# COMPLAX: A Python Tool Package for Automated Microsolvation of Organic Molecules

**DOI:** 10.1021/acs.jcim.6c02105

**Published:** 2026-10-09

**Authors:** Federica Lauria, Andrea Maranzana

**Affiliations:** Department of Chemistry, 9314 University of Turin, Via P. Giuria 7, Turin I-10125, Italy

## Abstract

Since the solvent choice drives organic reactivity, accurate computational modeling of explicit solute–solvent interactions remains crucial but preparation-intensive. We present COMPLAX, an open-source Python tool that automates the generation and optimization of targeted microsolvated clusters around highly localized active sites. Unlike unconstrained global optimizers, COMPLAX employs a rigid-core approach to preserve delicate solute geometries. To ensure unbiased spatial sampling, it couples a deterministic Fibonacci spherical lattice with three-dimensional rotational randomizations. Generated structures are refined via a two-stage xTB geometry optimization, utilizing a constrained pre-relaxation step to prevent unphysical solvent detachment. By automating conformer ranking and energy evaluations via a simple command-line interface, COMPLAX provides a reproducible, out-of-the-box solution for explicit microsolvation workflows.

## Graphical abstract

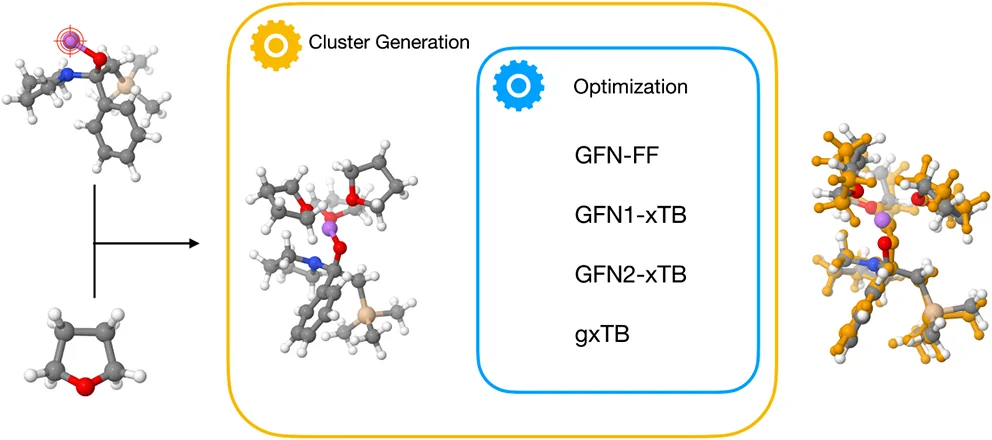

## 1 Introduction

In organic chemistry, the solvent actively dictates the course of the reaction, such as the reaction rates, yield, and chemo- or stereoselectivity, which can be deeply influenced by this specific solvent environment or even by trace amounts of certain coordinating species.^1,2^ This effect is particularly pronounced in the presence of charged species, such as metal ions, introducing the concept of the primary solvation shell.^3,4^ When simulating these phenomena computationally, solvent effects can be incorporated via mean-field continuum approximations;^5^ this category includes the Polarizable Continuum Model (PCM),^6^ the Conductor-like Screening Model (COSMO),^7^ and the Conductor-like Screening Model for Real Solvents (COSMO-RS).^8^ However, continuum models do not adequately describe specific solute–solvent interactions, such as hydrogen bonds or ion-molecule interactions.^9^ Alternatively, full explicit solvation via density functional theory (DFT) or running ab initio molecular dynamics (AIMD) is highly accurate but computationally demanding. A technique to reduce the computational cost of simulating a moderate amount of solvent molecules with good results is to use a hybrid approach that involves partitioning the system into several levels of calculation, applied to different parts of a molecule.^10^ On the contrary, a large number of solvent molecules is not always necessary; the use of both explicit and implicit solvent models is sufficient to provide a good estimate of the effect of interest.^11,12^

In the computational design of reactions, microsolvation has become a new focus for software capable of meeting these requirements. Several computational tools have been developed, although they often target different scopes. General-purpose conformational search algorithms—such as CREST,^13,14^ ABCluster,^15,16^ OGOLEM,^17^ and modules within quantum chemistry suites (e.g., GOAT^18^ and QCG in xTB^19,20^ )—excel at exploring vast configurational spaces. However, their unconstrained relaxations can unphysically distort delicate, user-defined solute geometries, such as transition states or highly directional metal coordinations. Conversely, tools dedicated to explicit solvation (e.g., Solvator in ORCA version,^21^ and PyConSolv^22^) typically generate bulk solvation boxes (e.g., a 20 Å bubble) using molecular dynamics (MD) or nondeterministic placement around the user-specified solute. To achieve this efficiency, these workflows are generally based on cost-effective strategies that go from classical MD to semiempirical extended tight-binding (xTB) metadynamics. Comparisons between COMPLAX and some of the above-mentioned software are reported in Section 3. Recently, xTB has quickly become a benchmark tool for conformational analysis, offering an excellent balance between quantum accuracy and computational speed. However, these tools are primarily designed for generalized intramolecular conformational studies or for generating unconstrained global clusters aimed at reproducing macroscopic solvent density. They lack a targeted, rigid-core protocol for coordinating explicit solvent molecules while strictly preserving the underlying solute geometry, often erasing the user’s initial structural intent and trapping the system in chemically irrelevant local minima. Consequently, the criteria for evaluating these approaches cannot be the same.

To bridge these methodological gaps, we introduce COMPLAX, a user-friendly software tool designed to automate the generation and optimization of targeted microsolvated clusters. COMPLAX avoids generalized bulk solvation by utilizing a fixed, deterministic spatial grid in combination with semiempirical techniques to conduct precise microsolvation around the specific active sites. For such targeted applications, the primary validation criteria prioritize structural fidelity to the input coordinates and the reduction of subsequent DFT computational costs rather than unconstrained global energy minimization.

The software proves to be useful for accurately calculating the energies of molecular systems, especially when the solvent plays a crucial role. This is particularly true in scenarios involving ion–molecule interactions, such as those between organic or metal ions and solvent molecules as well as the formation of hydrogen bonds.

COMPLAX is operated via a user-friendly command-line interface from input manipulation to output processing. By standardizing these steps and minimizing manual data handling, COMPLAX provides a robust, out-of-the-box solution for explicit solvent modeling and the exploration of conformational space.

## 2 Software Architecture and Implementation

### 2.1 Workflow

COMPLAX automates the preparation of microsolvated molecular systems, eliminating the need for manual manipulation via an external visualization software. It rapidly generates high-quality, preoptimized starting geometries for subsequent high-accuracy calculations (e.g., DFT). The entire workflow (Figure 1) is orchestrated via a command-line interface (CLI) in four steps: (i) input specification; (ii) generation of spatial distribution of the solvent via a nested systematic-stochastic algorithm (Fibonacci grid, isotropic 3D rotations, and vectorized steric checks); (iii) a two-stage geometry optimization using xTB; and (iv) automated output parsing, including energetic ranking and on-the-fly complexation energy evaluation. Implicit solvent models (ALPB, GBSA, or GBE)^23^ are also supported. A comprehensive list of CLI arguments and installation instructions is provided in the Supporting Information (Sections S1 and S2).

**Figure 1:**
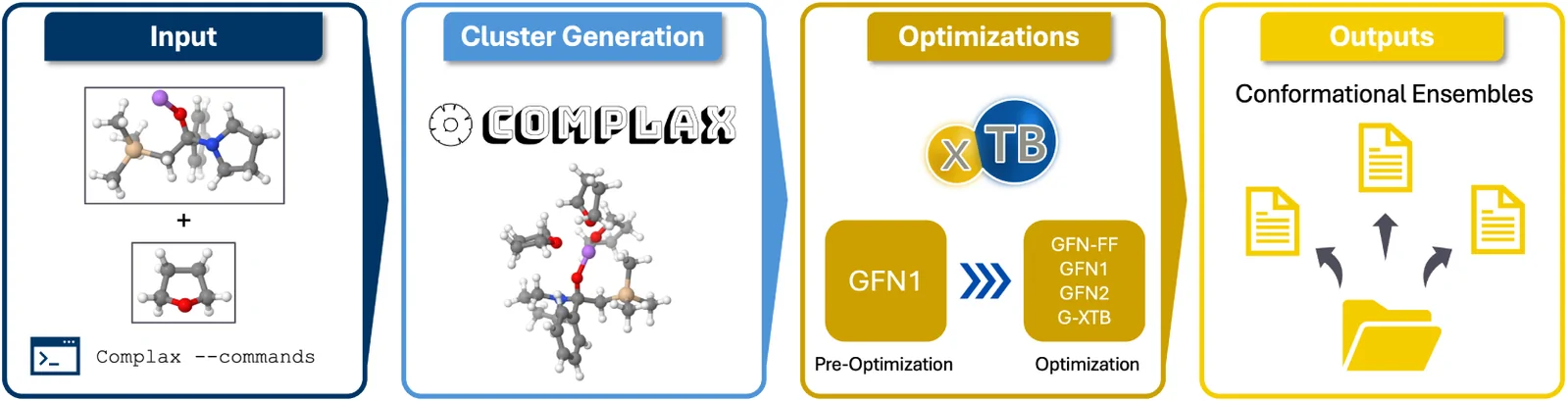
Schematic workflow of COMPLAX. Molecular structure figures were obtained by the Jmol program.^24^

Users must supply Cartesian coordinates for the solute and solvent (ideally DFT-optimized), the target interacting atom, and the desired number of solvent molecules. The workflow is highly customizable: optional parameters control the intermolecular target distance, Fibonacci grid resolution, stochastic configurations, and the number of top structures to retain. The pseudorandom generator can be initialized for complete spatial sampling reproducibility (benchmarked in Section S5.1). Furthermore, standard simulation parameters such as parallelization, total charge, unpaired electrons, and the specific xTB computational level (GFN2, GFN1, GFN-FF, and g-xTB^25^) are fully supported.

The positioning algorithm (detailed in Section 2.2) evaluates the coordination space using a deterministic Fibonacci lattice. For each grid point, the solvent was translated to the origin, randomly rotated in 3D, and mapped onto the target site coordinates. Vectorized steric checks ensure that initial clusters lack unphysical atomic overlaps.

To prevent artificial solvent detachment or severe deformations, the generated structures undergo two-stage parallel optimization. First, a rapid preoptimization (50 cycles with the GFN1-xTB level) is performed with solute frozen coordinates and harmonic distance constraints on both solvent focal points and internal bonds. Second, intermolecular constraints are lifted, allowing the solvent shell to fully relax around the rigid solute core at the user-specified computational level/method.

Finally, all outputs are parsed and stored in a dedicated “outplax” directory. COMPLAX can automatically rank the optimized structures by total energy and delete the intermediate files of discarded conformers to minimize storage overhead. Additionally, an automated summary report can calculate complexation energies (*ΔE*) by comparing the final cluster energies against the isolated components.

### 2.2 Implementation

COMPLAX is developed in Python 3.11 and distributed via the Python Package Index (pip install complax). External dependencies include xTB,^19,20^ NumPy,^26^ ASE,^27^ tabulate,^28^ tqdm,^29^ and Colorama.^30^

The generation of molecular configurations relies on spatial distribution, rigid-body rotation, and vectorized overlap screening to arrange solvent shells into collision-free, physically plausible geometries. While applying Fibonacci spherical lattices shares foundational similarities with the water cluster methodology pioneered by Kildgaard et al.,^31^ COMPLAX provides a generalized, highly automated evolution. Specifically, it is intrinsically designed to handle diverse organic molecules—including various solvents and counterions—while directly coupling structural generation with external quantum chemistry engines.

#### 2.2.1 Vector Normalization

This operation is essential for defining the directions and axes that are independent of the scale of the original vector. In the context of the code, normalization is used to generate random directions in three-dimensional space and to define normalized rotation axes within the *RandomRotationMatrix* function.

The normalize (*v*) function returns a unit vector obtained by dividing the input vector by its Euclidean norm:

$$\mathrm{v̂}=\frac{v}{\left|v\right|}=\frac{v}{\sqrt{x^{2}+y^{2}+z^{2}}}$$

#### 2.2.2 Deterministic Spatial Grid: The Fibonacci Sphere

To avoid the poor sampling efficiency typically associated with a pseudorandom directional vector in crowded environments, COMPLAX utilizes a deterministic Fibonacci spherical lattice to scan the coordination sphere. This method distributes points evenly along the vertical axis of the sphere (representing latitude or the *Z*-axis) and rotates them around the sphere’s horizontal axis (representing longitude or azimuth) based on the golden angle (Figure 2).

**Figure 2:**
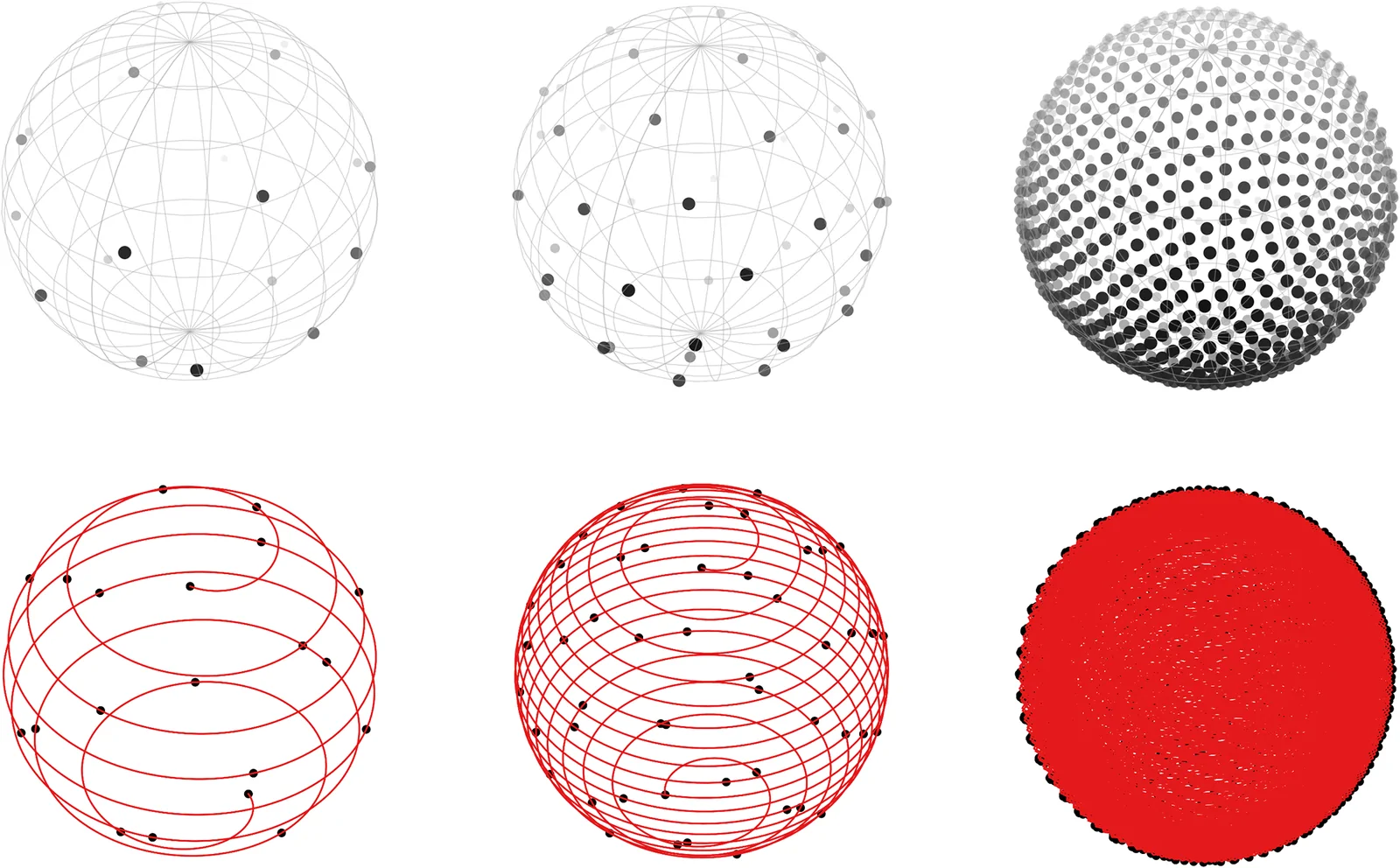
Deterministic spatial grid generation via the Fibonacci spherical lattice sampling algorithm implemented in COMPLAX. The top row illustrates the final discrete coordination nodes, whereas the bottom row depicts the corresponding generative spiral (θ_golden_ ≈ 137.5°). Columns from left to right show the scale-up of spatial resolution: low density (*N* = 20 points, left), medium density (*N* = 50 points, center), and high density (*N* = 1000 points, right).

COMPLAX distributes a user-defined number of points (*N*) across a spherical surface centered on the solute target atom. The coordinates are calculated using the Golden Ratio $\left(\Phi =\frac{1+\sqrt{5}}{2}\right)$ , introducing a constant irrational increment (the Golden Angle, θ_golden_ ≈ 137.5°) that prevents periodic alignment. To locate the point in 3D space, standard parametric equations for the Fibonacci lattice are employed (mathematical details are provided in the Supporting Information, Section S4.1).

Finally, to preserve sampling stochasticity across different runs, this deterministic directional grid was randomly shuffled prior to the placement loop. This ensures that the algorithm uniformly maps the available space without repeating identical trajectories across the independent structural batches.

#### 2.2.3 Isotropic 3D Rotational Randomization

Once a directional vector is selected from the shuffled Fibonacci grid, the incoming solvent molecule must be oriented. Rather than constraining the ligand to a simple axial rotation around the solute–solvent binding vector, COMPLAX samples the full three-dimensional rotational degrees of freedom within the *SO*(3) rotational group, treating the solvent as a free rigid rotor.

For every attempt, a completely random 3D rotation axis *û* (an isotropic unit vector) and a uniform rotation angle θ ∈ [0, 2π) are generated. The solvent coordinates—temporarily translated so that the focal atom sits at the origin—are rotated using a 3D rotation matrix constructed via the Euler-Rodrigues formula:^32^

$$R=I+\sin\theta K+\left(1-\cos\theta \right)K^{2}$$

where ***I*** is the 3 × 3 identity matrix and ***K*** is the skew-symmetric cross-product matrix:

$$R=\left(\begin{array}{lll} s_{x}^{2}+\left(1-s_{x}^{2}\right)c\theta & s_{x}s_{y}\left(1-c\theta \right)-s_{z}s\theta & s_{x}s_{z}\left(1-c\theta \right)+s_{y}s\theta \\ s_{x}s_{y}\left(1-c\theta \right)+s_{z}s\theta & s_{y}^{2}+\left(1-s_{y}^{2}\right)c\theta & s_{y}s_{z}\left(1-c\theta \right)-s_{x}s\theta \\ s_{x}s_{z}\left(1-c\theta \right)-s_{y}s\theta & s_{y}s_{z}\left(1-c\theta \right)+s_{x}s\theta & s_{z}^{2}+\left(1-s_{z}^{2}\right)c\theta \end{array}\right)$$

Applying ***R*** to the solvent coordinates ensures random rotation in space, optimizing the molecular orientation for insertion and significantly increasing packing efficiency for sterically demanding systems (full mathematical details and matrix derivations are provided in the Supporting Information, Section S4.2).

#### 2.2.4 Vectorized Steric Check

Every proposed solvent orientation undergoes a rigid-body, steric exclusion filter to eliminate unphysical configurations before quantum chemistry optimization. To handle the immense combinatorics of the nested grid search, interatomic distance evaluations between the proposed solvent and existing cluster components are entirely vectorized using *NumPy* broadcasting arrays, computing the full pairwise distance matrix in a single pass. Given the coordinate matrix of an existing cluster component **P**_**1**_ (of shape [*A*_1_, 3]) and the proposed solvent matrix **P**_**2**_ (of shape [*A*_2_, 3]), the full pairwise distance matrix is computed in a single vectorized pass as follows:

$$D_{jk}^{2}=\overset{c=1}{\underset{3}{\sum }}{\left(\left(P_{1}\right)\right)}_{j,c}-{\left(P_{2}\right)}_{k,c}{)}^{2}$$

The algorithm checks whether any pairwise squared distance falls below the user-defined threshold squared:

$$\exists \left(j,k\right)such\;that\;D_{jk}^{2}<d_{cutoff}^{2}$$

If a collision is detected, the inner loop samples a new random 3D orientation. This check acts as a geometric proxy for the Pauli core repulsion. Users can modulate the tightness of initial packing via the CLI (default: 1.6 Å). A slightly relaxed cutoff, combined with a high-density Fibonacci grid, allows crowded solvent shells to tightly pack, while the subsequent GFN1-xTB pre-optimization step smoothly resolves any minor structural friction.

## 3 Methodological Validation

To assess the structural fidelity of the geometries generated by COMPLAX, the lowest-energy outputs were benchmarked at the DFT level. As a primary test case, the global minima identified for a lithium compound explicitly solvated by 1,3 tetrahydrofuran (THF) molecules were re-optimized using the ORCA quantum chemistry package (version 6.1.0)^21^ under the same implicit solvation method. Further validation calculations on diverse chemical species (phenate, pyridine, acetate ion, palladium complexes) are detailed in Section S5 of the Supporting Information. These reference optimizations were performed at the ωB97X-D3 ^33^/def2-SVP^34^ level of theory, matching the computational setup of the isolated input structures. Some limited def2-TZVP optimizations were conducted and showed good agreement with def2-SVP geometries (see Supporting Information). Upon completion of the DFT geometry optimizations, the structural fidelity of the pre-optimized starting structures—generated at the unconstrained GFN2-xTB level following a strictly constrained GFN1-xTB initialization—was evaluated. These geometries accurately reproduced the relative energetic stabilization trends, requiring minimal structural rearrangement to reach the final DFT minima (Table 1, Figure 3). Full RMSD analyses are detailed in the Supporting Information.

**Table 1:** Comparison of Relative Final Energies (ΔE, kcal mol^–1^) and Required DFT Optimization Cycles (ωB97X-D3/def2-SVP) for Explicitly Solvated Clusters Generated by COMPLAX, CREST, Solvator, OGOLEM, and ABCluster^a^

Solvation State	COMPLAX opt. cycles	CREST (ΔE/Cycles)	Solvator (ΔE/Cycles)	OGOLEM (ΔE/Cycles)	ABCluster (ΔE/Cycles)
OrgLi + THF	181	+4.6/71	+24.5/124	+10.2/58	+1.8/37
Phenate + MeOH	33	+0.8/76	+5.1/93	+3.2/70	-0.7/35
Pyridine + H_2_O	27	0.0/213	+0.8/32	-0.1/66	-0.1/65
Pd + CH_3_CN	74	+5.7/47	0.0/63	0.0/37	+1.6/17
Acetate + H_2_O	113	+0.8/170	+12.2/64	+18.2/122	-4.7/27

a The ΔE values are relative to the COMPLAX-generated minima after DFT optimization (set as reference zero, ΔE = 0.0). Positive values indicate that the algorithm converged to a higher-energy local minimum. Complete structural RMSD values and solvator benchmark data are detailed in the Supporting Information.

**Figure 3:**
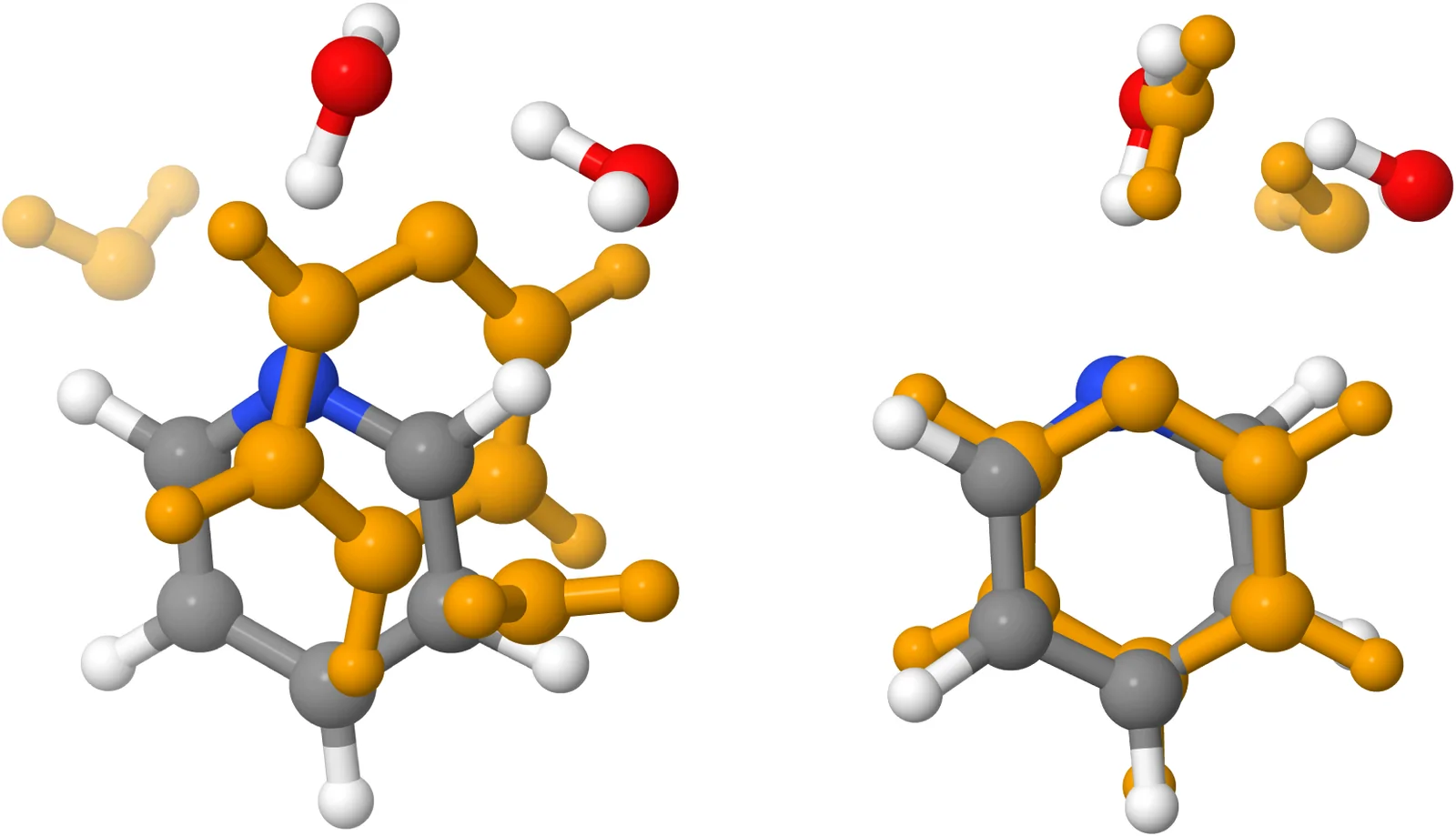
Superimposition of the initially generated structures (orange) and the final DFT-optimized geometries (standard CPK colors) for the pyridine·(H_2_O)_2_ complex, comparing the inputs from CREST (left) and COMPLAX (right). Images were generated by Jmol.^24^

To further validate COMPLAX, its workflow was directly compared against the ORCA Solvator module, CREST, OGOLEM, and the stochastic global optimizer ABCluster (SI Section S5.3), with COMPLAX set to generate 50 initial structures per system. All generated initial structures were subjected to full DFT optimization to evaluate their proximity to the final relaxed minima. Analysis of the results (Table 1) reveals that the performance of these methodologies strongly depends on the chemical nature of the system. For instance, ABCluster proved superior for highly flexible, electrostatically driven networks, locating a significantly deeper minimum for the acetate ion (−4.7 kcal mol^–1^) in fewer cycles, although comparable minima can be achieved with COMPLAX by increasing the number of generated stochastic structures. Similarly, OGOLEM demonstrated high efficiency in specific cases, reaching the same energetic minimum as COMPLAX for the Pd­(II) complex. Nonetheless, COMPLAX positions itself as a competitive tool across diverse chemical domains, significantly outperforming most tested methods in challenging, highly directional coordination networks. In the organolithium complex, for example, unguided algorithms easily became trapped in high-energy local minima (e.g., +10.2 kcal mol^–1^ for OGOLEM and +24.5 kcal mol^–1^ for Solvator). Furthermore, COMPLAX ensures superior computational efficiency even when other tools identify comparable minima: in the pyridine-water complex, COMPLAX reached convergence in only 27 cycles, compared to 65 for ABCluster, 66 for OGOLEM, and 213 for CREST. This severe discrepancy for CREST arises because its unconstrained search positioned water along the aromatic π-system rather than the nitrogen lone pair, forcing a costly structural rearrangement (Figure 3). While ABCluster and OGOLEM emerged as the most competitive alternative methodologies for standard ground-state solvation, their reliance on unconstrained explorations makes them unsuited for preserving delicate user-defined geometries, such as transition states. In such critical applications, the lack of native geometric restraints in these global optimizers would inevitably distort the active site. By integrating a fully automated rigid-core protocol, COMPLAX provides a reliable solution for ensuring the microsolvation of molecules with different types of intermolecular interactions. Further geometric validations, including triple-zeta basis set comparisons, are provided in the Supporting Information (Section S5).

## 4 Conclusion

In conclusion, COMPLAX introduces an automated computational framework for generating microsolvated clusters around target active sites, bypassing manual structural manipulation. By coupling a deterministic Fibonacci spherical lattice with isotropic 3D rotational randomizations and dynamic steric screening, it ensures an unbiased exploration of the coordination space. Its integrated two-stage optimization and automated parsing allow users to extract energetically ranked conformers and evaluate cooperative solvation effects. Furthermore, its rigid-core methodology prevents artificial deformations intrinsic to unconstrained global searches, making it uniquely suited for systems where localized solvent effects—such as those in organometallics, directional metal coordination, or transition states—demand strict structural preservation.

Future developments will interface COMPLAX with alternative geometry optimization engines, including emerging machine-learning interatomic potentials (MLIPs). Upcoming modules will also support the concurrent solvent mixtures and molecular aggregation workflows and generalize input/output parsing for broader chemical file formats.

## Supplementary Material



## Data Availability

COMPLAX is available under the MIT License and can be installed via PyPI. The source code, documentation, and examples are hosted at https://github.com/Fedelau/complax. Version v1.1.1 was used for the benchmarks and results reported in this manuscript.

## Notes

Installation and setup, command-line reference, examples of workflow, and supplementary validation data (PDF)

## Abbreviations

CLI: Command-line interface
DFT: Density functional theory
AIMD: *Ab initio* molecular dynamics
MLIPs: Machine learning interatomic potentials
PCM: Polarizable Continuum Model
COSMO: Conductor-like screening model
COSMO-RS: Conductor-like screening model for real solvents
