# Supplementary material for: COMPLAX: A Python Tool Package for Automated Microsolvation of Organic Molecules
Source: J Chem Inf Model. 2026 Oct 9;66(20):13050–5. doi: 10.1021/acs.jcim.6c02105 (PMC13715514; doi:10.1021/acs.jcim.6c02105)
Supplement: Supplementary file 1 [file ci6c02105_si_001.pdf]

Supporting Information

COMPLAX: A Python Tool Package for Automated Microsolvation of Organic Molecules

Federica Lauria and Andrea Maranzana

Department of Chemistry, University of Turin, Via Pietro Giuria, 7, I-10125, Torino, Italy

S1 Table of Contents

S1 INSTALLATION AND SETUP **S3**

S1.1 PYTHON ENVIRONMENT SETUP S3

S1.2 INSTALL THE PACKAGE S3

S1.3 REQUIREMENTS S3

S1.4 EXTERNAL SOFTWARE (XTB) S4

S2 CLI REFERENCE **S4**

S2.1 MANDATORY POSITIONAL ARGUMENTS S4

S2.2 SOLVATION AND GEOMETRIC PARAMETERS S4

S2.3 CONFORMATIONAL SAMPLING AND ANALYSIS S5

S2.4 SEMIEMPIRICAL (XTB) SETTINGS S6

S3 EXAMPLE WORKFLOW **S6**

S3.1 LAUNCHING THE CALCULATION S7

S3.2 COMMAND BREAKDOWN S7

S3.3 EXPECTED OUTPUT S8

S3.4 GUIDELINES FOR PARAMETER SELECTION S9

S4 MATHEMATICAL DETAILS **S10**

S4.1 FIBONACCI SPHERICAL LATTICE EQUATIONS S10

S4.2 EULER-RODRIGUES ROTATION MATRIX S11

S5 SUPPLEMENTARY VALIDATION DATA **S11**

S5.1 DFT BENCHMARKING OF THE CONFORMATIONAL SAMPLING S11

S5.2 COMPARISON WITH TRIPLE ZETA BASIS SET S14

S5.3 WORKFLOW EFFICIENCY AND TOOL COMPARISON S15

S5.4 REPRODUCIBILITY AND STOCHASTICITY IN CLUSTER GENERATION S19

S6 CARTESIAN COORDINATES **S21**

S7 REFERENCES **S83**

S1 Installation and Setup

COMPLAX is a Python-based command-line tool designed to act as a high-level driver for automated microsolvation and semiempirical optimizations. Therefore, a complete installation involves setting up the required Python environment and ensuring that the external engine is correctly installed and linked.

S1.1 Python Environment Setup

The software requires Python 3.8 or higher. We highly recommend running COMPLAX within a virtual environment to manage dependencies and avoid conflicts with pre-existing system libraries:

```
# Create a new python environemnt named "complaxvenv"
python -m complaxvenv /path/to/virtual/environment
# Activate the environment
source /path/to/virtual/environment/bin/activate
```

S1.2 Install the package

COMPLAX can be installed directly from PyPI using pip:

```
pip install complax
```

Alternatively, it is possible to install the tool manually by cloning the official repository on Github:

```
git clone https://github.com/Fedelau/complax.git
```

S1.3 Requirements

To endure fully comparability and handle generation of atomic coordinates, spatial grid, and terminal outputs, the following standard third-party Python libraries must be installed:

- `numpy` for matrix operations and spatial grid generation;[1]
- `ase` Atomic Simulation Environment, for molecular file parsing and manipulation;
- `colorama` for cross-platform-colored terminal outputs;[2]
- `tabulate` for formatting the final solvation energy tables;[3]
- `tqdm` for rendering the parallel execution progress bar.[4]

If COMPLAX is installed via PyPI, these requirements are resolved and downloaded automatically. For manual GitHub installations, these dependencies can be easily installed via the standard Python package manager using the command:

```
pip install -r requirements.txt
```

S1.4 External Software (xTB)

COMPLAX relies on the Grimme group's extended tight-binding program (xTB)[5] to perform all semiempirical calculations and geometry optimizations. Users must download and install the xTB standalone binary from its official repository (https://github.com/grimme-lab/xtb).

For COMPLAX to successfully execute the semiempirical tasks, the `xtb` executable must be globally accessible. Users must ensure that the installation directory of xTB is permanently added to their system's environmental `PATH` variable.

S2 CLI Reference

COMPLAX is executed via terminal using a single, unified command string. The general syntax for launching a calculation requires two mandatory positional arguments (Cartesian coordinates of the starting molecules) followed by a series of optional flags to customuze the workflow:

```
complax <solute.xyz> <solvent.xyz> [options]
```

The following table summarizes all available command-line arguments, grouped by functionality.

S2.1 Mandatory Positional Arguments

**Argument**	**Description**
`file1`	Path to the `.xyz` file containing the cental solute molecule
`file2`	Path to the `.xyz` file containing the solvent molecules to be added

S2.2 Solvation and Geometric Parameters

Argument	Default	Description
`-c`	1	Number of solvent molecules to dynamically add around the solute

`-t`	`2.0`	Target distance (in Å) between the selected anchoring atoms
`-a`	`None`	Indices (1-based) of the two atoms (solute and solvent) to be kept at distance -t. Example: -a 5 1 connects atom 5 of the solute and atom 1 of the solvent(s)
`--cutoff`	`1.6`	Steric overlap distance threshold (in Å) for the collision screening
`--res`	`10000`	Number of points in the Fibonacci spherical grid used for spatial sampling

S2.3 Conformational Sampling and Analysis

Argument	Default	Description
`--nstruct`	`400`	Number of independent stochastic structural configurations to generate
`--seed`	`42`	Pseudo-random number generator (PRNG) seed for strict reproducibility. Set to `-1` for purely stochastic exploration based on the system clock
`--keep-best`	`None`	Deletes higher-energy conformers and keeps only the top N structures
`--solvfx`	`False`	Calculates and prints a detailed breakdown table of the complexation and solvation energies

S2.4 Semiempirical (xTB) Settings

Argument	Default	Description
`--lev`	`gfn2`	xTB Hamiltonian level (`gfn0`, `gfn1`, `gfn2`, `gfnff`, `gxtb`)
`--charg`	`0`	Total molecular charge of the complete simulated system
`-u, --uhf`	`0`	Number of unpaired electrons (e.g., `1` for a radical doublet)
`-p`	`1`	Number of CPU cores allocated for parallel execution
`--alpb`	`None`	Activates the ALPB implicit continuum solvent model (e.g., `--alpb water`)
`--gbsa`	`None`	Activates the GBSA implicit continuum solvent model
`--gbe`	`None`	Activates the GBE implicit model (only compatible with `--lev gxtb`)

S3 Example Workflow

To illustrate the standard COMPLAX workflow, we provide a practical example simulating the targeted microsolvation of a specific active site. In this scenario, we aim to explicitly coordinate three tetrahydrofuran molecules (`thf.xyz`) by their oxygen atom around the lithium atom of

methylithium (`lime.xyz`) (Figure S1), while embedding the entire system within an implicit continuum solvent.

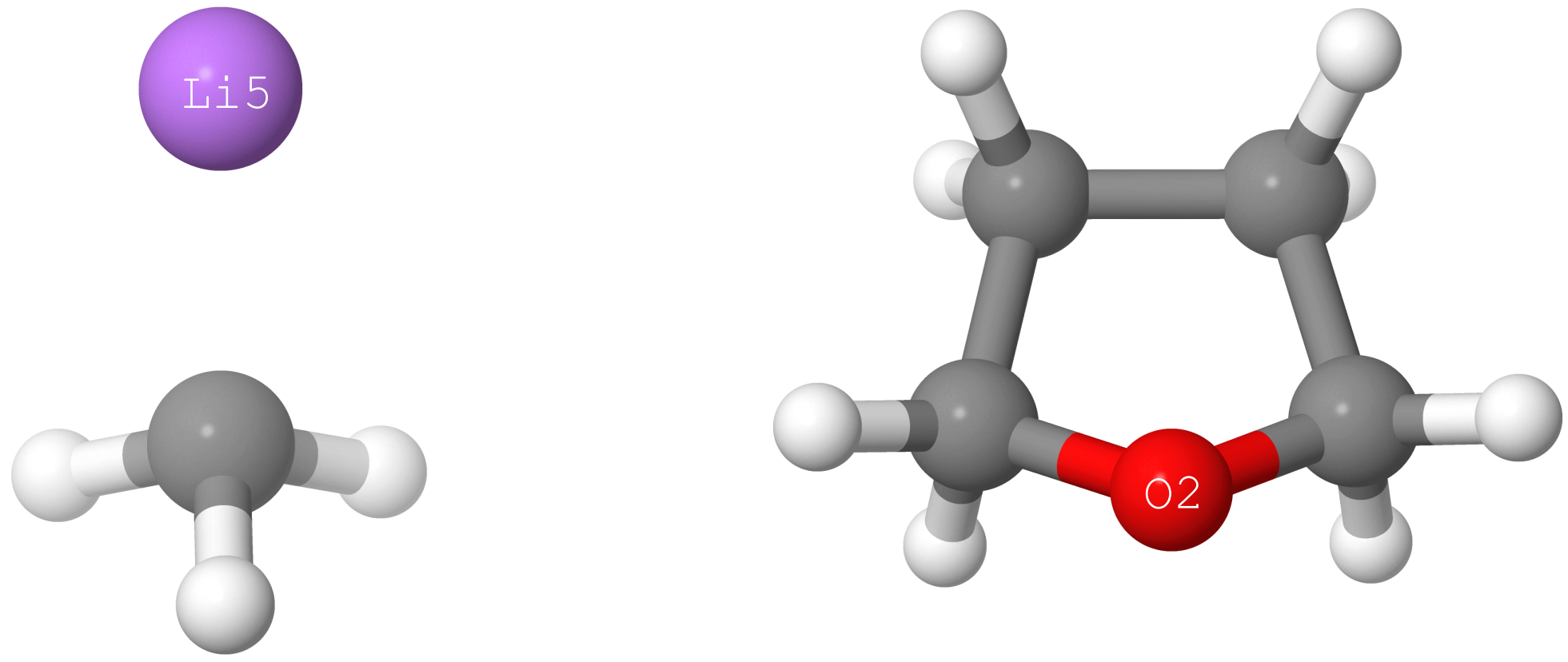

Figure S1: On the left, the methyllithium molecule, where the lithium atom (Atom 5) was chosen as the target. On the right, the tetrahydrofuran (THF) solvent molecule, where the oxygen atom (Atom 2) was selected to coordinate with the lithium center.

S3.1 Launching the Calculation

Assuming both coordinate files are present in the working directory, the user can launch the automated conformational sampling and optimization by executing the following command in the terminal:

```
complax lime.xyz thf.xyz -a 5 2 -c 3 --nstruct 20 --keep-best 3 --alpb thf -solvfx
```

S3.2 Command Breakdown

The complete explanation of the selected flags is present herein:

- `-a 5 2`: Anchors Atom 5 of the solute (e.g., the lithium atom) to Atom 2 of the solvent (e.g., the tetrahydrofuran oxygen).
- `-c 3`: Iteratively adds three explicit solvent molecules.
- `--nstruct 20`: Generates an ensemble of 20 stochastic starting configurations to ensure an exploration of the local conformational space.
- `--keep-best 3`: Automatically deletes the higher-energy conformers, keeping only the top 3 most stable optimized structures on the folder.
- `--alpb thf`: Evaluates the background electrostatic screening using the ALPB model parameterized for thf.
- `--solvfx`: Prompts the program to compute and display the final thermodynamic solvation effect table.

S3.3 Expected Output

During the execution, the CLI provides expected time feedback on the estimated completion time via a dynamic progress bar. Once the parallel optimizations are complete, COMPLAX automatically parses the xTB output files, identifies the lowest-energy conformers, and prints a final ranking summary directly to the terminal:

```
----------------------------------------------------------------

---------- Geometry optimization of 60 structure(s) ----------------

----------------------------------------------------------------

Calculating ...: 100%|█████████████████████████| 60/60 [Elapsed Time:00:43 ETA:00:00]


------------------------------------------------

        Ranking of stochastic solvent structures

------------------------------------------------


[1 Solvent Molecule(s)]

complax_struct_1solvent_20            -20.635510 Eh

complax_struct_1solvent_10            -20.635401 Eh

complax_struct_1solvent_9             -20.635312 Eh

...

[2 Solvent Molecule(s)]

complax_struct_2solvent_13            -37.368223 Eh

complax_struct_2solvent_17            -37.367507 Eh

complax_struct_2solvent_7             -37.366924 Eh

...

[3 Solvent Molecule(s)]

complax_struct_3solvent_5             -54.092922 Eh

complax_struct_3solvent_11            -54.092893 Eh

complax_struct_3solvent_17            -54.092855 Eh
```

Furthermore, since the evaluation of the thermodynamic solvation effect was requested (via the `--solvfx` flag), a detailed energy breakdown table is also displayed in the terminal:

```
                        **************************
                        * Effect of the Solvation *
                        **************************


+------------------------------------+------------+------------+------------+
|                                    |          1 |          2 |          3 |
+====================================+============+============+============+
| Sum of reag + solv (Hartree)       | -20.609217 | -37.322501 | -54.035784 |
+------------------------------------+------------+------------+------------+
| Tot Energy (Complex) (Hartree)     | -20.635312 | -37.366924 | -54.092922 |
+------------------------------------+------------+------------+------------+
| ΔE (kcal/mol)                      | -16.37     | -27.87     | -35.85     |
+------------------------------------+------------+------------+------------+


All the results have been saved in the ‘outplax’ folder.
```

Finally, all the desired optimized structures are automatically saved in the newly generated `outplax` directory.

S3.4 Guidelines for Parameter Selection

While COMPLAX provides robust default parameters optimized for standard organic interactions, specific chemical environments require a tailored selection of the `-t` (target) and `--cutoff` flags to ensure physically meaningful solvent placement.

- Target Selection (`-t`): The choice of the target atom(s) is highly dependent on the nature of the active site. When the target involves a transition metal or a highly charged species, users should carefully consider the metal's formal charge, oxidation state, and expected coordination geometry (e.g., square planar vs. tetrahedral). Selecting the appropriate metal center, or its immediately adjacent coordinating atoms, ensures that the algorithm correctly samples the primary coordination sphere and captures highly directional dative bonds.
- Distance Cutoff (`--cutoff`): This parameter defines the spatial boundary and placement threshold around the target. Its optimal value varies significantly based on the atomic radii and the dominant non-covalent interactions. For compact, electrostatically driven hydrogen-bonding networks, a tighter cutoff is generally sufficient. Conversely, when coordinating large

metal cations or dealing with diffuse ion-dipole interactions, the cutoff should be appropriately expanded to accommodate longer metal-ligand bond distances and larger van der Waals radii, preventing unphysical steric clashes during the initial placement phase.

To determine the most appropriate `--cutoff` value, users are encouraged to reference typical interaction distances from prior computational studies or experimental data. Alternatively, a rapid preliminary screening can be performed by reducing the number of generated structures via the `--nstruct` flag (which defaults to 400). Generating a small subset of configurations allows for a quick visual inspection of the initial geometries, ensuring that the spatial sampling is chemically reasonable before committing to a full production run.

S4 Mathematical Details

S4.1 Fibonacci Spherical Lattice Equations

The deterministic spatial grid in COMPLAX is generated by distributing a user-defined number of points N (passed via *--res* parameter) evenly across a spherical surface centered on the solute target atom. The Cartesian coordinates of each i-th point (where $i \in [0, N - 1]$) are calculated using the Golden Ratio ($\Phi = \frac{1+\sqrt{5}}{2}$), which introduces a constant irrational increment (the Golden Angle, $\theta_{golden} \approx 137.5°$), preventing periodic alignment. To locate the i-th point in three-dimensional space with a unit radius ($R = 1$), the following equations were used:

$$y = 1 - \frac{2i}{N - 1}$$

$$r = \sqrt{1 - y^2}$$

$$x = r \cdot cos(\phi_{rad})$$

$$z = r \cdot sin(\phi_{rad})$$

Where y is the height of the point along the vertical axis; r is the radius of the circular cross-section at the specific height; Φ is the angle of rotation about the vertical axis, expressed in radians. This collection of points provides an evenly distributed grid of the target directions. To preserve the stochasticity of the sampling across different runs, this grid of directional vectors was randomly

shuffled using a pseudo-random permutation algorithm (*np.random.shuffle*) prior to the placement loop.

S4.2 Euler-Rodrigues Rotation Matrix

To sample the full three-dimentional rotational degrees of freedom (the SO(3) rotational group), COMPLAX treats the incoming solvent molecule as a free rigid rotor. For each attempt, a completely random 3D rotation axis $\hat{\boldsymbol{u}}$ is generated as an isotropic unit vector, and a random rotation angle θ is sampled uniformly from the interval $[0, 2\pi)$. The coordinates of the solvent molecule, temporarily translated so that its focal atom sits at the coordinate origin, are then rotated using a 3D rotation matrix constructed via the Euler-Rodrigues formula[28]:

$$\boldsymbol{R} = \boldsymbol{I}\cos\theta + (1 - \cos\theta)\hat{\boldsymbol{u}}\hat{\boldsymbol{u}}^T + [\hat{\boldsymbol{u}}]_\times \sin\theta$$

Where $\boldsymbol{I}$ is the 3×3 identify matrix and $\hat{\boldsymbol{u}}\hat{\boldsymbol{u}}^T$ is the outer product or tersor product of two vectors. The skew-symmetric matrix $\boldsymbol{K}$ has the following property:

$$\boldsymbol{K}_s\boldsymbol{K}_s = \hat{\boldsymbol{u}}\hat{\boldsymbol{u}}^T - \boldsymbol{I}$$

The above Euler-Rodrigues formula can be rewritten in standard form as

$$\boldsymbol{R} = \boldsymbol{I} + \boldsymbol{sin}\,\theta\,\boldsymbol{K}_s + (\boldsymbol{1} - \boldsymbol{cos}\,\theta)\boldsymbol{K}^2$$

This standard form of the formula has been used to construct a rotation matrix from the axis-angle representation of rotation. Thus, the rotation matrix can be obtained as

$$R = \begin{pmatrix} s_x^2 + (1 - s_x^2)c\theta & s_x s_y(1 - c\theta) - s_z s\theta & s_x s_z(1 - c\theta) + s_y s\theta \\ s_x s_y(1 - c\theta) + s_z s\theta & s_y^2 + (1 - s_y^2)c\theta & s_y s_z(1 - c\theta) - s_x s\theta \\ s_x s_z(1 - c\theta) - s_y s\theta & s_y s_z(1 - c\theta) + s_x s\theta & s_z^2 + (1 - s_z^2)c\theta \end{pmatrix}$$

S5 Supplementary Validation Data

S5.1 DFT Benchmarking of the Conformational Sampling

To validate the reliability of the semi-empirical conformational sampling, density functional theory (DFT) calculations were performed on explicitly solvated test systems (pyridine-water and phenate-methanol clusters). For each system, an initial conformational search was conducted by generating 50 stochastic solvent configurations, which were subsequently optimized at the GFN2-xTB level. The

three lowest-energy conformers from this preliminary screening were then selected and subjected to full geometry optimization using the ωB97X-D3[6] functional in conjunction with the def2-SVP basis set.[7] with Orca version 6.1.0.[8] To quantitatively assess the geometric accuracy of the semi-empirical pre-optimization, the Root Mean Square Deviation (RMSD) of the atomic coordinates was calculated between the xTB minima and their corresponding DFT-optimized structures (Table S1). The resulting low RMSD values confirm that the semi-empirical stochastic screening provides robust and highly accurate starting geometries for high-level electronic structure calculations (Figure S2).

Table S1. DFT benchmarking of the conformational sampling for phenate-methanol, pyridine-water, organolithium-tetrahydrofuran, palladium complex-acetonitrile, and acetate anion-water clusters. For each system, the three lowest-energy conformers generated by the xTB pre-screening with 1, 2, 3 or 4 explicit solvent molecules were re-optimized at the ωB97X-D3/def2-SVP level. Structure labels (X.Y) denote the number of solvent molecules (X) and the energetic rank from the semi-empirical sampling (Y). The table reports the complexation energy (*ΔE*, in kcal mol^{-1}), the Root Mean Square Deviation (RMSD, in Å) between the xTB-generated starting geometries and the final DFT minima, and the total number of DFT optimization cycles required to reach convergence.

STRUCTURE	ΔE	RMSD	OPTIMIZATION CYCLES
**PHENATE-METHANOL**			
1.1	-3.7	0.50	28
1.2	-3.7	0.52	26
1.3	-3.4	0.32	24
2.1	-5.3	0.94	50
2.2	-5.4	0.77	46
2.3	-5.0	0.38	33
3.1	-7.6	0.52	33
3.2	-9.2	1.11	51
3.3	-8.1	0.97	48
**PYRIDINE-WATER**			
1.1	-1.8	0.44	28
1.2	-1.9	0.46	26
1.3	-1.8	0.47	27
2.1	-5.8	0.83	27

2.2	-7.0	1.06	51
2.3	-6.9	1.22	66
**ORGANOLITHIUM-THF**			
1.1	-4.7	1.04	37
1.2	-4.1	0.96	43
1.3	-4.3	1.3	63
2.1	-4.7	0.33	24
2.2	-7.6	0.69	23
2.3	-4.6	0.48	26
3.1	-19.3	1.25	181
3.2	-7.0	0.42	44
3.3	-14.2	1.37	135
**PALLADIUM COMPLEX-ACETONITRILE**			
1.1	-18.2	1.90	74
1.2	-18.2	1.88	62
1.3	-18.5	1.95	80
**ACETATE - WATER**			
1.1	-2.8	0.30	17
1.2	-3.6	0.62	27
1.3	-3.6	0.50	34
2.1	-4.5	0.45	32
2.2	-10.2	1.10	66
2.3	-6.6	0.56	35
3.1	-7.5	1.11	81
3.2	-7.8	0.66	79
3.3	-8.8	0.93	92
4.1	-20.6	0.53	113
4.2	-8.3	0.48	102
4.3	-11.7	1.14	85

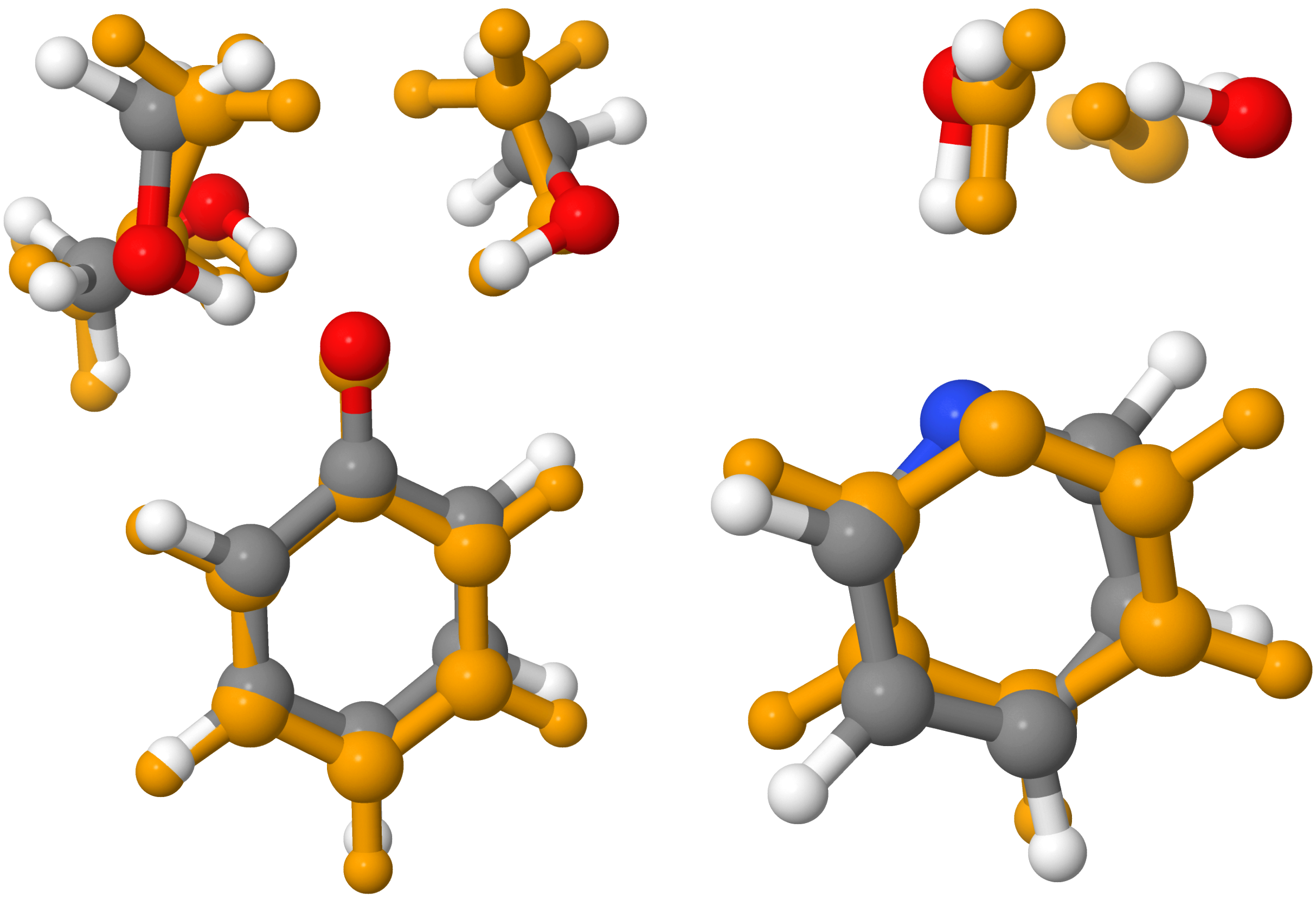

Figure S2. Visual validation of the conformational sampling for reference clusters. Structural overlays of the initial geometries generated by COMPLAX (solid orange) and the corresponding final DFT-optimized minima at the ωB97X-D3/def2-SVP level (standard CPK colors). Left: Phenate explicitly solvated by three methanol molecules. Right: Pyridine explicitly solvated by two water molecules. The high degree of structural overlap visually confirms the low RMSD values reported in Table S1.

S5.2 Comparison with triple zeta basis set

To further assess the geometric stability and basis set dependency of the optimized structures, the lowest-energy conformers identified for the 1-, 2-, and 3-solvent clusters were subjected to re-optimization using a larger triple-zeta basis set. Specifically, the global minima previously optimized at the ωB97X-D3/def2-SVP level were re-optimized at the ωB97X-D3/def2-TZVP[7] level. A structural comparison between the two levels of theory reveals a high degree of structural conservation, yielding Root Mean Square Deviation (RMSD) values of 0.12, 0.40, and 1.29 Å for the clusters containing one, two, and three solvent molecules, respectively. These values confirm that the structural parameters are already well-converged at the double-zeta level, and the expansion of the basis set does not substantially alter the resulting conformational geometries (Figure S3).

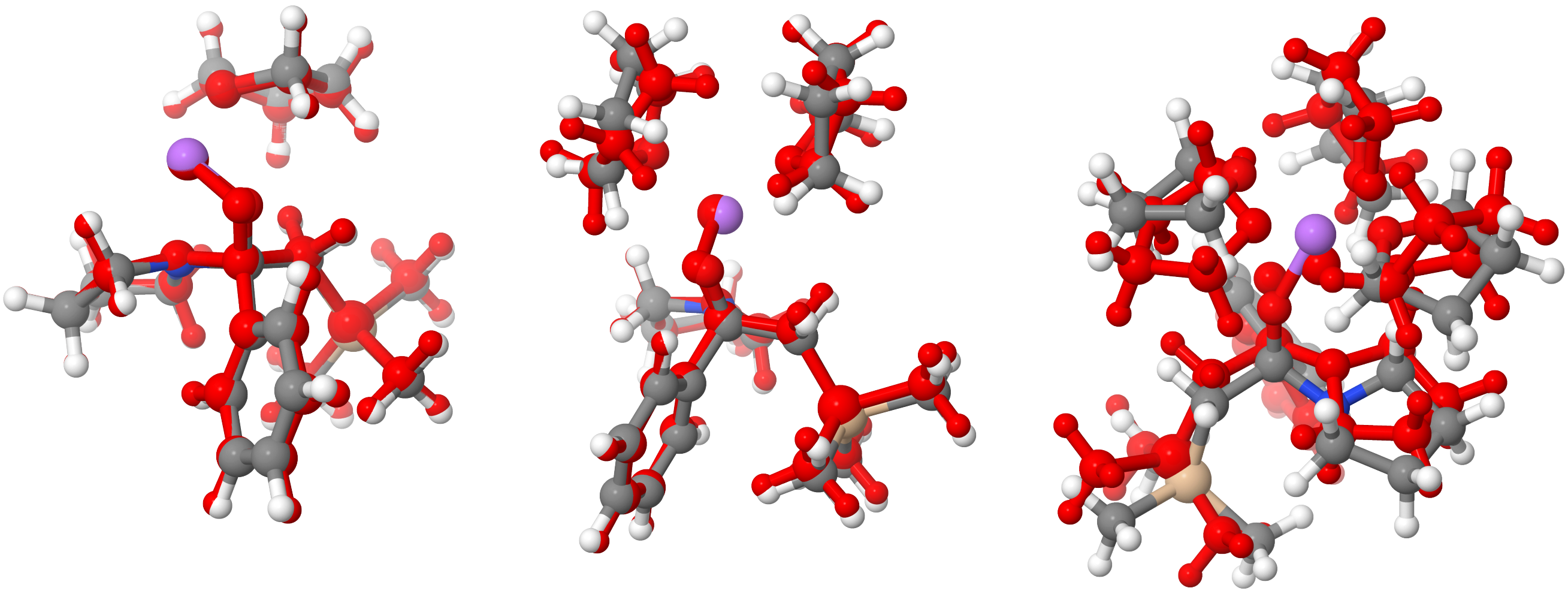

Figure S3. 3D structural overlay of the optimized 1-solvent cluster (left), 2-solvent cluster (center), and 3-solvent cluster (right). The reference DFT geometry (optimized at the ωB97X-D3/def2-SVP level) is depicted in standard CPK colors, whereas the geometry optimized at ωB97X-D3/def2-TZVP level is shown in solid red.

S5.3 Workflow Efficiency and Tool Comparison

To evaluate the capability of generating chemically sound starting structures for specific non-covalent interactions, the COMPLAX placement algorithm was directly compared with both the Solvator module implemented in the ORCA suite, the unconstrained conformational search algorithm of CREST, OGOLEM, and the stochastic global optimizer ABCluster. The initial geometries generated by all tools were subjected to full DFT optimization at the ωB97X-D3/def2-SVP level.

As reported in Table S2, the performance of the tools strongly depends on the nature of the molecular interaction. COMPLAX consistently identifies the most stable interaction modes for highly directional and complex environments, such as the metal coordination in the organolithium complex and the Pd(II) species, outperforming most other algorithms. In such highly directional systems, unconstrained optimizers often struggle: for instance, on the organolithium complex, Solvator, OGOLEM, and CREST converged to significantly higher-energy local minima (+24.5, +10.2, and +4.6 kcal mol^{-1}, respectively). Conversely, for highly flexible, electrostatic-driven hydrogen-bonded networks (e.g., acetate ion and phenate with water/methanol), the exhaustive stochastic sampling of ABCluster located deeper configurational minima (-4.7 and -0.7 kcal mol^{-1}, respectively). In these scenarios, OGOLEM exhibited inconsistent behavior, finding a competitive minimum for the pyridine-water complex (-0.1 kcal mol^{-1}) but failing severely on the acetate ion (+18.5 kcal mol^{-1}). Nevertheless, COMPLAX remains highly competitive in these scenarios, successfully placing solvents in highly relevant configurational basins without the need for the computationally expensive configurational screening intrinsic to global optimizers. Solvator struggled to correctly orient the

solvent molecules for highly directional interactions, converging to significantly higher-energy local minima, expect for Pd(II) species (Figure S4).

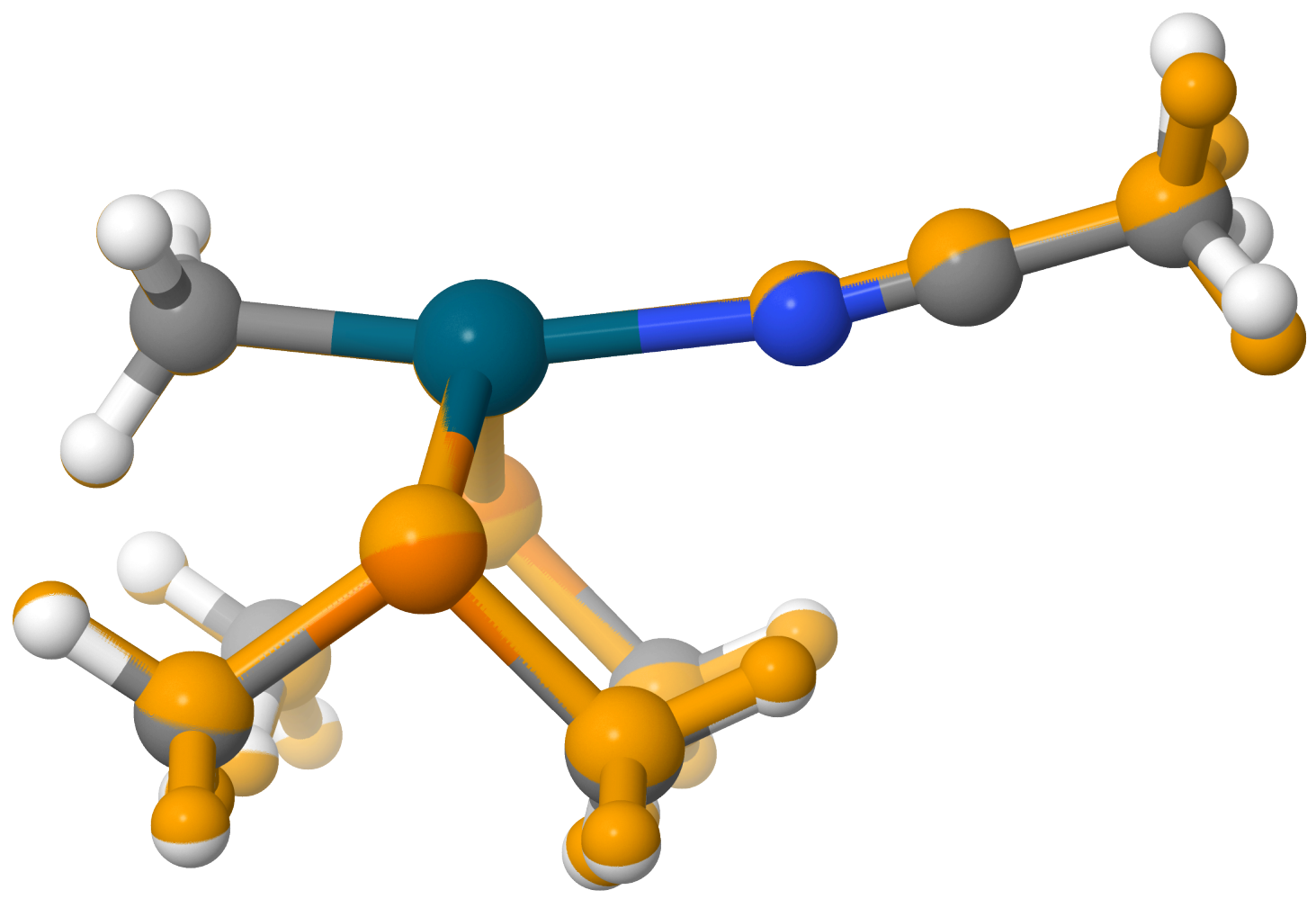

Figure S4. Comparison between the COMPLAX-generated structure (in standard CPK colors) and the Solvator-generated structure (in orange) after full DFT optimization.

While CREST successfully placed the solvent in relevant configurational areas, it frequently converged to less stable local minima compared to the targeted placement of COMPLAX (e.g., +4.6 kcal mol⁻¹ for the organolithium complex).

Table S2. Heatmap of final DFT potential energies (Hartree) and relative energies (ΔE, kcal mol^{-1}) for the microsolvated systems after full geometry optimization (ωB97X-D3/def2-SVP) starting from the structures generated by COMPLAX, CREST, ORCA Solvator, ABCluster, and OGOLEM. The lowest energy structure for each system is set as the reference point (ΔE = 0.0*). Negative values indicate that a deeper energetic minimum was found compared to the COMPLAX-generated reference structure (color gradients range from green for the lowest/most favorable values to red for the highest).

Molecular System	Solvent Molecules	Tool	Potential Energy (Hartree)	ΔE (kcal mol^{-1})
Phenate + MeOH	3	COMPLAX	-653.4798793	0.0*
		CREST	-653.478631	+0.8
		SOLVATOR	-653.4717308	+5.1
		ABCluster	-653.4810181	-0.7
		OGOLEM	-653.4748239	+3.2
Pyridine + H_2O	2	COMPLAX	-400.7464735	0.0*
		CREST	-400.7466514	0.0

		SOLVATOR	-400.7451732	+0.8
		ABCluster	-400.7466443	-0.1
		OGOLEM	-400.7466446	-0.1
OrganoLi + THF	3	COMPLAX	-1709.100769	0.0*
		CREST	-1709.093497	+4.6
		SOLVATOR	-1709.061686	+24.5
		ABCluster	-1709.097955	+1.8
		OGOLEM	-1709.084482	+10.2
Pd(II) + CH_3CN	1	COMPLAX	-1142.190437	0.0*
		CREST	-1142.18138	+5.7
		SOLVATOR	-1142.190427	+0.0
		ABCluster	-1142.187901	+1.6
		OGOLEM	-1142.190415	0.0
Acetate Ion + H_2O	4	COMPLAX	-533.7674297	0.0*
		CREST	-533.7661071	+0.8
		SOLVATOR	-533.7480375	+12.2
		ABCluster	-533.7748486	-4.7
		OGOLEM	-533.7379841	+18.5

Beyond identifying deeper configurational minima, COMPLAX generally improves computational efficiency during the quantum mechanical relaxation. As shown in Table S3 the pre-optimized structures generated by COMPLAX required significantly fewer DFT optimization cycles to reach convergence, maintaining a lower structural RMSD compared to CREST and OGOLEM in several test cases (e.g., the phenate and pyridine complexes). This highlights the advantage of the COMPLAX rigid-core approach: by preventing artificial solute deformations during the initial placement and pre-optimization stages, it provides highly accurate starting structures that are closer to the final DFT minima.

Table S3. Heatmap of DFT optimization cycles (ωB97X-D3/def2-SVP) and structural RMSD (initial vs. optimized geometry) for systems prepared with COMPLAX, CREST, SOLVATOR, ABCluster, and OGOLEM (color gradients range from green for the lowest/most favorable values to red for the highest).

Molecular system	Solvent Molecules	Tool	DFT Cycles	RMSD (Start vs Opt) (Å)

Phenate + MeOH	3	COMPLAX	33	0.52
		CREST	76	1.28
		SOLVATOR	93	2.25
		ABCluster	35	0.55
		OGOLEM	70	1.78
Pyridine + H_2O	2	COMPLAX	27	0.83
		CREST	213	3.08
		SOLVATOR	32	0.46
		ABCluster	65	0.18
		OGOLEM	66	0.18
OrganoLi + THF	3	COMPLAX	181	1.25
		CREST	71	0.66
		SOLVATOR	124	1.00
		ABCluster	37	0.47
		OGOLEM	58	0.63
Pd(II) + CH_3CN	1	COMPLAX	74	1.9
		CREST	47	1.54
		SOLVATOR	63	1.84
		ABCluster	17	0.44
		OGOLEM	37	1.50
Acetate Ion + H_2O	4	COMPLAX	113	1.03
		CREST	170	1.83
		SOLVATOR	64	0.72
		ABCluster	27	0.08
		OGOLEM	122	0.65

Notably, in the cases of complex coordination environments such as the organolithium and Pd(II) systems, tools like CREST, ABCluster, and OGOLEM generated structures that converged in fewer DFT cycles and with lower structural RMSD compared to COMPLAX. However, for the organolithium cluster, these structures relaxed into significantly higher-energy local minima (e.g., +4.6, +1.8 kcal mol^{-1}, and +10.2 kcal mol^{-1}, respectively). This fast convergence to suboptimal states demonstrates that unconstrained conformational search algorithms can easily become trapped in shallow configurational basins. While OGOLEM did reach the same energetic minimum as COMPLAX for the Pd(II) species in fewer cycles (37 vs 74), the targeted, rigid-core spatial sampling implemented in COMPLAX successfully identifies the correct, deeper energetic minima for highly

directional interactions, justifying the additional DFT optimization steps required to properly relax the system. Furthermore, although ABCluster located a deeper minimum for the highly flexible acetate ion, structural comparison of the optimized geometries reveals significant topological similarities (Figure S5). This demonstrates that COMPLAX successfully places the solvent molecules within the correct, chemically relevant configurational basin, achieving comparable structural soundness without relying on exhaustive conformational screening.

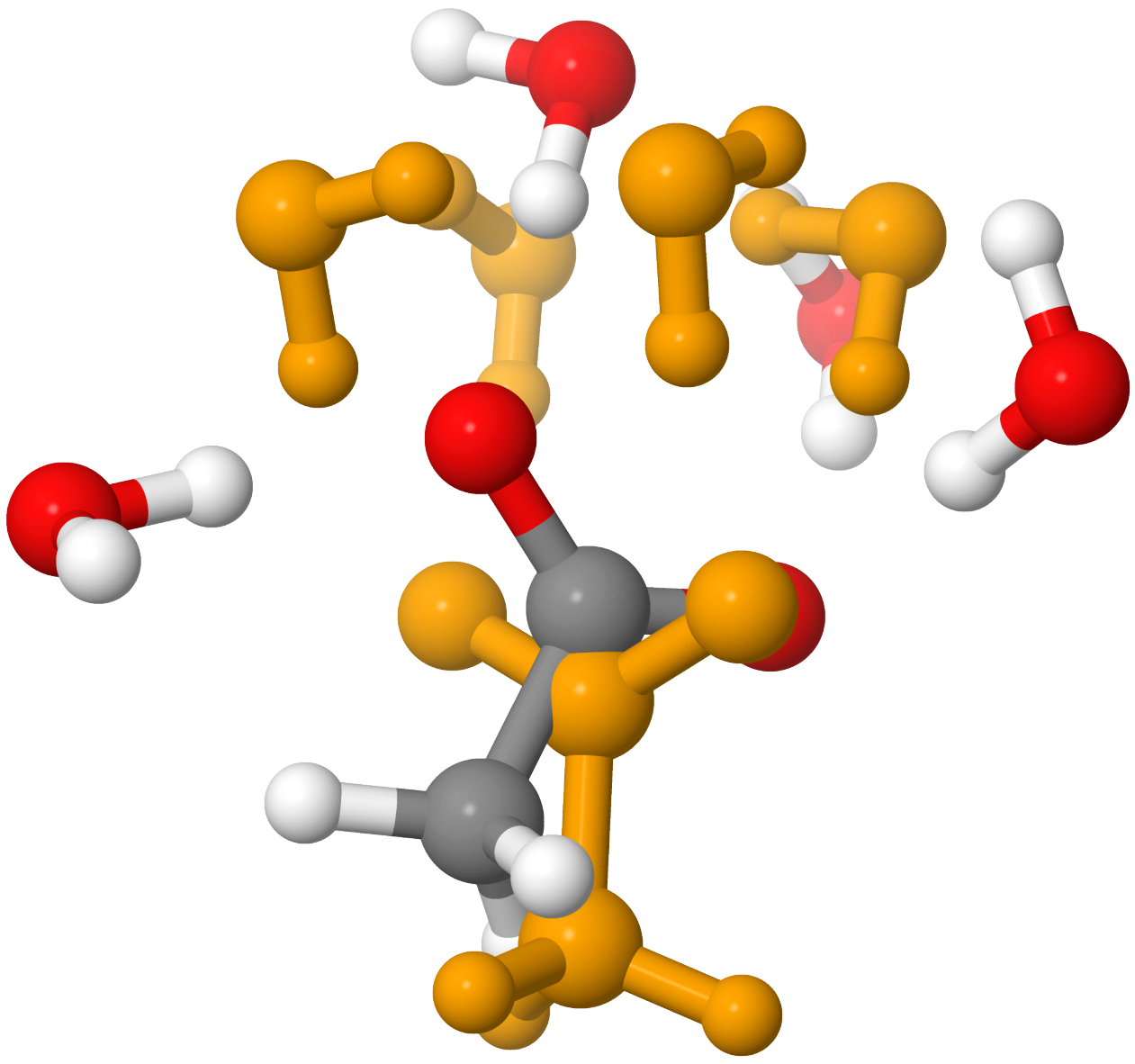

Figure S5: Structural comparison of the final DFT-optimized geometries originating from COMPLAX (standard CPK colors) and ABCluster (orange).

It should be noted that by simply increasing the sampling size of the initially generated configurations (via the `--nstruct` flag), COMPLAX successfully identifies a starting geometry that converges to an even deeper energetic minimum than that found by ABCluster, confirming its competitiveness when exhaustive configurational screening is desired.

S5.4 Reproducibility and Stochasticity in Cluster Generation

To evaluate the efficiency of the conformational sampling algorithm, we monitored the energy convergence across consecutive sampling steps. Figure S6 illustrates the relative energy (ΔE) of the most stable conformer identified up to step N, evaluated against the absolute global minimum of its respective solvation class. To highlight the impact of the initialization method, the test compares a reproducible deterministic generation (*--seed* 42, left panel) with a purely stochastic exploration (*--seed* -1, right panel).

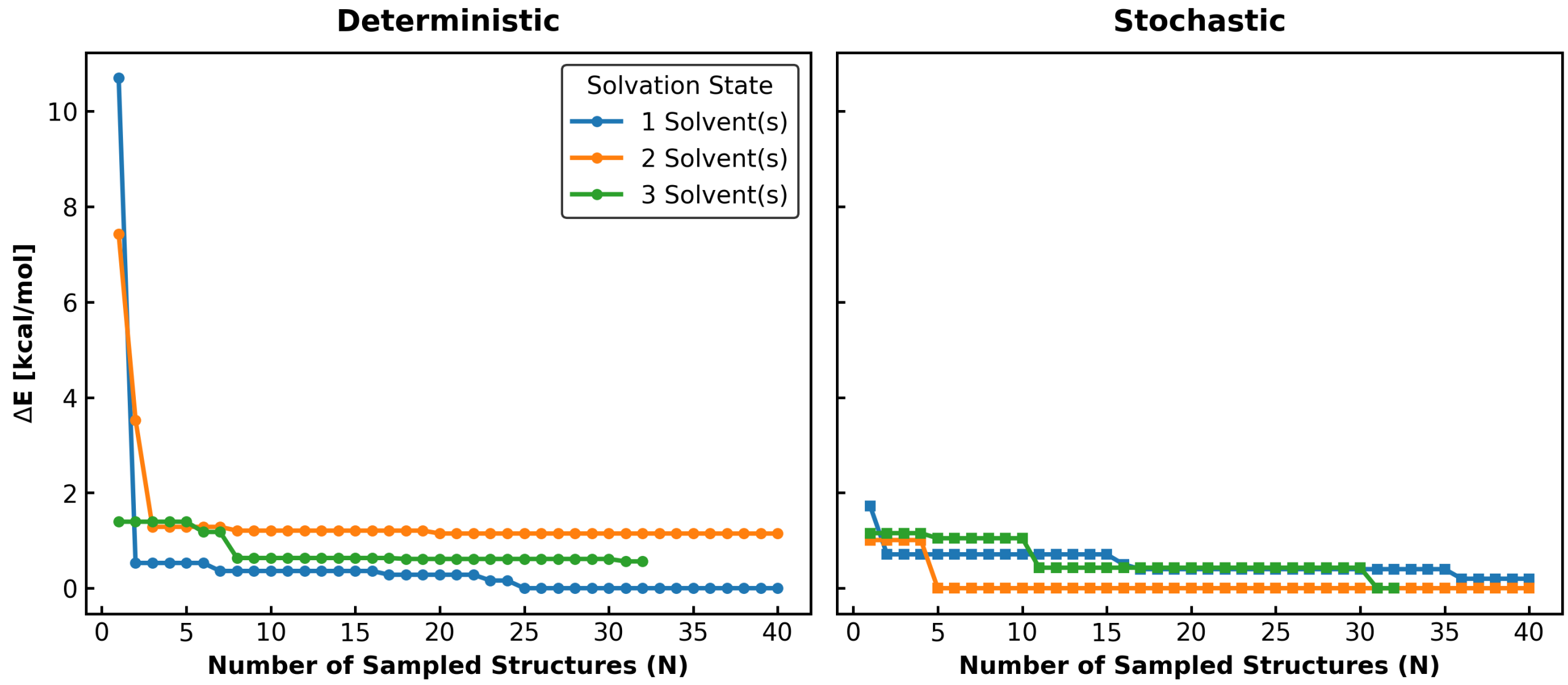

Figure S6: Conformational sampling convergence test for different targeted microsolvation states. Relative energy, ΔE expressed in kcal mol^{-1}, of the lowest-energy conformer found up to step N compared to the most stable conformer of that specific solvation class.

For minimal sampling size ($N < 10$), the energetic profiles exhibit structural bias and variations depending on the starting coordinates, highlighting the necessity of adequate sampling to overcome local minima traps. However, by progressively increasing the ensemble size, the energy curves for both sampling strategies systematically collapsed.

For all tested solvation states, expanding the ensemble size to $N = 40$ confined the energetic discrepancy well within the chemical accuracy threshold (< 1.0 kcal mol^{-1}). Therefore, this demonstrates that, given a sufficiently large generated ensemble, COMPLAX effectively eliminates the starting-geometry bias, ensuring an exhaustive exploration of the local potential energy surface and reliably converging to the correct global thermodynamic minimum regardless of the initial pseudo-random seed.

S6 Cartesian Coordinates

organolithium

Atom	X	Y	Z (Angstrom)
Li	0.994395	-0.210191	0.383519
O	-0.029627	0.202840	1.754377
C	0.976031	0.097645	2.667120
C	1.610175	1.505130	2.922888
Si	2.421633	2.201112	4.495625
C	2.951256	3.948292	4.002626
N	2.017886	-0.803677	2.045251
C	1.642210	-2.229210	1.917601
C	2.938558	-3.013362	2.136663
C	4.024262	-1.999715	1.775459
C	3.451793	-0.704023	2.339632
C	0.446482	-0.464666	4.010340
C	-0.896360	-0.246830	4.333931
C	-1.421156	-0.659453	5.558990
C	-0.610725	-1.319867	6.483283
C	0.724765	-1.567849	6.163621
C	1.245353	-1.141631	4.941023
C	3.958690	1.327429	5.158124
C	1.160493	2.350084	5.890216
H	-1.521059	0.253318	3.591407
H	2.293388	-1.350380	4.718008
H	-2.472789	-0.468276	5.792143
H	1.369535	-2.096824	6.871250
H	-1.018960	-1.647699	7.443292
H	0.743326	2.182695	2.820430
H	2.253784	1.756418	2.059736
H	1.229272	-2.449496	0.911503
H	0.856086	-2.508216	2.636629
H	3.894920	0.190914	1.876696
H	3.663278	-0.646970	3.420944
H	3.031104	-3.292687	3.199022
H	2.980955	-3.939230	1.544921
H	5.011226	-2.243350	2.195105
H	4.131680	-1.923785	0.680143
H	0.853824	1.364779	6.274222
H	1.585562	2.927243	6.727854
H	0.254685	2.873327	5.543165
H	2.095296	4.533385	3.628583
H	3.379162	4.488487	4.862953
H	3.714772	3.922801	3.207738
H	3.743767	0.315256	5.532748
H	4.747963	1.254695	4.393492
H	4.361573	1.914302	6.000274

Energy -1012.347336 (Hartree)

THF

Atom	X	Y	Z (Angstrom)
C	1.191647	-0.420882	-0.306247
O	0.014341	-1.214929	-0.352470
C	-1.123840	-0.439637	-0.003758
C	-0.665478	1.015940	-0.025019
C	0.798424	0.878675	0.391168
H	1.982411	-0.974168	0.225805
H	1.550674	-0.225789	-1.334627
H	-1.936936	-0.647466	-0.717937
H	-1.478510	-0.728242	1.004043
H	-0.736758	1.426857	-1.045178

H	-1.257439	1.658156	0.642608
H	1.426071	1.729497	0.089972
H	0.876736	0.767584	1.484856

Energy -232.225015 (Hartree)

Li-Cluster-1.1-COMPLAX-Best

Atom	X	Y	Z (Angstrom)
Li	0.992401	-0.265918	0.402254
O	-0.046658	0.167552	1.733473
C	0.962800	0.089183	2.645681
C	1.582525	1.500735	2.892829
Si	2.396958	2.162122	4.479471
C	2.938467	3.915305	4.021139
N	2.021405	-0.796974	2.031175
C	1.665422	-2.230506	1.910582
C	2.970157	-2.991157	2.165062
C	4.043242	-1.967746	1.789681
C	3.451228	-0.674583	2.342310
C	0.448222	-0.458501	3.995133
C	-0.872003	-0.171570	4.357936
C	-1.376636	-0.555188	5.601173
C	-0.569153	-1.251842	6.502206
C	0.741728	-1.568574	6.141591
C	1.243784	-1.173594	4.899719
C	3.924908	1.274167	5.146616
C	1.128690	2.293441	5.870232
H	-1.494307	0.357628	3.635613
H	2.268817	-1.440025	4.638243
H	-2.407782	-0.313326	5.867550
H	1.378393	-2.128930	6.829327
H	-0.961475	-1.555780	7.474416
H	0.709635	2.172923	2.810394
H	2.217644	1.758239	2.026098
H	1.282657	-2.465555	0.896625
H	0.867439	-2.508297	2.614741
H	3.883968	0.223695	1.878511
H	3.646566	-0.610753	3.424744
H	3.054293	-3.235408	3.235542
H	3.032916	-3.929291	1.598183
H	5.032509	-2.193865	2.209216
H	4.140108	-1.900169	0.694278
H	0.827919	1.304284	6.247672
H	1.552771	2.871249	6.706824
H	0.223100	2.813456	5.520272
H	2.086258	4.504850	3.648899
H	3.358620	4.436817	4.895145
H	3.708082	3.896686	3.233635
H	3.699797	0.260986	5.509829
H	4.722055	1.208492	4.391273
H	4.310998	1.858507	5.997254
C	2.460701	-0.695148	-2.108546
O	1.642024	0.089000	-1.264628
C	1.970714	1.432078	-1.552333
C	3.501936	1.449677	-1.681092
C	3.829747	0.008925	-2.136610
H	2.486942	-1.708655	-1.701416
H	2.023761	-0.719514	-3.118668
H	1.499158	1.733839	-2.498416
H	1.581384	2.047739	-0.740198
H	3.846574	2.189852	-2.402491
H	3.954947	1.657009	-0.711715
H	4.257113	-0.014476	-3.137610
H	4.522626	-0.464514	-1.442714

Energy -70.127941 (Hartree)

Li-Cluster-1.1-COMPLAX-DFT

Atom	X	Y	Z (Angstrom)
Li	1.166660	-0.966539	0.242001
O	0.171883	-0.219794	1.496686
C	1.123656	-0.278869	2.453151
C	1.914498	1.073074	2.501764
Si	2.747445	1.950060	3.964827
C	3.486209	3.510887	3.185422
N	2.081493	-1.387126	2.048060
C	1.546487	-2.758802	2.152470
C	2.733460	-3.626787	2.579001
C	3.942446	-2.822263	2.101495
C	3.503727	-1.389768	2.389177
C	0.497666	-0.545368	3.844477
C	-0.831934	-0.161505	4.036821
C	-1.444974	-0.297753	5.281159
C	-0.742572	-0.843073	6.355959
C	0.575694	-1.257235	6.170094
C	1.188050	-1.105383	4.925870
C	4.166656	1.060031	4.839742
C	1.474785	2.477717	5.247957
H	-1.361431	0.236554	3.168811
H	2.221599	-1.437007	4.806280
H	-2.483591	0.018630	5.412755
H	1.134196	-1.700400	6.999250
H	-1.222657	-0.953896	7.331834
H	1.130029	1.807162	2.242903
H	2.604714	1.094816	1.639761
H	1.142692	-3.112597	1.180254
H	0.706094	-2.812497	2.861694
H	4.063653	-0.650078	1.793736
H	3.696083	-1.149241	3.449469
H	2.755862	-3.712813	3.677680
H	2.689485	-4.647345	2.171457
H	4.881224	-3.090751	2.607937
H	4.096159	-2.964670	1.017742
H	1.052121	1.615991	5.786797
H	1.931879	3.154023	5.988555
H	0.638164	3.013378	4.772007
H	2.709370	4.111278	2.684401
H	3.963435	4.152244	3.943803
H	4.252502	3.259534	2.433310
H	3.831238	0.165590	5.385506
H	4.964999	0.763262	4.141664
H	4.611763	1.746124	5.578901
C	3.147565	0.305486	-1.710557
O	1.841044	0.127065	-1.189875
C	1.192555	1.407388	-1.147905
C	2.319344	2.448694	-1.013047
C	3.617688	1.626006	-1.110019
H	3.755864	-0.563472	-1.420356
H	3.108456	0.351319	-2.815295
H	0.617914	1.546332	-2.079431
H	0.504624	1.374932	-0.288818
H	2.257143	3.197613	-1.815689
H	2.258094	2.986014	-0.056533
H	4.392677	2.111602	-1.719417
H	4.042212	1.450803	-0.109585

Energy -1244.588716 (Hartree)

Li-Cluster-1.2-COMPLAX-Best

Atom	X	Y	Z (Angstrom)
Li	0.992401	-0.265918	0.402254
O	-0.046658	0.167552	1.733473
C	0.962800	0.089183	2.645681
C	1.582525	1.500735	2.892829
Si	2.396958	2.162122	4.479471
C	2.938467	3.915305	4.021139
N	2.021405	-0.796974	2.031175
C	1.665422	-2.230506	1.910582
C	2.970157	-2.991157	2.165062
C	4.043242	-1.967746	1.789681
C	3.451228	-0.674583	2.342310
C	0.448222	-0.458501	3.995133
C	-0.872003	-0.171570	4.357936
C	-1.376636	-0.555188	5.601172
C	-0.569153	-1.251842	6.502206
C	0.741728	-1.568574	6.141591
C	1.243784	-1.173594	4.899719
C	3.924908	1.274167	5.146616
C	1.128690	2.293441	5.870232
H	-1.494307	0.357628	3.635613
H	2.268817	-1.440025	4.638243
H	-2.407782	-0.313326	5.867550
H	1.378393	-2.128930	6.829327
H	-0.961475	-1.555780	7.474416
H	0.709635	2.172923	2.810394
H	2.217644	1.758239	2.026098
H	1.282657	-2.465555	0.896625
H	0.867439	-2.508297	2.614741
H	3.883968	0.223695	1.878511
H	3.646566	-0.610753	3.424744
H	3.054293	-3.235408	3.235542
H	3.032916	-3.929291	1.598183
H	5.032509	-2.193865	2.209216
H	4.140108	-1.900169	0.694278
H	0.827919	1.304284	6.247672
H	1.552771	2.871249	6.706824
H	0.223100	2.813456	5.520272
H	2.086258	4.504850	3.648899
H	3.358620	4.436817	4.895145
H	3.708082	3.896686	3.233635
H	3.699797	0.260986	5.509829
H	4.722055	1.208492	4.391273
H	4.310998	1.858507	5.997254
C	1.501295	-1.327038	-2.391671
O	0.701075	-0.830976	-1.338995
C	-0.571893	-0.596128	-1.904257
C	-0.293247	0.038232	-3.275556
C	1.102691	-0.517421	-3.640664
H	2.547851	-1.211932	-2.100675
H	1.282446	-2.394562	-2.546892
H	-1.103579	-1.551259	-2.024737
H	-1.122510	0.043607	-1.213750
H	-1.045975	-0.241662	-4.011223
H	-0.266518	1.124766	-3.188728
H	1.071512	-1.156327	-4.522034
H	1.807396	0.294628	-3.814430

Energy -70.127685 (Hartree)

Li-Cluster-1.2-COMPLAX-DFT

Atom	X	Y	Z (Angstrom)
Li	1.871987	0.027257	0.104065
O	0.666781	0.496331	1.304867

C	1.426939	0.261420	2.397858
C	2.091360	1.595247	2.879789
Si	2.523009	2.157021	4.642433
C	3.280606	3.875693	4.409765
N	2.517903	-0.703490	1.967223
C	2.067544	-2.084526	1.688647
C	3.187970	-2.992847	2.201253
C	4.416379	-2.084496	2.155589
C	3.838578	-0.744438	2.599806
C	0.563870	-0.319952	3.546295
C	-0.799890	-0.013363	3.546374
C	-1.632665	-0.438972	4.579740
C	-1.118910	-1.201264	5.628895
C	0.234200	-1.536573	5.628532
C	1.065135	-1.095656	4.598247
C	3.791156	1.135592	5.603801
C	0.971542	2.340098	5.695359
H	-1.176900	0.567169	2.702029
H	2.121065	-1.371696	4.623180
H	-2.694420	-0.176872	4.565063
H	0.649446	-2.144385	6.437191
H	-1.770540	-1.538764	6.439165
H	1.331641	2.345066	2.595825
H	2.947796	1.818349	2.217013
H	1.915834	-2.244922	0.602980
H	1.100128	-2.296725	2.169867
H	4.463207	0.107460	2.287802
H	3.788530	-0.710087	3.701919
H	2.983656	-3.290974	3.242633
H	3.295870	-3.915026	1.611550
H	5.243204	-2.424568	2.796418
H	4.801868	-2.011212	1.124230
H	0.522999	1.366067	5.943341
H	1.208010	2.857920	6.639229
H	0.207372	2.932308	5.167354
H	2.589883	4.547044	3.874736
H	3.514159	4.341334	5.380885
H	4.216239	3.827699	3.829094
H	3.419765	0.132521	5.861267
H	4.738871	1.025817	5.053695
H	4.016679	1.653228	6.550651
C	1.249771	-0.499759	-2.894241
O	1.131042	-0.768370	-1.495871
C	-0.214299	-1.170909	-1.169349
C	-1.058549	-0.557888	-2.272140
C	-0.144506	-0.718688	-3.488692
H	1.591142	0.541581	-3.022062
H	2.010191	-1.165496	-3.333020
H	-0.272574	-2.274599	-1.182256
H	-0.417956	-0.797164	-0.153258
H	-2.030467	-1.056988	-2.389885
H	-1.244914	0.505924	-2.055833
H	-0.231935	-1.737381	-3.898070
H	-0.365793	-0.013403	-4.302038

Energy -1244.586630 (Hartree)

Li-Cluster-1.3-COMPLAX-Best

Atom	X	Y	Z (Angstrom)
Li	0.992401	-0.265918	0.402254
O	-0.046658	0.167552	1.733473
C	0.962800	0.089183	2.645681
C	1.582525	1.500735	2.892829
Si	2.396958	2.162122	4.479471

C	2.938467	3.915305	4.021139
N	2.021405	-0.796974	2.031175
C	1.665422	-2.230506	1.910582
C	2.970157	-2.991157	2.165062
C	4.043242	-1.967746	1.789681
C	3.451228	-0.674583	2.342310
C	0.448222	-0.458501	3.995133
C	-0.872003	-0.171570	4.357936
C	-1.376636	-0.555188	5.601172
C	-0.569153	-1.251842	6.502206
C	0.741728	-1.568574	6.141591
C	1.243784	-1.173594	4.899719
C	3.924908	1.274167	5.146616
C	1.128690	2.293441	5.870232
H	-1.494307	0.357628	3.635613
H	2.268817	-1.440025	4.638243
H	-2.407782	-0.313326	5.867550
H	1.378393	-2.128930	6.829327
H	-0.961475	-1.555780	7.474416
H	0.709635	2.172923	2.810394
H	2.217644	1.758239	2.026098
H	1.282657	-2.465555	0.896625
H	0.867439	-2.508297	2.614741
H	3.883968	0.223695	1.878511
H	3.646566	-0.610753	3.424744
H	3.054293	-3.235408	3.235542
H	3.032916	-3.929291	1.598183
H	5.032509	-2.193865	2.209216
H	4.140108	-1.900169	0.694278
H	0.827919	1.304284	6.247672
H	1.552771	2.871249	6.706824
H	0.223100	2.813456	5.520272
H	2.086258	4.504850	3.648899
H	3.358620	4.436817	4.895145
H	3.708082	3.896686	3.233635
H	3.699797	0.260986	5.509829
H	4.722055	1.208492	4.391273
H	4.310998	1.858507	5.997254
C	2.438879	-0.702314	-2.154978
O	1.336778	-0.912474	-1.296795
C	0.419232	-1.690046	-2.037766
C	0.439654	-1.104194	-3.458866
C	1.840393	-0.455873	-3.552413
H	3.015854	0.137233	-1.760788
H	3.069253	-1.604068	-2.169101
H	0.750461	-2.738778	-2.051467
H	-0.551650	-1.620643	-1.545109
H	0.298722	-1.875678	-4.214276
H	-0.346353	-0.355081	-3.564736
H	2.460626	-0.912612	-4.323403
H	1.755902	0.611676	-3.750757

Energy -70.127587 (Hartree)

Li-Cluster-1.3-COMPLAX-DFT

Atom	X	Y	Z (Angstrom)
Li	1.859490	0.563339	0.082697
O	0.609387	0.638675	1.326480
C	1.424850	0.397317	2.376188
C	1.901258	1.750610	3.004611
Si	2.309142	2.160467	4.814097
C	2.800002	3.987978	4.776830
N	2.633753	-0.338635	1.822237
C	2.376393	-1.727604	1.388747

C	3.655605	-2.503939	1.715081
C	4.723695	-1.411501	1.769648
C	3.966003	-0.261398	2.424643
C	0.690829	-0.434234	3.458227
C	-0.702750	-0.339429	3.506643
C	-1.434165	-1.000116	4.492008
C	-0.784260	-1.790255	5.440289
C	0.603094	-1.916161	5.387126
C	1.330643	-1.240717	4.406908
C	3.746393	1.232940	5.620137
C	0.785216	1.988514	5.908372
H	-1.183920	0.268266	2.737694
H	2.416149	-1.356116	4.388178
H	-2.523117	-0.901537	4.518577
H	1.125925	-2.542580	6.115173
H	-1.356078	-2.311674	6.212453
H	1.028955	2.404786	2.827868
H	2.690258	2.177323	2.357658
H	2.161237	-1.769493	0.304401
H	1.490965	-2.149873	1.889065
H	4.437378	0.716221	2.238307
H	3.956096	-0.402174	3.519852
H	3.563098	-2.981008	2.704440
H	3.872205	-3.300508	0.987790
H	5.625909	-1.701836	2.327849
H	5.038810	-1.128253	0.750406
H	0.487289	0.937820	6.044627
H	0.979432	2.420836	6.903572
H	-0.072694	2.522622	5.469954
H	2.000270	4.605840	4.337780
H	2.996709	4.368032	5.792340
H	3.711584	4.147873	4.178215
H	3.533491	0.163265	5.764083
H	4.677714	1.328109	5.040090
H	3.932254	1.665767	6.616826
C	1.939352	-1.072080	-2.529707
O	1.326216	-0.172144	-1.617221
C	-0.094098	-0.432131	-1.539161
C	-0.327596	-1.732627	-2.317950
C	1.077103	-2.324508	-2.467680
H	1.946235	-0.635268	-3.545861
H	2.982227	-1.228498	-2.214547
H	-0.341473	-0.487596	-0.466750
H	-0.633652	0.422071	-1.976981
H	-1.018524	-2.407133	-1.793973
H	-0.758878	-1.521728	-3.308772
H	1.346384	-2.922387	-1.582583
H	1.188427	-2.963618	-3.354887

Energy -1244.586827 (Hartree)

Li-Cluster-2.1-COMPLAX-Best

Atom	X	Y	Z (Angstrom)
Li	0.992401	-0.265918	0.402254
O	-0.046658	0.167552	1.733473
C	0.962800	0.089183	2.645681
C	1.582525	1.500735	2.892829
Si	2.396958	2.162122	4.479471
C	2.938467	3.915305	4.021139
N	2.021405	-0.796974	2.031175
C	1.665422	-2.230506	1.910582
C	2.970157	-2.991157	2.165062
C	4.043242	-1.967746	1.789681
C	3.451228	-0.674583	2.342310

```
  C     0.448222    -0.458501     3.995133
  C    -0.872003    -0.171570     4.357936
  C    -1.376636    -0.555188     5.601173
  C    -0.569153    -1.251842     6.502206
  C     0.741728    -1.568574     6.141591
  C     1.243784    -1.173594     4.899719
  C     3.924908     1.274167     5.146616
  C     1.128690     2.293441     5.870232
  H    -1.494307     0.357628     3.635613
  H     2.268817    -1.440025     4.638243
  H    -2.407782    -0.313326     5.867550
  H     1.378393    -2.128930     6.829327
  H    -0.961475    -1.555780     7.474416
  H     0.709635     2.172923     2.810394
  H     2.217644     1.758239     2.026098
  H     1.282657    -2.465555     0.896625
  H     0.867439    -2.508297     2.614741
  H     3.883968     0.223695     1.878511
  H     3.646566    -0.610753     3.424744
  H     3.054293    -3.235408     3.235542
  H     3.032916    -3.929291     1.598183
  H     5.032509    -2.193865     2.209216
  H     4.140108    -1.900169     0.694278
  H     0.827919     1.304284     6.247672
  H     1.552771     2.871249     6.706824
  H     0.223100     2.813456     5.520272
  H     2.086258     4.504850     3.648899
  H     3.358620     4.436817     4.895145
  H     3.708082     3.896686     3.233635
  H     3.699797     0.260986     5.509829
  H     4.722055     1.208492     4.391273
  H     4.310998     1.858507     5.997254
  C     0.340448    -1.144719    -2.498246
  O     0.290536    -1.271431    -1.092371
  C    -1.031507    -1.671942    -0.794818
  C    -1.928082    -0.838904    -1.724302
  C    -0.995757    -0.501766    -2.909236
  H     1.220350    -0.549382    -2.747295
  H     0.434213    -2.144587    -2.951098
  H    -1.146960    -2.746251    -0.997352
  H    -1.198730    -1.474835     0.260805
  H    -2.808995    -1.392932    -2.044469
  H    -2.245018     0.064439    -1.203864
  H    -1.353373    -0.914028    -3.852509
  H    -0.884291     0.575960    -3.011447
  C     0.597916     2.470567    -0.337846
  O     1.504414     1.412548    -0.575582
  C     2.460222     1.933171    -1.475338
  C     1.662491     2.773414    -2.485888
  C     0.396989     3.168387    -1.690827
  H    -0.303417     2.029689     0.085118
  H     1.036283     3.174500     0.385935
  H     3.175763     2.571010    -0.933044
  H     2.991514     1.092771    -1.924059
  H     2.223645     3.647667    -2.812678
  H     1.405382     2.171101    -3.358963
  H     0.311620     4.247528    -1.554839
  H    -0.503065     2.802991    -2.184743
Energy          -86.859686  (Hartree)
```

Li-Cluster-2.1-COMPLAX-DFT

```
 Atom       X             Y             Z (Angstrom)
 Li     1.317980    -0.105830     0.230314
```

O	0.172864	0.224785	1.612102
C	1.098523	0.092953	2.591127
C	1.729985	1.489357	2.923619
Si	2.415847	2.149125	4.566355
C	3.014414	3.894930	4.139698
N	2.178873	-0.815656	2.053190
C	1.802918	-2.235017	1.891117
C	3.075324	-3.029236	2.202467
C	4.190450	-2.019378	1.932001
C	3.579710	-0.725957	2.460992
C	0.452343	-0.473825	3.880708
C	-0.909791	-0.234302	4.081653
C	-1.547604	-0.645075	5.250850
C	-0.835510	-1.325777	6.238572
C	0.517806	-1.595275	6.039752
C	1.153238	-1.169028	4.873114
C	3.884520	1.253261	5.349291
C	1.050232	2.303567	5.855503
H	-1.444831	0.280320	3.280953
H	2.213890	-1.392610	4.740889
H	-2.612074	-0.436703	5.391895
H	1.086241	-2.140365	6.798492
H	-1.333986	-1.651875	7.155295
H	0.875447	2.174800	2.779133
H	2.432252	1.753640	2.112771
H	1.460828	-2.440805	0.860345
H	0.968176	-2.510201	2.555386
H	4.064889	0.170720	2.043331
H	3.713915	-0.677636	3.556630
H	3.089551	-3.320106	3.265920
H	3.160015	-3.952063	1.609746
H	5.144579	-2.271058	2.418507
H	4.378257	-1.935234	0.847861
H	0.700693	1.321017	6.207642
H	1.410057	2.871753	6.728875
H	0.179090	2.836297	5.442053
H	2.202611	4.497679	3.701073
H	3.375925	4.424618	5.035916
H	3.841453	3.868286	3.411359
H	3.624942	0.243291	5.699889
H	4.738592	1.173066	4.658921
H	4.220755	1.828571	6.227572
C	0.229796	-1.129059	-2.484371
O	0.335787	-1.242875	-1.076375
C	-0.967536	-1.529108	-0.561775
C	-1.879364	-0.634028	-1.391667
C	-1.194701	-0.609735	-2.771652
H	1.024094	-0.451617	-2.830809
H	0.397823	-2.116629	-2.950547
H	-1.194265	-2.601626	-0.713164
H	-0.944966	-1.275862	0.509158
H	-2.913592	-1.003565	-1.431770
H	-1.899287	0.370186	-0.941643
H	-1.707522	-1.262975	-3.492660
H	-1.182289	0.400994	-3.205143
C	0.868140	2.596170	-0.330771
O	1.806728	1.580005	-0.684808
C	2.450080	2.016767	-1.865703
C	1.354765	2.703952	-2.699849
C	0.248381	3.013329	-1.665964
H	0.183627	2.148818	0.405593
H	1.410146	3.436802	0.142074
H	3.255125	2.728450	-1.604756

H	2.910726	1.145507	-2.353476
H	1.739116	3.608732	-3.191854
H	0.981853	2.038925	-3.492784
H	-0.051894	4.070417	-1.665692
H	-0.653099	2.416732	-1.868874

Energy -1476.839956 (Hartree)

Li-Cluster-2.2-COMPLAX-Best

Atom	X	Y	Z (Angstrom)
Li	0.992401	-0.265918	0.402254
O	-0.046658	0.167552	1.733473
C	0.962800	0.089183	2.645681
C	1.582525	1.500735	2.892829
Si	2.396958	2.162122	4.479471
C	2.938467	3.915305	4.021139
N	2.021405	-0.796974	2.031175
C	1.665422	-2.230506	1.910582
C	2.970157	-2.991157	2.165062
C	4.043242	-1.967746	1.789681
C	3.451228	-0.674583	2.342310
C	0.448222	-0.458501	3.995133
C	-0.872003	-0.171570	4.357936
C	-1.376636	-0.555188	5.601172
C	-0.569153	-1.251842	6.502206
C	0.741728	-1.568574	6.141591
C	1.243784	-1.173594	4.899719
C	3.924908	1.274167	5.146616
C	1.128690	2.293441	5.870232
H	-1.494307	0.357628	3.635613
H	2.268817	-1.440025	4.638243
H	-2.407782	-0.313326	5.867550
H	1.378393	-2.128930	6.829327
H	-0.961475	-1.555780	7.474416
H	0.709635	2.172923	2.810394
H	2.217644	1.758239	2.026098
H	1.282657	-2.465555	0.896625
H	0.867439	-2.508297	2.614741
H	3.883968	0.223695	1.878511
H	3.646566	-0.610753	3.424744
H	3.054293	-3.235408	3.235542
H	3.032916	-3.929291	1.598183
H	5.032509	-2.193865	2.209216
H	4.140108	-1.900169	0.694278
H	0.827919	1.304284	6.247672
H	1.552771	2.871249	6.706824
H	0.223100	2.813456	5.520272
H	2.086258	4.504850	3.648899
H	3.358620	4.436817	4.895145
H	3.708082	3.896686	3.233635
H	3.699797	0.260986	5.509829
H	4.722055	1.208492	4.391273
H	4.310998	1.858507	5.997254
C	-0.850804	-1.074076	-1.700812
O	0.477751	-1.144672	-1.227644
C	1.196427	-1.859231	-2.211273
C	0.266534	-3.001659	-2.648143
C	-1.141751	-2.442686	-2.341158
H	-1.484699	-0.830158	-0.848956
H	-0.924666	-0.277747	-2.456104
H	1.413446	-1.196638	-3.061515
H	2.130682	-2.195879	-1.761705
H	0.386522	-3.241508	-3.703748
H	0.467612	-3.895615	-2.056205

H	-1.745619	-2.326518	-3.241235
H	-1.669118	-3.089659	-1.641663
C	3.195869	1.497502	-1.038840
O	1.903758	1.198634	-0.555008
C	1.163963	2.394215	-0.685742
C	2.121314	3.525918	-0.280635
C	3.519040	2.915135	-0.533223
H	3.876677	0.726511	-0.675851
H	3.183143	1.486301	-2.139004
H	0.847661	2.514808	-1.731604
H	0.291064	2.307033	-0.043105
H	1.949425	4.419931	-0.876841
H	1.980692	3.770359	0.774039
H	4.095689	3.468755	-1.275838
H	4.089551	2.870959	0.394430

Energy -86.859685 (Hartree)

Li-Cluster-2.2-COMPLAX-DFT

Atom	X	Y	Z (Angstrom)
Li	1.332335	0.037918	-0.054121
O	0.215665	0.367454	1.342575
C	1.106374	0.157531	2.335356
C	1.750490	1.520538	2.767886
Si	2.395288	2.084811	4.460272
C	3.022123	3.844764	4.140726
N	2.189711	-0.743119	1.784693
C	1.815862	-2.151802	1.561450
C	3.091656	-2.958208	1.824718
C	4.203562	-1.931757	1.608868
C	3.586057	-0.671414	2.205165
C	0.418080	-0.469717	3.574224
C	-0.947406	-0.231671	3.750158
C	-1.618612	-0.699619	4.878817
C	-0.936911	-1.435425	5.848229
C	0.420569	-1.701930	5.673030
C	1.089020	-1.219264	4.547836
C	3.838261	1.133668	5.225771
C	0.999427	2.187123	5.720693
H	-1.456979	0.330374	2.964673
H	2.152500	-1.440276	4.431806
H	-2.685404	-0.492028	5.002284
H	0.965417	-2.288404	6.418111
H	-1.461860	-1.805979	6.732712
H	0.912582	2.226277	2.623026
H	2.486071	1.806090	1.997205
H	1.463838	-2.307267	0.526038
H	0.986019	-2.462831	2.216516
H	4.072104	0.248157	1.840047
H	3.716400	-0.681785	3.303041
H	3.109715	-3.307492	2.870271
H	3.177953	-3.847199	1.182162
H	5.158259	-2.206131	2.081733
H	4.391637	-1.788659	0.530653
H	0.632579	1.191110	6.012130
H	1.340369	2.706331	6.631287
H	0.144104	2.748302	5.311985
H	2.221229	4.485779	3.737288
H	3.386714	4.315045	5.068262
H	3.853916	3.851341	3.416737
H	3.563622	0.106783	5.509339
H	4.708547	1.087284	4.552574
H	4.157130	1.653001	6.144434
C	-0.991547	-1.324124	-0.910684

O	0.343061	-1.077073	-1.358181
C	0.622159	-2.019832	-2.373328
C	-0.038335	-3.325040	-1.905848
C	-1.125898	-2.851359	-0.914624
H	-1.078653	-0.850491	0.079119
H	-1.698177	-0.855494	-1.620731
H	0.189771	-1.673682	-3.331211
H	1.713129	-2.086542	-2.493889
H	-0.449805	-3.892874	-2.752625
H	0.694616	-3.973716	-1.404139
H	-2.135562	-3.167036	-1.213205
H	-0.941018	-3.254035	0.091365
C	3.509243	2.043709	-1.125075
O	2.193024	1.619314	-0.833876
C	1.388116	2.777189	-0.603004
C	2.343525	3.854107	-0.054306
C	3.745184	3.228271	-0.191215
H	4.189435	1.195970	-0.957775
H	3.583607	2.346111	-2.187712
H	0.928264	3.092677	-1.556294
H	0.599575	2.469415	0.100819
H	2.257441	4.785498	-0.632414
H	2.117773	4.095854	0.993473
H	4.495062	3.929033	-0.584578
H	4.106629	2.869223	0.784444

Energy -1476.841698 (Hartree)

Li-Cluster-2.3-COMPLAX-Best

Atom	X	Y	Z (Angstrom)
Li	0.992401	-0.265918	0.402254
O	-0.046658	0.167552	1.733473
C	0.962800	0.089183	2.645681
C	1.582525	1.500735	2.892829
Si	2.396958	2.162122	4.479471
C	2.938467	3.915305	4.021139
N	2.021405	-0.796974	2.031175
C	1.665422	-2.230506	1.910582
C	2.970157	-2.991157	2.165062
C	4.043242	-1.967746	1.789681
C	3.451228	-0.674583	2.342310
C	0.448222	-0.458501	3.995133
C	-0.872003	-0.171570	4.357936
C	-1.376636	-0.555188	5.601172
C	-0.569153	-1.251842	6.502206
C	0.741728	-1.568574	6.141591
C	1.243784	-1.173594	4.899719
C	3.924908	1.274167	5.146616
C	1.128690	2.293441	5.870232
H	-1.494307	0.357628	3.635613
H	2.268817	-1.440025	4.638243
H	-2.407782	-0.313326	5.867550
H	1.378393	-2.128930	6.829327
H	-0.961475	-1.555780	7.474416
H	0.709635	2.172923	2.810394
H	2.217644	1.758239	2.026098
H	1.282657	-2.465555	0.896625
H	0.867439	-2.508297	2.614741
H	3.883968	0.223695	1.878511
H	3.646566	-0.610753	3.424744
H	3.054293	-3.235408	3.235542
H	3.032916	-3.929291	1.598183
H	5.032509	-2.193865	2.209216
H	4.140108	-1.900169	0.694278

H	0.827919	1.304284	6.247672
H	1.552771	2.871249	6.706824
H	0.223100	2.813456	5.520272
H	2.086258	4.504850	3.648899
H	3.358620	4.436817	4.895145
H	3.708082	3.896686	3.233635
H	3.699797	0.260986	5.509829
H	4.722055	1.208492	4.391273
H	4.310998	1.858507	5.997254
C	0.026034	-0.857832	-2.501934
O	0.006920	-1.031090	-1.103827
C	-1.353182	-1.216831	-0.763571
C	-2.124781	-0.198752	-1.617539
C	-1.209014	-0.003527	-2.844329
H	0.977621	-0.396588	-2.770076
H	-0.049546	-1.840926	-2.991942
H	-1.657712	-2.243264	-1.011735
H	-1.437500	-1.050333	0.306943
H	-3.111970	-0.560967	-1.902497
H	-2.233448	0.731894	-1.063644
H	-1.680941	-0.349288	-3.763098
H	-0.934442	1.044545	-2.957407
C	2.739085	2.000343	-1.145355
O	1.610988	1.399888	-0.549188
C	0.650823	2.431864	-0.441934
C	1.426308	3.690699	-0.024871
C	2.862024	3.399991	-0.515150
H	3.601299	1.355684	-0.967387
H	2.569865	2.096622	-2.229427
H	0.170231	2.586158	-1.417420
H	-0.084811	2.110093	0.289256
H	1.004744	4.581821	-0.485613
H	1.392973	3.804479	1.060219
H	3.215197	4.124779	-1.250800
H	3.555857	3.384296	0.325210

Energy -86.859588 (Hartree)

Li-Cluster-2.3-COMPLAX-DFT

Atom	X	Y	Z (Angstrom)
Li	1.100671	-0.146668	0.136251
O	0.037351	0.255935	1.552381
C	0.986364	0.147617	2.506858
C	1.567723	1.563364	2.844080
Si	2.255172	2.245621	4.475518
C	2.762803	4.019050	4.040705
N	2.092943	-0.713464	1.937884
C	1.782700	-2.147505	1.777065
C	3.107091	-2.878237	2.020691
C	4.156457	-1.808174	1.720088
C	3.500961	-0.555355	2.291223
C	0.398012	-0.465132	3.803347
C	-0.972267	-0.313132	4.030224
C	-1.560996	-0.771394	5.207782
C	-0.789181	-1.412508	6.176819
C	0.574704	-1.595235	5.951462
C	1.159834	-1.121911	4.776958
C	3.785384	1.415020	5.210316
C	0.914162	2.330332	5.795219
H	-1.552481	0.173050	3.243290
H	2.229817	-1.277420	4.622319
H	-2.633625	-0.631661	5.369939
H	1.189812	-2.108978	6.695510
H	-1.249000	-1.775849	7.099686

H	0.685460	2.213956	2.704407
H	2.253398	1.850505	2.029110
H	1.403258	-2.366093	0.760275
H	0.995246	-2.473799	2.475303
H	3.923085	0.370475	1.866919
H	3.677812	-0.510624	3.381506
H	3.181313	-3.181704	3.077980
H	3.213431	-3.787180	1.410609
H	5.141270	-2.015908	2.164192
H	4.295256	-1.701468	0.630392
H	0.616411	1.330414	6.146080
H	1.266097	2.907759	6.665640
H	0.011611	2.825953	5.403622
H	1.909339	4.591303	3.641622
H	3.138009	4.557914	4.925693
H	3.561277	4.033935	3.280201
H	3.584756	0.390930	5.558415
H	4.622458	1.381317	4.495532
H	4.119291	1.998272	6.084182
C	-0.371439	-0.651430	-2.385914
O	-0.017613	-1.201553	-1.125648
C	-1.234334	-1.581094	-0.482332
C	-2.181845	-0.425810	-0.785015
C	-1.719566	0.065516	-2.172232
H	0.442879	0.017491	-2.697349
H	-0.463295	-1.465073	-3.128381
H	-1.586919	-2.540868	-0.905496
H	-1.025771	-1.679097	0.590583
H	-3.238037	-0.729543	-0.765234
H	-2.024449	0.344607	-0.016197
H	-2.432673	-0.198384	-2.967037
H	-1.602149	1.158363	-2.190610
C	3.047009	1.844412	-1.273444
O	1.755044	1.404830	-0.896950
C	0.947109	2.550569	-0.608920
C	1.921833	3.671595	-0.199946
C	3.317781	3.047089	-0.375654
H	3.748578	1.009555	-1.132632
H	3.052579	2.128713	-2.343497
H	0.375016	2.824214	-1.513304
H	0.253096	2.242644	0.188812
H	1.794319	4.554117	-0.843594
H	1.754529	3.993823	0.837142
H	4.049799	3.741519	-0.811371
H	3.716423	2.706991	0.592357

Energy -1476.843230 (Hartree)

Li-Cluster-3.1-COMPLAX-Best

Atom	X	Y	Z (Angstrom)
Li	0.992401	-0.265918	0.402254
O	-0.046658	0.167552	1.733473
C	0.962800	0.089183	2.645681
C	1.582525	1.500735	2.892829
Si	2.396958	2.162122	4.479471
C	2.938467	3.915305	4.021139
N	2.021405	-0.796974	2.031175
C	1.665422	-2.230506	1.910582
C	2.970157	-2.991157	2.165062
C	4.043242	-1.967746	1.789681
C	3.451228	-0.674583	2.342310
C	0.448222	-0.458501	3.995133
C	-0.872003	-0.171570	4.357936
C	-1.376636	-0.555188	5.601172

C	-0.569153	-1.251842	6.502206
C	0.741728	-1.568574	6.141591
C	1.243784	-1.173594	4.899719
C	3.924908	1.274167	5.146616
C	1.128690	2.293441	5.870232
H	-1.494307	0.357628	3.635613
H	2.268817	-1.440025	4.638243
H	-2.407782	-0.313326	5.867550
H	1.378393	-2.128930	6.829327
H	-0.961475	-1.555780	7.474416
H	0.709635	2.172923	2.810394
H	2.217644	1.758239	2.026098
H	1.282657	-2.465555	0.896625
H	0.867439	-2.508297	2.614741
H	3.883968	0.223695	1.878511
H	3.646566	-0.610753	3.424744
H	3.054293	-3.235408	3.235542
H	3.032916	-3.929291	1.598183
H	5.032509	-2.193865	2.209216
H	4.140108	-1.900169	0.694278
H	0.827919	1.304284	6.247672
H	1.552771	2.871249	6.706824
H	0.223100	2.813456	5.520272
H	2.086258	4.504850	3.648899
H	3.358620	4.436817	4.895145
H	3.708082	3.896686	3.233635
H	3.699797	0.260986	5.509829
H	4.722055	1.208492	4.391273
H	4.310998	1.858507	5.997254
C	1.155683	2.637669	-0.932600
O	0.735941	1.288331	-0.951464
C	-0.631499	1.323383	-1.304488
C	-1.219258	2.492638	-0.500458
C	-0.010846	3.441268	-0.329432
H	2.082739	2.693290	-0.356316
H	1.352362	2.970908	-1.965758
H	-0.729566	1.499376	-2.384776
H	-1.046460	0.355259	-1.044645
H	-2.055619	2.978591	-0.998608
H	-1.540155	2.125044	0.468296
H	-0.155919	4.383619	-0.852852
H	0.170200	3.642483	0.724312
C	-1.624555	-2.528519	-1.602074
O	-0.903332	-2.036126	-0.493610
C	-1.794834	-2.116569	0.602299
C	-3.164940	-1.680712	0.057517
C	-3.065246	-2.004070	-1.452759
H	-1.118682	-2.190668	-2.508356
H	-1.623383	-3.629886	-1.572161
H	-1.833170	-3.155215	0.961004
H	-1.396763	-1.469122	1.379660
H	-3.977397	-2.224115	0.539652
H	-3.297072	-0.610263	0.219134
H	-3.786719	-2.760960	-1.761318
H	-3.210289	-1.104007	-2.049192
C	3.291517	-0.389001	-1.660609
O	2.233796	-1.064792	-1.010021
C	2.171966	-2.337732	-1.622771
C	2.393522	-2.091836	-3.122877
C	3.180413	-0.761413	-3.149657
H	3.179929	0.674406	-1.451685
H	4.254834	-0.740898	-1.261028
H	2.969401	-2.980229	-1.220497

H	1.200892	-2.767016	-1.386602
H	2.955004	-2.903582	-3.585574
H	1.431218	-1.988248	-3.627466
H	4.169993	-0.866600	-3.595540
H	2.625643	0.001343	-3.692569

Energy -103.581577 (Hartree)

Li-Cluster-3.1-COMPLAX-DFT

Atom	X	Y	Z (Angstrom)
Li	0.368641	-0.319089	-0.331655
O	1.024728	0.411348	1.127091
C	1.520388	0.277867	2.374442
C	1.981509	1.665314	2.917500
Si	2.629377	1.954560	4.676923
C	3.400294	3.683858	4.677110
N	2.667918	-0.669335	2.447817
C	2.434010	-1.951891	1.806736
C	3.795925	-2.646361	1.873922
C	4.803070	-1.474784	1.861807
C	3.921031	-0.218208	1.869804
C	0.432652	-0.249499	3.350272
C	-0.862627	0.270667	3.243049
C	-1.878158	-0.125717	4.110223
C	-1.624719	-1.081035	5.096195
C	-0.342450	-1.614062	5.210853
C	0.676580	-1.192347	4.353954
C	3.949835	0.724252	5.231688
C	1.214409	1.968583	5.925995
H	-1.059438	1.000954	2.455384
H	1.687416	-1.591364	4.461224
H	-2.876885	0.311393	4.016778
H	-0.127209	-2.359866	5.981302
H	-2.420470	-1.401774	5.773697
H	1.098325	2.323150	2.829365
H	2.702285	2.077621	2.190618
H	2.124432	-1.813712	0.748954
H	1.633246	-2.517705	2.306953
H	3.771367	0.160032	0.834527
H	4.371238	0.597993	2.451067
H	3.887298	-3.225564	2.804910
H	3.942425	-3.352406	1.040839
H	5.441686	-1.502102	2.757050
H	5.475182	-1.495268	0.989724
H	0.749936	0.977753	6.039975
H	1.576356	2.293001	6.915374
H	0.424651	2.669424	5.610483
H	2.665171	4.448677	4.378120
H	3.775522	3.954491	5.677458
H	4.247607	3.742860	3.974707
H	3.639475	-0.306790	5.007682
H	4.905944	0.902874	4.714033
H	4.136078	0.814166	6.313901
C	0.417373	2.224413	-1.434332
O	-0.587957	1.218985	-1.251889
C	-1.617366	1.700869	-0.388948
C	-1.053233	2.958295	0.256195
C	-0.174961	3.508261	-0.866120
H	1.309554	1.929033	-0.855662
H	0.665520	2.282586	-2.505143
H	-2.522533	1.915126	-0.987735
H	-1.858550	0.910227	0.338954
H	-1.831929	3.653977	0.599533
H	-0.416740	2.664782	1.104456

H	-0.783501	4.031288	-1.622255
H	0.599950	4.202277	-0.511269
C	-2.075421	-1.811807	-1.218300
O	-1.026009	-1.712074	-0.272701
C	-1.428402	-2.423977	0.888074
C	-2.912351	-2.091664	1.044249
C	-3.376876	-1.790529	-0.397442
H	-1.969628	-0.974045	-1.922018
H	-1.976623	-2.759314	-1.781460
H	-1.267317	-3.507654	0.729742
H	-0.805964	-2.085450	1.726993
H	-3.470643	-2.912965	1.514746
H	-3.024317	-1.208585	1.689096
H	-4.093302	-2.535504	-0.772340
H	-3.868065	-0.808938	-0.458898
C	2.819349	-0.507347	-1.993837
O	1.538927	-1.084351	-1.755841
C	1.538654	-2.477440	-2.061307
C	2.913505	-2.775535	-2.658677
C	3.783587	-1.683994	-2.034834
H	2.804953	0.040603	-2.954106
H	3.027072	0.205732	-1.181839
H	1.361210	-3.041703	-1.129775
H	0.710529	-2.694752	-2.754205
H	3.257638	-3.793822	-2.429129
H	2.891235	-2.667647	-3.754893
H	4.079564	-1.962375	-1.011413
H	4.696833	-1.468146	-2.606862

Energy -1709.100769 (Hartree)

Li-Cluster-3.2-COMPLAX-Best

Atom	X	Y	Z (Angstrom)
Li	0.992401	-0.265918	0.402254
O	-0.046658	0.167552	1.733473
C	0.962800	0.089183	2.645681
C	1.582525	1.500735	2.892829
Si	2.396958	2.162122	4.479471
C	2.938467	3.915305	4.021139
N	2.021405	-0.796974	2.031175
C	1.665422	-2.230506	1.910582
C	2.970157	-2.991157	2.165062
C	4.043242	-1.967746	1.789681
C	3.451228	-0.674583	2.342310
C	0.448222	-0.458501	3.995133
C	-0.872003	-0.171570	4.357936
C	-1.376636	-0.555188	5.601173
C	-0.569153	-1.251842	6.502206
C	0.741728	-1.568574	6.141591
C	1.243784	-1.173594	4.899719
C	3.924908	1.274167	5.146616
C	1.128690	2.293441	5.870232
H	-1.494307	0.357628	3.635613
H	2.268817	-1.440025	4.638243
H	-2.407782	-0.313326	5.867550
H	1.378393	-2.128930	6.829327
H	-0.961475	-1.555780	7.474416
H	0.709635	2.172923	2.810394
H	2.217644	1.758239	2.026098
H	1.282657	-2.465555	0.896625
H	0.867439	-2.508297	2.614741
H	3.883968	0.223695	1.878511
H	3.646566	-0.610753	3.424744
H	3.054293	-3.235408	3.235542

H	3.032916	-3.929291	1.598183
H	5.032509	-2.193865	2.209216
H	4.140108	-1.900169	0.694278
H	0.827919	1.304284	6.247672
H	1.552771	2.871249	6.706824
H	0.223100	2.813456	5.520272
H	2.086258	4.504850	3.648899
H	3.358620	4.436817	4.895145
H	3.708082	3.896686	3.233635
H	3.699797	0.260986	5.509829
H	4.722055	1.208492	4.391273
H	4.310998	1.858507	5.997254
C	-0.062555	-0.742253	-2.158315
O	0.980145	-1.158726	-1.302014
C	1.671848	-2.164749	-2.007661
C	0.584846	-3.011115	-2.690757
C	-0.597564	-2.024860	-2.818599
H	-0.794086	-0.212432	-1.546766
H	0.341627	-0.059921	-2.919435
H	2.327137	-1.703012	-2.762068
H	2.277413	-2.723541	-1.293254
H	0.913412	-3.379592	-3.661860
H	0.320126	-3.859873	-2.056958
H	-0.880707	-1.838791	-3.854054
H	-1.465719	-2.381563	-2.269024
C	2.971768	1.650573	-1.109237
O	1.727766	1.377888	-0.489230
C	1.030691	2.603732	-0.487854
C	2.077084	3.679535	-0.144889
C	3.407121	3.033398	-0.591031
H	3.654004	0.838084	-0.856037
H	2.831423	1.687050	-2.199557
H	0.605346	2.785469	-1.485741
H	0.231772	2.520835	0.245000
H	1.874067	4.609564	-0.672138
H	2.070279	3.871938	0.930014
H	3.909583	3.597975	-1.378126
H	4.085721	2.929974	0.255355
C	-2.352279	-2.254329	1.170085
O	-2.570685	-1.602349	-0.064486
C	-3.162657	-0.362126	0.264001
C	-4.172175	-0.671716	1.384451
C	-3.584707	-1.942156	2.040566
H	-2.212440	-3.317595	0.961432
H	-1.446041	-1.850676	1.640798
H	-2.391379	0.330517	0.624625
H	-3.617357	0.035476	-0.644941
H	-4.252166	0.152106	2.091167
H	-5.156765	-0.873917	0.961119
H	-3.285378	-1.771752	3.073265
H	-4.301513	-2.762263	2.009508

Energy -103.581495 (Hartree)

Li-Cluster-3.2-COMPLAX-DFT

Atom	X	Y	Z (Angstrom)
Li	1.126726	-0.119458	0.203133
O	0.070287	0.224633	1.658210
C	1.027487	0.083930	2.603009
C	1.702630	1.466543	2.898662
Si	2.504653	2.105780	4.496100
C	3.130010	3.832951	4.032028
N	2.061079	-0.854958	2.038065
C	1.639004	-2.256259	1.882922

C	2.939566	-3.073415	1.907290
C	4.031811	-2.035589	1.638836
C	3.481722	-0.809862	2.359189
C	0.417088	-0.449977	3.923631
C	-0.919250	-0.140512	4.192822
C	-1.521833	-0.526139	5.388809
C	-0.799205	-1.249681	6.337963
C	0.528509	-1.583489	6.074506
C	1.128652	-1.183008	4.880032
C	3.986414	1.157118	5.184012
C	1.225517	2.299544	5.865474
H	-1.466735	0.408053	3.423469
H	2.169581	-1.458215	4.695141
H	-2.566165	-0.263637	5.581351
H	1.103892	-2.160471	6.803730
H	-1.269689	-1.557497	7.275416
H	0.855303	2.168829	2.799512
H	2.362762	1.708409	2.047060
H	1.081391	-2.395322	0.939641
H	0.949556	-2.572862	2.683215
H	3.942946	0.128297	2.012729
H	3.699948	-0.891499	3.441519
H	3.088752	-3.511874	2.907315
H	2.937643	-3.909421	1.190998
H	5.024885	-2.339771	2.001454
H	4.117228	-1.827615	0.558441
H	0.871091	1.326943	6.239749
H	1.653816	2.856655	6.714699
H	0.345497	2.856400	5.506233
H	2.306362	4.474396	3.678124
H	3.593433	4.337052	4.895426
H	3.885860	3.785449	3.230582
H	3.711733	0.152806	5.539463
H	4.796149	1.054200	4.445067
H	4.392611	1.710406	6.046622
C	0.124475	-0.947865	-2.307949
O	1.212846	-1.248550	-1.431693
C	1.681315	-2.542971	-1.763443
C	0.409532	-3.339855	-2.024425
C	-0.516043	-2.297583	-2.677463
H	-0.565949	-0.289087	-1.765752
H	0.512525	-0.413501	-3.193248
H	2.320734	-2.491028	-2.666194
H	2.291256	-2.914091	-0.928385
H	0.583624	-4.224395	-2.653316
H	-0.011428	-3.681206	-1.066598
H	-0.560360	-2.425505	-3.769116
H	-1.533839	-2.356383	-2.271903
C	2.624896	2.124423	-1.316907
O	1.463796	1.608742	-0.700363
C	0.658422	2.710409	-0.291252
C	1.643184	3.844440	0.051111
C	3.005389	3.329491	-0.457403
H	3.382094	1.327987	-1.345683
H	2.399027	2.426811	-2.358182
H	-0.009347	2.997642	-1.123975
H	0.055721	2.349182	0.554524
H	1.350903	4.782880	-0.441603
H	1.673397	4.037017	1.132393
H	3.570997	4.085011	-1.020956
H	3.635445	3.003916	0.384296
C	-2.083356	-2.129588	1.028881
O	-2.237901	-1.378395	-0.161955

C	-2.764836	-0.129020	0.248100
C	-3.779733	-0.452222	1.360765
C	-3.356905	-1.858180	1.837734
H	-1.947491	-3.187009	0.755737
H	-1.199171	-1.759731	1.575752
H	-1.948136	0.496936	0.648312
H	-3.215440	0.356215	-0.631043
H	-3.734480	0.294666	2.166831
H	-4.810635	-0.459050	0.977048
H	-3.161271	-1.901327	2.918998
H	-4.136499	-2.599519	1.606574

Energy -1709.081066 (Hartree)

Li-Cluster-3.3-COMPLAX-Best

Atom	X	Y	Z (Angstrom)
Li	0.992401	-0.265918	0.402254
O	-0.046658	0.167552	1.733473
C	0.962800	0.089183	2.645681
C	1.582525	1.500735	2.892829
Si	2.396958	2.162122	4.479471
C	2.938467	3.915305	4.021139
N	2.021405	-0.796974	2.031175
C	1.665422	-2.230506	1.910582
C	2.970157	-2.991157	2.165062
C	4.043242	-1.967746	1.789681
C	3.451228	-0.674583	2.342310
C	0.448222	-0.458501	3.995133
C	-0.872003	-0.171570	4.357936
C	-1.376636	-0.555188	5.601173
C	-0.569153	-1.251842	6.502206
C	0.741728	-1.568574	6.141591
C	1.243784	-1.173594	4.899719
C	3.924908	1.274167	5.146616
C	1.128690	2.293441	5.870232
H	-1.494307	0.357628	3.635613
H	2.268817	-1.440025	4.638243
H	-2.407782	-0.313326	5.867550
H	1.378393	-2.128930	6.829327
H	-0.961475	-1.555780	7.474416
H	0.709635	2.172923	2.810394
H	2.217644	1.758239	2.026098
H	1.282657	-2.465555	0.896625
H	0.867439	-2.508297	2.614741
H	3.883968	0.223695	1.878511
H	3.646566	-0.610753	3.424744
H	3.054293	-3.235408	3.235542
H	3.032916	-3.929291	1.598183
H	5.032509	-2.193865	2.209216
H	4.140108	-1.900169	0.694278
H	0.827919	1.304284	6.247672
H	1.552771	2.871249	6.706824
H	0.223100	2.813456	5.520272
H	2.086258	4.504850	3.648899
H	3.358620	4.436817	4.895145
H	3.708082	3.896686	3.233635
H	3.699797	0.260986	5.509829
H	4.722055	1.208492	4.391273
H	4.310998	1.858507	5.997254
C	0.522820	2.531619	-0.880772
O	0.545449	1.127397	-1.032799
C	-0.531758	0.820925	-1.895142
C	-1.689447	1.720672	-1.435314
C	-0.962632	2.927678	-0.798446

H	1.102412	2.775472	0.010998
H	0.994889	2.998495	-1.760197
H	-0.248580	1.058795	-2.931013
H	-0.730492	-0.243243	-1.799769
H	-2.337095	2.018457	-2.258494
H	-2.277045	1.199838	-0.682306
H	-1.149918	3.858336	-1.332032
H	-1.263112	3.042115	0.240379
C	3.331524	-0.158149	-1.520085
O	2.321534	-0.947915	-0.926853
C	2.211413	-2.095151	-1.742073
C	2.314264	-1.584453	-3.188088
C	3.122065	-0.274281	-3.040014
H	3.229925	0.854546	-1.127922
H	4.320183	-0.560073	-1.248082
H	3.037989	-2.787311	-1.521563
H	1.261802	-2.571449	-1.509050
H	2.815837	-2.300229	-3.838043
H	1.317718	-1.383630	-3.580529
H	4.078472	-0.315124	-3.560968
H	2.550248	0.573501	-3.412115
C	-1.526727	-3.435150	-1.404874
O	-0.941267	-2.406132	-0.633427
C	-1.927229	-2.025714	0.305586
C	-3.240114	-1.978914	-0.494850
C	-3.009812	-3.050978	-1.585221
H	-0.972026	-3.507631	-2.344567
H	-1.446932	-4.391901	-0.863198
H	-1.994782	-2.792954	1.094063
H	-1.601888	-1.082135	0.744097
H	-4.105837	-2.206541	0.125527
H	-3.375678	-1.003999	-0.956587
H	-3.655377	-3.912125	-1.432178
H	-3.188015	-2.640016	-2.578403

Energy -103.581463 (Hartree)

Li-Cluster-3.3-COMPLAX-DFT

Atom	X	Y	Z (Angstrom)
Li	0.430155	-0.899612	-0.536176
O	0.687253	-0.250051	1.078681
C	1.415239	-0.180814	2.211893
C	2.140690	1.205573	2.296074
Si	2.630772	2.191882	3.842747
C	3.546255	3.704937	3.165741
N	2.425931	-1.257753	2.218796
C	1.951118	-2.618191	2.035015
C	3.086465	-3.500361	2.569806
C	4.314017	-2.592237	2.479903
C	3.730839	-1.227538	2.842533
C	0.467741	-0.307131	3.442124
C	-0.816002	0.243536	3.359378
C	-1.693066	0.207305	4.440625
C	-1.305928	-0.399222	5.636552
C	-0.038214	-0.970484	5.729552
C	0.836562	-0.923554	4.642114
C	3.774844	1.361494	5.094006
C	1.106818	2.802992	4.771093
H	-1.107513	0.701653	2.412556
H	1.823083	-1.383291	4.730782
H	-2.687119	0.655139	4.351621
H	0.274357	-1.461089	6.655644
H	-1.990550	-0.431607	6.488220
H	1.419348	1.895222	1.821506

H	2.997467	1.179367	1.599312
H	1.740120	-2.832669	0.967871
H	1.006168	-2.807262	2.578257
H	4.350185	-0.397243	2.461413
H	3.711442	-1.119016	3.947850
H	2.897472	-3.759978	3.624939
H	3.195320	-4.444766	2.015728
H	5.137720	-2.897292	3.142893
H	4.703125	-2.574167	1.448128
H	0.587302	1.982008	5.289136
H	1.395128	3.554290	5.524511
H	0.382941	3.273192	4.086248
H	2.919448	4.262181	2.450639
H	3.826610	4.398653	3.974809
H	4.470367	3.412547	2.640935
H	3.295543	0.497681	5.578620
H	4.720232	1.024963	4.641715
H	4.022781	2.088801	5.884553
C	-0.019447	1.825708	-1.077160
O	-0.507407	0.590248	-1.604604
C	-1.922044	0.635349	-1.578551
C	-2.247903	1.331542	-0.264043
C	-1.122085	2.371005	-0.150915
H	0.904014	1.596840	-0.528666
H	0.194701	2.517264	-1.911710
H	-2.301931	1.205705	-2.448890
H	-2.296029	-0.395348	-1.644207
H	-3.254335	1.773761	-0.248042
H	-2.173902	0.603083	0.557270
H	-1.459576	3.364508	-0.482157
H	-0.762248	2.464847	0.882819
C	3.256956	-0.977616	-1.045049
O	2.007444	-1.136304	-1.706926
C	2.138769	-0.602593	-3.013405
C	3.143251	0.561095	-2.899776
C	3.741245	0.401209	-1.487073
H	3.074373	-1.059177	0.038742
H	3.951428	-1.775700	-1.372314
H	2.512363	-1.387239	-3.696940
H	1.138542	-0.289937	-3.343097
H	3.911904	0.494392	-3.683544
H	2.645226	1.534152	-3.015885
H	4.837196	0.484529	-1.471398
H	3.340547	1.165409	-0.804408
C	-1.589752	-3.178980	-1.426302
O	-0.889761	-2.376147	-0.496830
C	-1.461882	-2.620446	0.783620
C	-2.963433	-2.664317	0.510019
C	-3.049581	-3.217276	-0.929278
H	-1.465944	-2.734790	-2.424691
H	-1.149936	-4.193078	-1.446147
H	-1.095756	-3.590252	1.170978
H	-1.114671	-1.817652	1.448133
H	-3.501365	-3.283385	1.241277
H	-3.385684	-1.649604	0.565100
H	-3.446543	-4.242274	-0.957014
H	-3.707967	-2.601207	-1.559046

Energy -1709.092525 (Hartree)

Li-Cluster-ABCluster-Best

Atom	X	Y	Z (Angstrom)
Li	5.737171	3.465922	2.934957
O	5.958307	5.000535	2.174871

C	5.926611	6.354640	2.472481
C	7.096219	6.891144	1.657144
Si	7.131057	5.461164	0.390817
C	8.138976	3.859043	0.704227
N	6.155092	6.537082	3.922625
C	5.084331	6.090799	4.793872
C	4.230674	7.321590	5.149864
C	5.201972	8.498220	4.994586
C	6.482982	7.869014	4.398880
C	4.595144	6.976051	2.059485
C	3.467654	6.170023	1.952626
C	2.232316	6.699890	1.631224
C	2.098174	8.058499	1.403220
C	3.212580	8.873500	1.499264
C	4.446564	8.338139	1.824225
C	8.286641	6.215246	-0.983265
C	5.549882	5.225173	-0.670178
H	3.577662	5.107920	2.119124
H	5.297542	8.999615	1.890096
H	1.369093	6.051197	1.556594
H	3.119874	9.935883	1.318651
H	1.135344	8.478428	1.148115
H	7.995033	6.896668	2.269868
H	6.966427	7.882190	1.236636
H	5.539950	5.689056	5.707147
H	4.520474	5.298793	4.310446
H	7.235568	7.758731	5.190720
H	6.916770	8.474835	3.606944
H	3.396583	7.417803	4.455487
H	3.830167	7.247459	6.160879
H	4.791594	9.253046	4.326075
H	5.417965	8.973492	5.951189
H	5.223136	6.188556	-1.047113
H	5.753513	4.596031	-1.531276
H	4.720177	4.801025	-0.118665
H	7.734894	3.239006	1.494208
H	8.182367	3.273703	-0.208092
H	9.160983	4.117363	0.959272
H	7.874409	7.122112	-1.416511
H	9.272089	6.466266	-0.600484
H	8.433188	5.512833	-1.798207
C	4.776397	0.582752	2.349758
O	5.427829	1.770788	1.959580
C	5.394218	1.762066	0.545456
C	3.972346	1.318423	0.196841
C	3.610242	0.385441	1.363943
H	5.477090	-0.263288	2.276907
H	4.457339	0.695664	3.382510
H	5.648365	2.757259	0.196698
H	6.126221	1.033967	0.166569
H	3.311087	2.183664	0.172881
H	3.924372	0.820917	-0.768847
H	2.661545	0.668730	1.815664
H	3.537289	-0.653821	1.047452
C	7.758679	1.760062	4.468574
O	7.025419	2.956328	4.343656
C	7.898221	3.979521	4.790581
C	9.284557	3.606479	4.254299
C	9.178859	2.094192	3.997834
H	7.772378	1.440773	5.523318
H	7.271559	0.993738	3.869755
H	7.521569	4.924359	4.401360
H	7.900593	3.996916	5.890955

H	9.480608	4.141168	3.328490
H	10.066839	3.849663	4.971064
H	9.283686	1.878485	2.936288
H	9.927420	1.523283	4.544125
C	3.910660	2.663660	5.682036
O	3.831316	2.946213	4.303592
C	2.454342	3.069039	4.022781
C	1.767779	1.954636	4.830600
C	2.765061	1.678343	5.969515
H	4.901268	2.261731	5.882056
H	3.772614	3.588501	6.258767
H	2.093460	4.054894	4.348764
H	2.320621	2.977695	2.946408
H	0.795959	2.274021	5.199474
H	1.625988	1.065536	4.217539
H	2.330460	1.852363	6.951139
H	3.123007	0.650318	5.931221

Energy 0.000000 (Hartree)

Li-Cluster-ABCluster-DFT

Atom	X	Y	Z (Angstrom)
Li	1.969368	7.009773	1.197874
O	0.763043	5.974053	1.761171
C	0.320718	4.700618	1.894891
C	1.530142	3.754263	2.096859
Si	2.835538	4.421026	3.286170
C	3.911833	5.771481	2.472408
N	-0.369595	4.300446	0.631591
C	-1.423701	5.185763	0.168160
C	-2.768849	4.547420	0.580727
C	-2.446099	3.045741	0.718967
C	-0.915329	2.961088	0.485945
C	-0.650074	4.607167	3.111246
C	-1.276588	5.773350	3.565824
C	-2.185953	5.751550	4.620442
C	-2.485875	4.550171	5.262421
C	-1.863654	3.379967	4.833403
C	-0.958322	3.411067	3.771552
C	4.001011	2.991920	3.700503
C	2.139954	5.129073	4.883839
H	-1.019203	6.708824	3.067701
H	-0.486497	2.474580	3.464546
H	-2.661135	6.680623	4.948616
H	-2.081165	2.430490	5.330431
H	-3.194244	4.527062	6.094647
H	2.011593	3.643383	1.109039
H	1.257001	2.733130	2.408629
H	-1.374113	5.261884	-0.934910
H	-1.265889	6.196074	0.568008
H	-0.723560	2.605922	-0.543961
H	-0.417875	2.244851	1.154237
H	-3.108502	4.956311	1.543793
H	-3.562076	4.745673	-0.156636
H	-2.717646	2.678645	1.719470
H	-2.992785	2.429016	-0.011100
H	1.549452	4.379830	5.433747
H	2.948633	5.482382	5.544600
H	1.466330	5.969844	4.659831
H	3.510650	6.777875	2.677643
H	4.932588	5.762748	2.886482
H	4.013582	5.623575	1.382801
H	3.458681	2.176618	4.205870
H	4.458672	2.570502	2.790668

H	4.816067	3.314039	4.368588
C	0.674185	9.106284	2.352278
O	1.972251	8.836504	1.809319
C	2.900115	9.556176	2.610601
C	2.396336	9.375429	4.044297
C	0.876102	9.161975	3.872110
H	0.314053	10.068829	1.948693
H	0.012572	8.292295	2.024901
H	3.904878	9.149917	2.426635
H	2.893429	10.619063	2.307302
H	2.860257	8.490220	4.503889
H	2.641751	10.241666	4.674267
H	0.557029	8.218772	4.337080
H	0.284385	9.971286	4.322171

Energy -1244.588460 (Hartree)

Li-Cluster-OGOLEM-Best

Atom	X	Y	Z (Angstrom)
Li	-1.370213	-0.445063	-0.690975
O	-0.184552	0.328423	0.214584
C	1.067423	-0.002180	0.697237
C	1.957834	1.055972	0.058623
Si	0.594490	2.367032	-0.243627
C	-0.452221	2.335679	-1.854276
N	1.415349	-1.363206	0.237079
C	0.635169	-2.453594	0.803004
C	1.322382	-3.721672	0.277908
C	2.735273	-3.251728	-0.110835
C	2.786767	-1.808608	0.397128
C	1.105357	0.042263	2.223448
C	-0.074817	-0.138278	2.935265
C	-0.084058	-0.178390	4.316555
C	1.101777	-0.050842	5.019603
C	2.285811	0.123888	4.325647
C	2.288206	0.171027	2.941968
C	1.673950	3.923821	-0.677453
C	-0.419273	3.035776	1.237045
H	-0.993294	-0.246111	2.376372
H	3.226862	0.314617	2.427726
H	-1.016533	-0.314728	4.847369
H	3.217083	0.224057	4.865415
H	1.102902	-0.085865	6.099496
H	2.338538	0.683837	-0.890324
H	2.806028	1.376138	0.654148
H	-0.407217	-2.375248	0.485424
H	0.665221	-2.443744	1.904653
H	3.469798	-1.182854	-0.176125
H	3.101680	-1.802995	1.454052
H	1.350184	-4.492154	1.047316
H	0.793787	-4.123818	-0.585053
H	3.516882	-3.864452	0.335044
H	2.859897	-3.269988	-1.193526
H	-0.807545	2.246869	1.868208
H	0.179955	3.686698	1.861128
H	-1.256714	3.629018	0.881241
H	-1.226096	1.574340	-1.868697
H	-0.927378	3.302820	-1.979603
H	0.189534	2.187630	-2.716139
H	2.445366	4.117192	0.062684
H	2.177741	3.814368	-1.633720
H	1.059308	4.816167	-0.742786
C	-4.203010	-0.970300	0.132772
O	-3.236039	-0.083978	-0.388501

C	-3.495662	1.161750	0.242076
C	-3.930207	0.832693	1.679164
C	-4.229526	-0.674071	1.632298
H	-5.183418	-0.753921	-0.318599
H	-3.908206	-1.989778	-0.110244
H	-2.583385	1.749321	0.197642
H	-4.301631	1.676178	-0.299529
H	-3.132432	1.055820	2.383552
H	-4.807772	1.410437	1.964145
H	-3.441768	-1.238455	2.131056
H	-5.184219	-0.930038	2.086173
C	0.050847	-1.856779	-2.649642
O	-1.268672	-1.613243	-2.189161
C	-2.040852	-1.460934	-3.361313
C	-1.179804	-0.624947	-4.315315
C	0.251899	-0.890123	-3.819110
H	0.730285	-1.677978	-1.816962
H	0.125573	-2.900126	-2.989841
H	-2.244886	-2.451604	-3.794577
H	-2.982194	-0.985415	-3.095430
H	-1.325274	-0.926387	-5.350839
H	-1.428865	0.430394	-4.223876
H	0.886303	-1.320238	-4.591622
H	0.710638	0.029049	-3.461569
C	1.668857	5.764853	4.097067
O	0.742056	4.744395	4.384218
C	1.486576	3.549292	4.483507
C	2.639957	3.658275	3.468012
C	2.663632	5.151180	3.103122
H	1.116577	6.615063	3.696382
H	2.188371	6.073894	5.018314
H	1.883404	3.443825	5.504798
H	0.812169	2.718719	4.278122
H	3.578333	3.332008	3.913404
H	2.437893	3.043487	2.593598
H	3.653105	5.593826	3.198383
H	2.309285	5.299420	2.084134

Energy -271916.940200 (Hartree)

Li-Cluster-OGOLEM-DFT

Atom	X	Y	Z (Angstrom)
Li	-1.685655	-0.035468	-0.902299
O	-0.675125	0.059129	0.510090
C	0.627091	-0.042229	0.853251
C	1.458511	1.026122	0.098139
Si	0.486762	2.623177	-0.211546
C	-0.785332	2.419137	-1.614962
N	1.116936	-1.396283	0.444259
C	0.358222	-2.530940	0.941565
C	1.121586	-3.754671	0.400804
C	2.519351	-3.208786	0.031412
C	2.507333	-1.781013	0.585155
C	0.806441	0.107191	2.392374
C	-0.323427	0.032922	3.212875
C	-0.219252	0.077150	4.602075
C	1.031504	0.194694	5.207726
C	2.168272	0.281257	4.406328
C	2.053723	0.245703	3.015783
C	1.748401	3.883438	-0.854136
C	-0.398249	3.345087	1.282435
H	-1.289761	-0.055687	2.713200
H	2.961881	0.333980	2.414394
H	-1.121133	0.020603	5.218407

H	3.155414	0.387134	4.865238
H	1.119180	0.229129	6.296806
H	1.694225	0.623402	-0.904517
H	2.431275	1.256904	0.564091
H	-0.684283	-2.477282	0.596382
H	0.331605	-2.555485	2.052803
H	3.174556	-1.097460	0.037572
H	2.840832	-1.797877	1.645845
H	1.174939	-4.554234	1.154495
H	0.618839	-4.182699	-0.479878
H	3.341010	-3.810790	0.447288
H	2.651963	-3.187532	-1.061602
H	-0.898472	2.545646	1.848436
H	0.260373	3.890592	1.974231
H	-1.167377	4.059952	0.945607
H	-1.790168	2.185613	-1.225075
H	-0.884669	3.356760	-2.185160
H	-0.488128	1.633500	-2.328506
H	2.564952	4.037685	-0.130219
H	2.207706	3.544550	-1.797523
H	1.280505	4.863359	-1.042981
C	-3.937219	-1.360085	-0.064449
O	-3.562682	-0.014984	-0.358675
C	-3.739768	0.813202	0.805069
C	-4.218585	-0.117856	1.921832
C	-3.752650	-1.492547	1.439161
H	-4.987416	-1.529952	-0.366503
H	-3.296642	-2.029477	-0.660124
H	-2.762079	1.260166	1.043077
H	-4.461730	1.610541	0.569567
H	-3.797553	0.162427	2.897651
H	-5.316315	-0.092972	2.011562
H	-2.685134	-1.636435	1.666876
H	-4.324537	-2.326626	1.869765
C	-0.107928	-1.676512	-2.675813
O	-1.348287	-0.979888	-2.553447
C	-1.790134	-0.675679	-3.867068
C	-0.515172	-0.370524	-4.672568
C	0.623512	-0.941441	-3.798365
H	0.399113	-1.635707	-1.696363
H	-0.312412	-2.729579	-2.947150
H	-2.328625	-1.547251	-4.281693
H	-2.494104	0.166361	-3.803797
H	-0.555772	-0.838399	-5.666537
H	-0.392837	0.710870	-4.827723
H	1.298436	-1.606889	-4.354507
H	1.236063	-0.130361	-3.377126
C	2.139423	5.889554	3.761177
O	1.105287	4.981858	4.044428
C	1.723671	3.738099	4.288931
C	2.893805	3.650808	3.290410
C	3.118352	5.117526	2.866696
H	1.702126	6.778075	3.283582
H	2.638507	6.214073	4.698144
H	2.093765	3.694717	5.333150
H	0.975147	2.943655	4.160997
H	3.784972	3.198446	3.749192
H	2.618002	3.021878	2.432023
H	4.157750	5.452062	2.997790
H	2.855870	5.261143	1.808215

Energy 0.000000 (Hartree)

Li-Cluster-CREST-Best

Atom	X	Y	Z (Angstrom)
Li	-1.005934	1.212498	-3.508839
O	-0.770191	0.591074	-2.024959
C	-0.344456	-0.103555	-0.941244
C	0.867992	-1.011349	-1.326571
Si	2.221409	-1.535713	-0.078351
C	2.442036	-3.437198	-0.217868
N	-1.423218	-1.005291	-0.526868
C	-2.780731	-0.470421	-0.509861
C	-3.482685	-1.111571	0.690624
C	-2.647793	-2.365714	0.939080
C	-1.235105	-1.890628	0.606095
C	0.075716	0.954437	0.091691
C	1.175372	1.751033	-0.221793
C	1.554547	2.807436	0.583306
C	0.832814	3.105666	1.727967
C	-0.275300	2.340877	2.041427
C	-0.652319	1.282929	1.228673
C	2.029039	-1.140619	1.782294
C	3.880362	-0.737415	-0.617001
H	1.730931	1.536222	-1.121095
H	-1.524716	0.712646	1.502046
H	2.417718	3.402699	0.319285
H	-0.856504	2.565503	2.925611
H	1.127686	3.928459	2.363253
H	1.362323	-0.482741	-2.144895
H	0.427957	-1.903069	-1.775759
H	-3.280608	-0.762460	-1.443753
H	-2.781779	0.624375	-0.474321
H	-0.577004	-2.717270	0.325490
H	-0.784621	-1.386305	1.474276
H	-3.440579	-0.453592	1.561044
H	-4.527970	-1.333356	0.481535
H	-2.729706	-2.739655	1.958667
H	-2.945094	-3.152678	0.245606
H	3.896828	0.325458	-0.395732
H	4.718217	-1.190843	-0.095852
H	4.057188	-0.855645	-1.682113
H	2.717091	-3.732241	-1.226074
H	3.222674	-3.788871	0.449665
H	1.531018	-3.969691	0.039887
H	1.654892	-0.135516	1.944382
H	1.353072	-1.831384	2.275808
H	2.991498	-1.215840	2.280669
C	-1.214730	3.818605	-2.534545
O	-1.381685	3.037643	-3.702397
C	-1.441719	3.973280	-4.763969
C	-0.400372	5.053765	-4.429800
C	-0.181411	4.881512	-2.915774
H	-2.173000	4.284947	-2.267252
H	-0.894981	3.155226	-1.735032
H	-1.242197	3.443799	-5.692538
H	-2.449034	4.409103	-4.803625
H	0.524325	4.885634	-4.977865
H	-0.771947	6.045373	-4.678018
H	0.821732	4.513201	-2.706953
H	-0.333412	5.805221	-2.363005
C	-2.543193	-0.560570	-4.766849
O	-1.468990	0.286523	-5.129988
C	-0.590469	-0.555320	-5.855468
C	-0.578388	-1.894136	-5.103840
C	-1.876070	-1.846216	-4.279657
H	-3.124356	-0.063639	-3.994160

H	-3.169919	-0.748102	-5.650932
H	-0.985940	-0.688823	-6.873344
H	0.385194	-0.076951	-5.905839
H	-0.557320	-2.735102	-5.794269
H	0.287515	-1.957443	-4.448154
H	-2.506775	-2.719228	-4.430795
H	-1.648416	-1.744540	-3.219913

Energy -86.874956 (Hartree)

Li-Cluster-CREST-DFT

Atom	X	Y	Z (Angstrom)
Li	-1.278639	1.276793	-3.826298
O	-0.567032	0.576584	-2.453150
C	-0.195518	0.032481	-1.275255
C	1.031002	-0.916820	-1.485369
Si	2.305899	-1.468529	-0.175133
C	2.314000	-3.357111	-0.062189
N	-1.316213	-0.747862	-0.735241
C	-2.651393	-0.178662	-0.775948
C	-3.429862	-0.955447	0.293364
C	-2.649616	-2.264832	0.419443
C	-1.205395	-1.795139	0.254105
C	0.225437	1.192398	-0.326355
C	1.248899	2.054701	-0.747293
C	1.667217	3.123794	0.039359
C	1.047807	3.375809	1.266980
C	0.016612	2.541494	1.689471
C	-0.383645	1.458504	0.901446
C	2.127110	-0.770616	1.567497
C	4.001425	-0.923213	-0.810256
H	1.719410	1.874983	-1.717986
H	-1.178615	0.802975	1.262020
H	2.478378	3.771133	-0.306856
H	-0.478601	2.725580	2.646918
H	1.369841	4.216374	1.887202
H	1.637408	-0.392705	-2.242583
H	0.648207	-1.813783	-2.003930
H	-3.112899	-0.302579	-1.775892
H	-2.661913	0.907830	-0.571271
H	-0.546019	-2.609876	-0.087388
H	-0.805283	-1.454041	1.234911
H	-3.397441	-0.413741	1.254020
H	-4.490372	-1.095897	0.035837
H	-2.826697	-2.793939	1.367835
H	-2.914658	-2.948866	-0.403835
H	4.047553	0.175868	-0.880541
H	4.810910	-1.250366	-0.138391
H	4.207881	-1.333075	-1.812177
H	2.445581	-3.813238	-1.056768
H	3.140802	-3.706252	0.577148
H	1.377675	-3.749942	0.364537
H	2.243069	0.323909	1.579922
H	1.156045	-1.007722	2.026391
H	2.915572	-1.203827	2.205047
C	-1.984359	3.517748	-2.340915
O	-1.726257	3.178437	-3.718674
C	-0.901602	4.173545	-4.312808
C	-0.076862	4.723921	-3.159174
C	-1.097569	4.725569	-2.018891
H	-3.057998	3.733186	-2.219953
H	-1.714930	2.633633	-1.744629
H	-0.307290	3.699588	-5.108755
H	-1.529249	4.958767	-4.774602

```
  H     0.751225     4.036409    -2.927499
  H     0.345766     5.716687    -3.369537
  H    -0.626187     4.628266    -1.030580
  H    -1.683806     5.657974    -2.028185
  C    -2.707442    -0.900836    -4.792232
  O    -1.585082    -0.114173    -5.196372
  C    -0.407603    -0.939280    -5.260605
  C    -0.853508    -2.351153    -4.880143
  C    -2.110802    -2.087097    -4.050791
  H    -3.364892    -0.272694    -4.170047
  H    -3.279528    -1.213853    -5.685404
  H     0.015883    -0.875590    -6.275154
  H     0.312542    -0.544508    -4.526447
  H    -1.094551    -2.943191    -5.777986
  H    -0.074674    -2.888694    -4.320940
  H    -2.794038    -2.947340    -4.004612
  H    -1.834604    -1.784009    -3.026952
Energy            0.000000  (Hartree)
```

Li-Cluster-SOLVATOR-Best

```
 Atom       X            Y             Z (Angstrom)
 Li     0.992401    -0.265918     0.402254
  O    -0.046658     0.167552     1.733473
  C     0.962800     0.089183     2.645681
  C     1.582525     1.500735     2.892829
 Si     2.396958     2.162122     4.479471
  C     2.938467     3.915305     4.021139
  N     2.021405    -0.796974     2.031175
  C     1.665422    -2.230506     1.910582
  C     2.970157    -2.991157     2.165062
  C     4.043242    -1.967746     1.789681
  C     3.451228    -0.674583     2.342310
  C     0.448222    -0.458501     3.995133
  C    -0.872003    -0.171570     4.357936
  C    -1.376636    -0.555188     5.601172
  C    -0.569153    -1.251842     6.502206
  C     0.741728    -1.568574     6.141591
  C     1.243784    -1.173594     4.899719
  C     3.924908     1.274167     5.146616
  C     1.128690     2.293441     5.870232
  H    -1.494307     0.357628     3.635613
  H     2.268817    -1.440025     4.638243
  H    -2.407782    -0.313326     5.867550
  H     1.378393    -2.128930     6.829327
  H    -0.961475    -1.555780     7.474416
  H     0.709635     2.172923     2.810394
  H     2.217644     1.758239     2.026098
  H     1.282657    -2.465555     0.896625
  H     0.867439    -2.508297     2.614741
  H     3.883968     0.223695     1.878511
  H     3.646566    -0.610753     3.424744
  H     3.054293    -3.235408     3.235542
  H     3.032916    -3.929291     1.598183
  H     5.032509    -2.193865     2.209216
  H     4.140108    -1.900169     0.694278
  H     0.827919     1.304284     6.247672
  H     1.552771     2.871249     6.706824
  H     0.223100     2.813456     5.520272
  H     2.086258     4.504850     3.648899
  H     3.358620     4.436817     4.895145
  H     3.708082     3.896686     3.233635
  H     3.699797     0.260986     5.509829
  H     4.722055     1.208492     4.391273
```

H	4.310998	1.858507	5.997254
C	3.727155	-0.536437	-2.531382
C	3.792756	0.809695	-1.814038
C	2.314170	1.091002	-1.618588
O	1.766870	-0.162585	-1.228110
C	2.486582	-1.190224	-1.912529
H	3.581621	-0.378255	-3.598956
H	4.621477	-1.136535	-2.389797
H	4.277098	0.715657	-0.843145
H	4.298192	1.576179	-2.393755
H	1.855256	1.417640	-2.561736
H	2.100293	1.809783	-0.832260
H	2.751835	-1.955505	-1.182497
H	1.837206	-1.642841	-2.668853
C	5.191737	5.123710	0.734039
C	3.712056	5.539027	0.674941
C	2.995902	4.255593	0.232146
O	3.829470	3.181710	0.622304
C	5.164389	3.613104	0.438005
H	5.791598	5.648764	-0.005009
H	5.611301	5.317609	1.719241
H	3.358559	5.847984	1.656472
H	3.534956	6.348163	-0.028532
H	2.866611	4.249568	-0.860591
H	2.027375	4.109739	0.711441
H	5.793277	3.029380	1.111466
H	5.475732	3.420713	-0.598705
C	-3.440238	-3.557916	2.621882
C	-2.844718	-3.982230	1.276416
C	-1.369756	-3.643508	1.474152
O	-1.096195	-3.981861	2.821740
C	-2.241767	-3.642610	3.589118
H	-3.807690	-2.534265	2.570669
H	-4.254589	-4.207286	2.934381
H	-2.956756	-5.056661	1.130450
H	-3.280076	-3.452519	0.433877
H	-1.196259	-2.568841	1.308175
H	-0.693680	-4.229941	0.850738
H	-2.360730	-4.421919	4.346988
H	-2.072411	-2.683011	4.094731

Energy 0.000000 (Hartree)

Li-Cluster-SOLVATOR-DFT

Atom	X	Y	Z (Angstrom)
Li	0.697608	-0.332089	0.096938
O	-0.317672	0.003297	1.506512
C	0.694522	0.096148	2.400470
C	1.095277	1.598696	2.604928
Si	1.762979	2.443163	4.166154
C	1.976757	4.258702	3.669227
N	1.870823	-0.642139	1.812851
C	1.721172	-2.099468	1.670244
C	3.132578	-2.668858	1.838712
C	4.036468	-1.483254	1.497799
C	3.264862	-0.310325	2.093694
C	0.262786	-0.492742	3.766582
C	-1.088078	-0.388379	4.115130
C	-1.548757	-0.833744	5.352246
C	-0.665650	-1.414065	6.263975
C	0.679376	-1.543278	5.921688
C	1.137279	-1.081780	4.686096
C	3.446632	1.862580	4.801207
C	0.532552	2.386152	5.590834

H	-1.760317	0.043616	3.370229
H	2.194770	-1.198089	4.440290
H	-2.608084	-0.734198	5.606260
H	1.380275	-2.008681	6.620022
H	-1.024914	-1.770412	7.232935
H	0.135231	2.122373	2.450741
H	1.727217	1.914472	1.754589
H	1.311276	-2.379422	0.674845
H	1.007290	-2.526706	2.388698
H	3.540190	0.657344	1.647617
H	3.485588	-0.242367	3.175090
H	3.291970	-2.964734	2.888582
H	3.311399	-3.560853	1.220503
H	5.056397	-1.573964	1.900826
H	4.121562	-1.365269	0.403798
H	0.436070	1.375772	6.016313
H	0.852569	3.067178	6.396507
H	-0.469054	2.701977	5.258687
H	1.037010	4.683815	3.281655
H	2.295364	4.873466	4.526530
H	2.740259	4.360617	2.882487
H	3.409298	0.840064	5.206343
H	4.214900	1.892279	4.012602
H	3.775473	2.527611	5.616641
C	3.644526	-0.479352	-2.862596
C	3.842634	0.732236	-1.950943
C	2.410730	1.094843	-1.597017
O	1.715384	-0.157369	-1.539024
C	2.442726	-1.178768	-2.228731
H	3.404561	-0.154386	-3.887355
H	4.524825	-1.135113	-2.917252
H	4.392163	0.461352	-1.036566
H	4.380955	1.561252	-2.430702
H	1.942631	1.733510	-2.366955
H	2.327347	1.589269	-0.618844
H	2.753034	-1.944748	-1.497169
H	1.779272	-1.655902	-2.966074
C	5.563232	4.108063	1.806964
C	4.720706	5.021010	0.905847
C	3.741103	4.046180	0.220012
O	4.125405	2.736585	0.607497
C	5.452326	2.769815	1.088275
H	6.600958	4.451430	1.923423
H	5.118108	4.027646	2.810837
H	4.198609	5.805339	1.471867
H	5.353797	5.523450	0.158969
H	3.781894	4.143879	-0.880819
H	2.695583	4.194920	0.532559
H	5.603335	1.894546	1.737618
H	6.175222	2.704734	0.249532
C	-2.556615	-4.106888	3.770197
C	-3.021556	-3.159376	2.649720
C	-1.820499	-3.119165	1.678919
O	-0.896747	-4.095027	2.137275
C	-1.048807	-4.186980	3.534576
H	-2.802926	-3.737709	4.776524
H	-3.010426	-5.103145	3.652841
H	-3.937126	-3.526832	2.163408
H	-3.236450	-2.152572	3.036082
H	-1.355712	-2.114708	1.673878
H	-2.094070	-3.382520	0.645191
H	-0.593650	-5.130605	3.871043
H	-0.532658	-3.350567	4.046356

Energy 0.000000 (Hartree)

Phenate

Atom	X	Y	Z (Angstrom)
C	-2.287022	-1.627739	0.000054
C	-0.900644	-1.640237	0.000527
C	-0.099289	-0.429252	0.000088
C	-0.900535	0.781814	-0.001020
C	-2.286908	0.769454	-0.001481
C	-3.017516	-0.429112	-0.000939
H	-2.827404	-2.584680	0.000467
H	-0.348862	-2.587893	0.001287
H	-0.348618	1.729391	-0.001472
H	-2.827217	1.726435	-0.002297
H	-4.112024	-0.429051	-0.001307
O	1.152725	-0.429299	0.000373

Energy -306.580094 (Hartree)

Water

Atom	X	Y	Z (Angstrom)
O	0.014514	-0.130232	0.000000
H	0.974984	-0.079128	0.000000
H	-0.257928	0.792210	0.000000

Energy -76.337816 (Hartree)

Ph-Cluster-1.1-COMPLAX-Best

Atom	X	Y	Z (Angstrom)
C	-2.287022	-1.627739	0.000054
C	-0.900644	-1.640237	0.000527
C	-0.099289	-0.429252	0.000088
C	-0.900535	0.781814	-0.001020
C	-2.286908	0.769454	-0.001481
C	-3.017516	-0.429112	-0.000939
H	-2.827404	-2.584680	0.000467
H	-0.348862	-2.587893	0.001287
H	-0.348618	1.729391	-0.001472
H	-2.827217	1.726435	-0.002297
H	-4.112024	-0.429051	-0.001307
O	1.152725	-0.429299	0.000373
C	3.112500	1.414514	-1.881280
H	3.977378	0.824917	-1.544700
H	3.477362	2.315347	-2.374975
H	2.543837	0.805209	-2.601355
O	2.333561	1.833220	-0.784666
H	1.908101	1.053208	-0.413507

Energy -27.981411 (Hartree)

Ph-Cluster-1.1-COMPLAX-DFT

Atom	X	Y	Z (Angstrom)
C	-2.183134	-1.575744	0.009076
C	-0.804159	-1.659718	0.149564
C	0.047061	-0.505844	0.067990
C	-0.642987	0.734874	-0.163263
C	-2.022575	0.796110	-0.301410
C	-2.823165	-0.351256	-0.219926
H	-2.782223	-2.492792	0.079518
H	-0.313892	-2.622793	0.327439
H	-0.027504	1.638626	-0.221664
H	-2.494218	1.771362	-0.477374
H	-3.910206	-0.292564	-0.329829
O	1.312372	-0.587241	0.193103
C	2.618462	1.484475	-1.756368
H	3.235154	0.621621	-2.092607

H	3.079621	2.392604	-2.191090
H	1.625910	1.364575	-2.242806
O	2.544479	1.610767	-0.381611
H	2.090431	0.769188	-0.054943

Energy -422.218262 (Hartree)

Ph-Cluster-1.2-COMPLAX-Best

Atom	X	Y	Z (Angstrom)
C	-2.287022	-1.627739	0.000054
C	-0.900644	-1.640237	0.000527
C	-0.099289	-0.429252	0.000088
C	-0.900535	0.781814	-0.001020
C	-2.286908	0.769454	-0.001481
C	-3.017516	-0.429112	-0.000939
H	-2.827404	-2.584680	0.000467
H	-0.348862	-2.587893	0.001287
H	-0.348618	1.729391	-0.001472
H	-2.827217	1.726435	-0.002297
H	-4.112024	-0.429051	-0.001307
O	1.152725	-0.429299	0.000373
C	3.165497	-2.675679	-1.403232
H	3.424729	-3.674774	-1.781225
H	4.079180	-2.208090	-1.018598
H	2.774345	-2.069631	-2.235698
O	2.224881	-2.857421	-0.370513
H	1.850024	-1.996810	-0.155899

Energy -27.981164 (Hartree)

Ph-Cluster-1.2-COMPLAX-DFT

Atom	X	Y	Z (Angstrom)
C	-2.042356	-1.693689	-0.210390
C	-0.670243	-1.662983	-0.003517
C	0.048349	-0.429075	0.168931
C	-0.766642	0.752390	0.116287
C	-2.138570	0.698907	-0.091169
C	-2.806931	-0.520358	-0.259900
H	-2.536809	-2.665388	-0.336337
H	-0.084106	-2.586849	0.039916
H	-0.253268	1.711121	0.246025
H	-2.709172	1.636028	-0.123374
H	-3.888272	-0.555422	-0.423150
O	1.307584	-0.375461	0.355158
C	2.639950	-2.626012	-1.364067
H	3.096644	-3.575441	-1.705256
H	3.296280	-1.806286	-1.731622
H	1.676524	-2.519789	-1.908807
O	2.494286	-2.645412	0.010623
H	2.052093	-1.768853	0.249764

Energy -422.218260 (Hartree)

Ph-Cluster-1.3-COMPLAX-Best

Atom	X	Y	Z (Angstrom)
C	-2.287022	-1.627739	0.000054
C	-0.900644	-1.640237	0.000527
C	-0.099289	-0.429252	0.000088
C	-0.900535	0.781814	-0.001020
C	-2.286908	0.769454	-0.001481
C	-3.017516	-0.429112	-0.000939
H	-2.827404	-2.584680	0.000467
H	-0.348862	-2.587893	0.001287
H	-0.348618	1.729391	-0.001472
H	-2.827217	1.726435	-0.002297
H	-4.112024	-0.429051	-0.001307

O	1.152725	-0.429299	0.000373
C	2.505428	-2.158571	-2.406848
H	3.199007	-2.828396	-2.934829
H	2.637981	-1.145904	-2.803846
H	1.470656	-2.488319	-2.605586
O	2.842181	-2.217073	-1.040926
H	2.267709	-1.602828	-0.572292

Energy -27.980945 (Hartree)

Ph-Cluster-1.3-COMPLAX-DFT

Atom	X	Y	Z (Angstrom)
C	-2.081562	-1.653216	-0.164496
C	-0.699304	-1.543790	-0.224146
C	-0.029032	-0.274633	-0.130474
C	-0.901614	0.854051	0.034739
C	-2.282723	0.721941	0.092671
C	-2.903816	-0.529462	-0.006157
H	-2.537563	-2.648467	-0.242322
H	-0.066270	-2.429538	-0.341091
H	-0.425932	1.837554	0.112099
H	-2.898804	1.621598	0.218747
H	-3.992703	-0.626216	0.040096
O	1.237598	-0.149071	-0.187216
C	2.330258	-2.247267	-2.237461
H	2.768260	-3.150340	-2.705441
H	2.853440	-1.371846	-2.682071
H	1.275305	-2.186413	-2.583514
O	2.453711	-2.327210	-0.862853
H	2.020402	-1.488934	-0.501159

Energy -422.218263 (Hartree)

Ph-Cluster-2.1-COMPLAX-Best

Atom	X	Y	Z (Angstrom)
C	-2.287022	-1.627739	0.000054
C	-0.900644	-1.640237	0.000527
C	-0.099289	-0.429252	0.000088
C	-0.900535	0.781814	-0.001020
C	-2.286908	0.769454	-0.001481
C	-3.017516	-0.429112	-0.000939
H	-2.827404	-2.584680	0.000467
H	-0.348862	-2.587893	0.001287
H	-0.348618	1.729391	-0.001472
H	-2.827217	1.726435	-0.002297
H	-4.112024	-0.429051	-0.001307
O	1.152725	-0.429299	0.000373
C	3.767226	-1.798205	1.351878
H	4.691085	-2.385748	1.443868
H	3.182885	-1.934223	2.269422
H	4.024074	-0.733450	1.243917
O	3.070048	-2.305217	0.235101
H	2.324839	-1.717408	0.073545
C	2.650641	2.398599	-0.937248
H	3.283140	3.293497	-0.847083
H	1.657274	2.715857	-1.277833
H	3.088806	1.724577	-1.691886
O	2.585842	1.815180	0.343321
H	2.052583	1.015969	0.277516

Energy -36.226953 (Hartree)

Ph-Cluster-2.1-COMPLAX-DFT

Atom	X	Y	Z (Angstrom)
C	-1.978673	-1.818878	0.216334
C	-0.677084	-1.454955	-0.103822

C	-0.286664	-0.085074	-0.218162
C	-1.314549	0.874200	0.026708
C	-2.611774	0.491208	0.346354
C	-2.969870	-0.857518	0.445820
H	-2.229288	-2.883716	0.292763
H	0.096690	-2.210493	-0.274093
H	-1.043683	1.931990	-0.048827
H	-3.367184	1.266039	0.524312
H	-3.992813	-1.151692	0.697948
O	0.915342	0.258087	-0.525511
C	3.016050	-1.408788	1.020002
H	3.953314	-1.925326	1.297981
H	2.208779	-1.840849	1.650362
H	3.114265	-0.344424	1.307557
O	2.784915	-1.569441	-0.344290
H	2.055051	-0.924738	-0.556103
C	3.615095	1.815984	-0.700061
H	4.577256	2.280185	-0.414897
H	3.312203	2.272776	-1.667717
H	3.803351	0.742381	-0.895228
O	2.688440	2.021795	0.316488
H	1.905962	1.460509	0.084878

Energy -537.851905 (Hartree)

Ph-Cluster-2.2-COMPLAX-Best

Atom	X	Y	Z (Angstrom)
C	-2.287022	-1.627739	0.000054
C	-0.900644	-1.640237	0.000527
C	-0.099289	-0.429252	0.000088
C	-0.900535	0.781814	-0.001020
C	-2.286908	0.769454	-0.001481
C	-3.017516	-0.429112	-0.000939
H	-2.827404	-2.584680	0.000467
H	-0.348862	-2.587893	0.001287
H	-0.348618	1.729391	-0.001472
H	-2.827217	1.726435	-0.002297
H	-4.112024	-0.429051	-0.001307
O	1.152725	-0.429299	0.000373
C	2.753513	2.382375	-0.750886
H	3.202951	3.372839	-0.592936
H	1.839672	2.512416	-1.342233
H	3.461991	1.755774	-1.319307
O	2.463904	1.858783	0.524566
H	1.994964	1.026527	0.403535
C	3.995705	-1.676334	-0.958826
H	4.805421	-2.393263	-1.152040
H	4.359514	-0.929338	-0.245776
H	3.733849	-1.173203	-1.904942
O	2.919536	-2.409896	-0.419000
H	2.216754	-1.782646	-0.220751

Energy -36.226763 (Hartree)

Ph-Cluster-2.2-COMPLAX-DFT

Atom	X	Y	Z (Angstrom)
C	-1.755459	-1.457195	-0.600896
C	-0.437310	-1.454137	-0.163853
C	0.132414	-0.317522	0.486749
C	-0.731935	0.806279	0.647432
C	-2.048414	0.785416	0.200718
C	-2.585295	-0.342911	-0.427508
H	-2.149255	-2.352657	-1.096448
H	0.216105	-2.319335	-0.313193
H	-0.318669	1.692169	1.140368

H	-2.674956	1.673434	0.346868
H	-3.622202	-0.353391	-0.775761
O	1.353382	-0.307584	0.901647
C	2.346123	2.056996	-1.008232
H	2.884239	2.931736	-1.417979
H	1.266902	2.313673	-0.989919
H	2.459395	1.234341	-1.746063
O	2.860837	1.748615	0.243339
H	2.310523	1.001379	0.601248
C	3.448816	-1.640807	-0.932700
H	4.386742	-2.153987	-1.218379
H	3.701872	-0.595687	-0.669788
H	2.818354	-1.595598	-1.848885
O	2.840213	-2.317349	0.117966
H	2.242041	-1.642013	0.538953

Energy -537.851517 (Hartree)

Ph-Cluster-2.3-COMPLAX-Best

Atom	X	Y	Z (Angstrom)
C	-2.287022	-1.627739	0.000054
C	-0.900644	-1.640237	0.000527
C	-0.099289	-0.429252	0.000088
C	-0.900535	0.781814	-0.001020
C	-2.286908	0.769454	-0.001481
C	-3.017516	-0.429112	-0.000939
H	-2.827404	-2.584680	0.000467
H	-0.348862	-2.587893	0.001287
H	-0.348618	1.729391	-0.001472
H	-2.827217	1.726435	-0.002297
H	-4.112024	-0.429051	-0.001307
O	1.152725	-0.429299	0.000373
C	3.687802	-1.938032	1.640490
H	4.155865	-2.457054	2.488508
H	3.985600	-0.886024	1.674266
H	4.052318	-2.386486	0.701600
O	2.292794	-2.078792	1.801565
H	1.860996	-1.544989	1.126668
C	2.000627	1.674658	2.357990
H	2.577468	2.434060	2.904722
H	2.116208	0.716932	2.876978
H	0.934848	1.961219	2.366781
O	2.536001	1.615911	1.055405
H	2.067445	0.924093	0.577153

Energy -36.226468 (Hartree)

Ph-Cluster-2.3-COMPLAX-DFT

Atom	X	Y	Z (Angstrom)
C	-2.455178	-1.568658	-0.056992
C	-1.092121	-1.692474	0.184839
C	-0.230807	-0.555439	0.229949
C	-0.862253	0.709300	0.020759
C	-2.226185	0.813573	-0.219933
C	-3.047923	-0.318678	-0.265210
H	-3.075406	-2.472706	-0.083410
H	-0.637408	-2.674831	0.346683
H	-0.225650	1.598908	0.063522
H	-2.664009	1.807075	-0.374152
H	-4.121442	-0.228807	-0.454984
O	1.034585	-0.658632	0.442133
C	3.811814	-1.652024	1.466892
H	4.649847	-1.894550	2.146254
H	3.872681	-0.572399	1.228481
H	3.996979	-2.203947	0.519222

O	2.611629	-1.999187	2.077683
H	1.898474	-1.614411	1.507969
C	2.058637	1.459604	2.450244
H	2.718914	2.158928	2.995796
H	2.210438	0.447581	2.871983
H	1.012343	1.753986	2.683314
O	2.348403	1.514567	1.088886
H	1.878296	0.728552	0.696475

Energy -537.851900 (Hartree)

Ph-Cluster-3.1-COMPLAX-Best

Atom	X	Y	Z (Angstrom)
C	-2.287022	-1.627739	0.000054
C	-0.900644	-1.640237	0.000527
C	-0.099289	-0.429252	0.000088
C	-0.900535	0.781814	-0.001020
C	-2.286908	0.769454	-0.001481
C	-3.017516	-0.429112	-0.000939
H	-2.827404	-2.584680	0.000467
H	-0.348862	-2.587893	0.001287
H	-0.348618	1.729391	-0.001472
H	-2.827217	1.726435	-0.002297
H	-4.112024	-0.429051	-0.001307
O	1.152725	-0.429299	0.000373
C	3.561616	1.716898	1.019837
H	3.948873	2.668608	1.411699
H	4.265451	1.348982	0.262933
H	3.501414	0.990617	1.842605
O	2.302314	1.996826	0.446905
H	1.882341	1.158948	0.224645
C	3.910513	-2.118277	-0.787966
H	4.594153	-2.977870	-0.832124
H	4.225530	-1.470193	0.034977
H	3.981073	-1.558412	-1.735492
O	2.617821	-2.637354	-0.560833
H	2.027028	-1.890159	-0.414164
C	1.667902	-2.368398	2.726931
H	0.568375	-2.337987	2.727890
H	2.011477	-2.645895	3.722029
H	1.989057	-3.131018	2.000498
O	2.206478	-1.091341	2.449229
H	1.872793	-0.818625	1.587267

Energy -44.471004 (Hartree)

Ph-Cluster-3.1-COMPLAX-DFT

Atom	X	Y	Z (Angstrom)
C	-2.153463	-1.555901	0.046018
C	-0.770294	-1.608673	-0.091572
C	0.021245	-0.430758	-0.049358
C	-0.670407	0.796764	0.128511
C	-2.053465	0.832247	0.263564
C	-2.816536	-0.339036	0.227249
H	-2.728796	-2.487919	0.009699
H	-0.257030	-2.564177	-0.236714
H	-0.069962	1.710856	0.144574
H	-2.550337	1.799545	0.399752
H	-3.904376	-0.303721	0.335528
O	1.323188	-0.473657	-0.165738
C	2.966965	2.016684	1.052863
H	3.134648	3.069682	1.343459
H	3.949309	1.501643	1.113167
H	2.327809	1.551516	1.827952
O	2.402341	1.975427	-0.218415

H	2.039479	1.061600	-0.331763
C	3.829842	-2.522627	-0.545544
H	4.388892	-3.475844	-0.541065
H	3.952771	-2.055669	0.450471
H	4.331750	-1.859214	-1.282754
O	2.497190	-2.775702	-0.858045
H	2.001293	-1.935932	-0.687302
C	1.790636	-2.319779	2.487118
H	0.713958	-2.191896	2.722775
H	2.265913	-2.791090	3.365713
H	1.858900	-3.029174	1.640320
O	2.434115	-1.111924	2.216197
H	2.075317	-0.798087	1.354486

Energy -653.479879 (Hartree)

Ph-Cluster-3.2-COMPLAX-Best

Atom	X	Y	Z (Angstrom)
C	-2.287022	-1.627739	0.000054
C	-0.900644	-1.640237	0.000527
C	-0.099289	-0.429252	0.000088
C	-0.900535	0.781814	-0.001020
C	-2.286908	0.769454	-0.001481
C	-3.017516	-0.429112	-0.000939
H	-2.827404	-2.584680	0.000467
H	-0.348862	-2.587893	0.001287
H	-0.348618	1.729391	-0.001472
H	-2.827217	1.726435	-0.002297
H	-4.112024	-0.429051	-0.001307
O	1.152725	-0.429299	0.000373
C	1.254107	-1.502219	-3.123313
H	1.611937	-1.695520	-4.145033
H	0.276293	-1.012639	-3.189139
H	1.141780	-2.462010	-2.593939
O	2.204830	-0.656613	-2.514634
H	1.905349	-0.481068	-1.616244
C	3.713709	-2.628003	-0.114703
H	4.199484	-3.615466	-0.144925
H	4.213206	-2.027488	0.652274
H	3.834894	-2.137362	-1.092952
O	2.361584	-2.852130	0.224484
H	1.904695	-2.003938	0.192932
C	3.559417	1.241124	1.609011
H	4.096309	2.096192	2.039903
H	4.227101	0.735939	0.902880
H	3.293140	0.542468	2.419285
O	2.428611	1.763245	0.948960
H	1.938509	1.023912	0.574989

Energy -44.470660 (Hartree)

Ph-Cluster-3.2-COMPLAX-DFT

Atom	X	Y	Z (Angstrom)
C	-2.408125	-1.080300	-0.135495
C	-1.076832	-1.262098	-0.494933
C	-0.105056	-0.250564	-0.276274
C	-0.565394	0.948086	0.334270
C	-1.898720	1.114090	0.690826
C	-2.841132	0.107086	0.460756
H	-3.125893	-1.886824	-0.323281
H	-0.745450	-2.197783	-0.954216
H	0.173681	1.733518	0.519218
H	-2.211014	2.053738	1.160366
H	-3.889219	0.243873	0.741796
O	1.145203	-0.407232	-0.614515

C	1.488209	-2.659123	-2.959015
H	1.975124	-3.208263	-3.785011
H	0.401271	-2.884705	-3.024392
H	1.858696	-3.086976	-2.007712
O	1.773672	-1.302982	-3.082725
H	1.529067	-0.880314	-2.225967
C	4.048128	-1.644221	-0.559002
H	4.944737	-2.276143	-0.435501
H	4.279815	-0.637566	-0.159870
H	3.853794	-1.526389	-1.641753
O	2.975113	-2.247835	0.108231
H	2.193489	-1.677552	-0.073133
C	2.675937	0.649189	1.959014
H	3.395829	1.130975	2.644345
H	2.930772	-0.426171	1.898649
H	1.674300	0.720700	2.433564
O	2.733794	1.283084	0.718054
H	2.177844	0.736958	0.107823

Energy -653.482167 (Hartree)

Ph-Cluster-3.3-COMPLAX-Best

Atom	X	Y	Z (Angstrom)
C	-2.287022	-1.627739	0.000054
C	-0.900644	-1.640237	0.000527
C	-0.099289	-0.429252	0.000088
C	-0.900535	0.781814	-0.001020
C	-2.286908	0.769454	-0.001481
C	-3.017516	-0.429112	-0.000939
H	-2.827404	-2.584680	0.000467
H	-0.348862	-2.587893	0.001287
H	-0.348618	1.729391	-0.001472
H	-2.827217	1.726435	-0.002297
H	-4.112024	-0.429051	-0.001307
O	1.152725	-0.429299	0.000373
C	1.506448	0.225830	-3.234763
H	1.897197	0.181240	-4.264595
H	1.685686	1.231865	-2.845447
H	0.420778	0.031025	-3.254579
O	2.216632	-0.738512	-2.488445
H	1.894088	-0.694418	-1.582970
C	3.142292	-3.130535	-0.093652
H	3.956075	-3.802141	0.217919
H	3.487874	-2.546516	-0.951229
H	2.276599	-3.742332	-0.399963
O	2.854534	-2.301707	1.011564
H	2.215267	-1.640753	0.726385
C	3.664383	1.862114	0.139263
H	4.174610	2.802224	-0.114932
H	4.198979	1.040828	-0.347703
H	3.700076	1.718836	1.231241
O	2.346026	1.973899	-0.349996
H	1.896574	1.138747	-0.185301

Energy -44.470581 (Hartree)

Ph-Cluster-3.3-COMPLAX-DFT

Atom	X	Y	Z (Angstrom)
C	-2.184841	-1.730918	0.722441
C	-0.812583	-1.541332	0.608011
C	-0.275938	-0.412176	-0.067276
C	-1.211418	0.507558	-0.608756
C	-2.581961	0.303886	-0.487351
C	-3.090602	-0.815404	0.177386
H	-2.558363	-2.615243	1.251026

H	-0.101991	-2.253038	1.039336
H	-0.818759	1.387131	-1.127765
H	-3.271132	1.037191	-0.921214
H	-4.169314	-0.970287	0.269950
O	1.010630	-0.234950	-0.183861
C	1.891268	0.932431	-3.103046
H	2.533602	1.117153	-3.982816
H	2.128446	1.698957	-2.341362
H	0.842405	1.102188	-3.430078
O	2.112726	-0.362911	-2.640921
H	1.698360	-0.414873	-1.749238
C	2.919340	-2.769261	-0.160903
H	3.711674	-3.494776	0.096401
H	3.295639	-2.138044	-0.989092
H	2.065576	-3.350614	-0.569122
O	2.584078	-2.035924	0.974990
H	1.981490	-1.315171	0.658922
C	3.846754	1.097973	-0.049002
H	4.705406	1.715213	0.270666
H	4.047069	0.753860	-1.084212
H	3.820971	0.192771	0.586541
O	2.677390	1.855687	0.050661
H	1.934881	1.212449	-0.003236

Energy -653.480857 (Hartree)

Ph-Cluster-ABCluster-Best

Atom	X	Y	Z (Angstrom)
C	4.526195	4.979826	-3.160336
C	4.794285	4.487528	-1.902949
C	5.935082	4.900558	-1.184846
C	6.789116	5.826344	-1.819629
C	6.509286	6.307378	-3.079015
C	5.375063	5.896314	-3.762665
H	3.637777	4.646067	-3.686295
H	4.132609	3.768266	-1.438812
H	7.677186	6.151829	-1.292962
H	7.185820	7.018715	-3.541613
H	5.157332	6.282504	-4.750926
O	6.185286	4.450112	0.008058
C	5.620133	2.309055	2.303358
H	5.256745	1.428527	2.841510
H	5.188021	3.207731	2.758553
H	6.711028	2.362013	2.415990
O	5.243984	2.173939	0.964687
H	5.545284	3.009090	0.505062
C	9.182701	3.281258	0.163650
H	10.228723	3.160812	0.461440
H	9.135294	3.330104	-0.933526
H	8.622165	2.394351	0.486759
O	8.700760	4.446217	0.755470
H	7.730199	4.520691	0.495483
C	6.522469	6.996564	1.952358
H	6.414446	7.676851	2.801501
H	6.448864	7.580818	1.024834
H	7.513767	6.532067	1.989640
O	5.502077	6.044112	2.040731
H	5.664203	5.393939	1.309770

Energy 0.000000 (Hartree)

Ph-Cluster-ABCluster-DFT

Atom	X	Y	Z (Angstrom)
C	4.421886	5.234835	-3.301566
C	4.601687	4.839359	-1.980461

C	5.851795	4.991441	-1.326432
C	6.903870	5.565662	-2.089902
C	6.709206	5.955113	-3.410306
C	5.469288	5.797483	-4.036737
H	3.440553	5.100677	-3.770554
H	3.780484	4.397980	-1.408268
H	7.872936	5.688048	-1.596564
H	7.546298	6.393551	-3.965284
H	5.322351	6.106509	-5.075547
O	6.028872	4.624096	-0.087158
C	6.156901	2.659140	2.391386
H	5.907130	1.859059	3.111138
H	5.564243	3.555239	2.650830
H	7.223826	2.923890	2.545672
O	5.897172	2.206273	1.095278
H	5.868633	3.010286	0.530163
C	8.973347	3.333659	0.141309
H	9.941338	3.041297	0.586346
H	9.113681	3.345336	-0.961178
H	8.237491	2.539148	0.365858
O	8.588183	4.573791	0.650106
H	7.631691	4.681790	0.414680
C	6.200607	6.558973	2.408175
H	6.079130	6.979485	3.422354
H	6.430377	7.406982	1.729156
H	7.095671	5.907612	2.417415
O	5.034923	5.881806	2.056255
H	5.242330	5.405060	1.219115

Energy 0.000000 (Hartree)

Ph-Cluster-OGOLEM-Best

Atom	X	Y	Z (Angstrom)
C	2.680532	2.651754	1.180673
C	1.485210	2.465251	0.543823
C	1.064805	1.154210	0.120340
C	1.974638	0.068299	0.385549
C	3.167211	0.288194	1.017797
C	3.529660	1.572430	1.425742
H	2.972862	3.640677	1.503388
H	0.809523	3.286266	0.358073
H	1.688985	-0.918094	0.050902
H	3.838548	-0.537294	1.208749
H	4.469193	1.730542	1.933453
O	-0.027957	0.975887	-0.470078
C	-1.257156	1.286331	2.138842
H	-1.339616	2.334260	2.427975
H	-2.039249	0.702107	2.629793
H	-1.396642	1.212277	1.046818
O	0.021540	0.845367	2.492666
H	0.010741	-0.137416	2.459818
C	-2.083152	-1.767004	-0.948947
H	-2.336284	-2.824272	-0.872326
H	-2.192463	-1.450062	-1.989342
H	-2.773387	-1.188110	-0.327098
O	-0.754232	-1.620476	-0.511131
H	-0.505307	-0.664687	-0.592825
C	0.576381	-2.812795	2.494905
H	0.725386	-2.772671	3.573534
H	1.532725	-2.621263	1.997512
H	0.227924	-3.812864	2.221340
O	-0.378736	-1.834332	2.170914
H	-0.543678	-1.859884	1.198957

Energy -115790.568100 (Hartree)

Ph-Cluster-OGOLEM-DFT

Atom	X	Y	Z (Angstrom)
C	3.409774	2.605622	0.546381
C	2.336515	1.974044	1.161680
C	1.848166	0.710531	0.712536
C	2.537667	0.142282	-0.402481
C	3.609327	0.788822	-1.006738
C	4.065457	2.028686	-0.547491
H	3.746560	3.576855	0.927956
H	1.826872	2.430940	2.015539
H	2.194600	-0.832596	-0.764524
H	4.105269	0.311254	-1.860124
H	4.909508	2.531845	-1.027573
O	0.849214	0.129382	1.280674
C	-2.419104	0.645395	2.331854
H	-2.489751	1.591276	2.898597
H	-2.953330	0.814299	1.370785
H	-1.351996	0.485927	2.088821
O	-2.984003	-0.388535	3.085596
H	-2.536677	-1.215797	2.816444
C	-1.015630	-1.326493	-0.801153
H	-1.496344	-2.143177	-1.368464
H	-0.442153	-0.713878	-1.524062
H	-1.822850	-0.683375	-0.397563
O	-0.196429	-1.862207	0.189656
H	0.300125	-1.066727	0.634120
C	-0.339876	-2.145707	3.408922
H	-0.808724	-1.762546	4.330070
H	0.322780	-1.357662	3.010473
H	0.278022	-3.024749	3.679498
O	-1.355605	-2.482165	2.494774
H	-0.979380	-2.368918	1.585612

Energy 0.000000 (Hartree)

Ph-Cluster-CREST-Best

Atom	X	Y	Z (Angstrom)
C	1.431641	-1.922673	0.104075
C	0.088000	-1.946435	-0.141441
C	-0.638793	-0.725424	-0.379743
C	0.114722	0.505529	-0.351660
C	1.462547	0.494122	-0.114184
C	2.129934	-0.709029	0.120604
H	1.963175	-2.844019	0.288917
H	-0.473657	-2.868135	-0.158684
H	-0.421131	1.423687	-0.537058
H	2.014580	1.421491	-0.097516
H	3.190952	-0.705781	0.316681
O	-1.875427	-0.734919	-0.609234
C	-4.460773	1.124574	0.894802
H	-4.818634	1.956659	1.500138
H	-4.556812	0.195219	1.462897
H	-5.066107	1.052837	-0.012699
O	-3.111283	1.393803	0.582205
H	-2.763297	0.643187	0.052757
C	-2.673716	1.309674	-3.069630
H	-3.147486	1.787949	-3.926569
H	-1.601634	1.200860	-3.268969
H	-2.814124	1.938679	-2.184785
O	-3.294921	0.054724	-2.912617
H	-2.898175	-0.388491	-2.141654
C	-0.390614	2.062645	2.861829

H	0.566581	1.792053	3.305526
H	-1.066122	2.407462	3.651450
H	-0.240255	2.874595	2.144576
O	-0.891691	0.907044	2.230885
H	-1.734443	1.137897	1.791546

Energy -44.117324 (Hartree)

Ph-Cluster-Crest-DFT

Atom	X	Y	Z (Angstrom)
C	1.586189	-1.485432	-1.341452
C	0.200480	-1.372567	-1.342084
C	-0.500224	-0.787626	-0.249508
C	0.297639	-0.321104	0.835480
C	1.682443	-0.440417	0.816503
C	2.350619	-1.024629	-0.265147
H	2.084048	-1.942088	-2.204958
H	-0.393957	-1.725457	-2.190720
H	-0.209099	0.147468	1.686095
H	2.256685	-0.064526	1.671046
H	3.440677	-1.114532	-0.270501
O	-1.790573	-0.686266	-0.244569
C	-4.341749	0.807596	0.860206
H	-4.989051	1.685821	1.031650
H	-4.634662	0.032340	1.598181
H	-4.559040	0.409721	-0.147945
O	-3.002689	1.183032	0.970370
H	-2.453076	0.441638	0.541732
C	-2.851572	1.061715	-2.564213
H	-3.532118	1.572917	-3.269098
H	-1.837561	1.082636	-3.017584
H	-2.801521	1.669952	-1.639900
O	-3.317801	-0.229424	-2.330088
H	-2.753275	-0.577008	-1.592249
C	-0.748198	2.995081	2.315770
H	-0.036578	3.472448	3.011999
H	-1.402417	3.795397	1.913008
H	-0.158995	2.598285	1.464936
O	-1.453012	2.006864	2.997238
H	-2.108572	1.647947	2.358246

Energy 0.000000 (Hartree)

Ph-Cluster-SOLVATOR-Best

Atom	X	Y	Z (Angstrom)
C	-2.287022	-1.627739	0.000054
C	-0.900644	-1.640237	0.000527
C	-0.099289	-0.429252	0.000088
C	-0.900535	0.781814	-0.001020
C	-2.286908	0.769454	-0.001481
C	-3.017516	-0.429112	-0.000939
H	-2.827404	-2.584680	0.000467
H	-0.348862	-2.587893	0.001287
H	-0.348618	1.729391	-0.001472
H	-2.827217	1.726435	-0.002297
H	-4.112024	-0.429051	-0.001307
O	1.152725	-0.429299	0.000373
C	2.550583	2.717012	-0.792801
O	2.127729	2.210480	0.452807
H	2.698303	3.787878	-0.659485
H	3.493660	2.262408	-1.112008
H	1.796343	2.554232	-1.570336
H	1.959788	1.252369	0.362415
C	2.156676	-2.254004	-2.423669
O	2.091250	-2.786419	-1.122050

H	2.081724	-3.094343	-3.111601
H	1.332074	-1.560900	-2.617370
H	3.104682	-1.732003	-2.592612
H	1.983846	-2.040412	-0.501931
C	-6.596654	-0.064901	-1.502767
O	-6.277803	-0.686353	-0.273581
H	-5.845701	-0.388867	-2.221518
H	-7.582037	-0.370272	-1.859415
H	-6.562853	1.025370	-1.415115
H	-6.958097	-0.473596	0.377893

Energy 0.000000 (Hartree)

Ph-Cluster-SOLVATOR-DFT

Atom	X	Y	Z (Angstrom)
C	-2.666751	-1.783265	0.918921
C	-1.357408	-1.732278	0.462233
C	-0.587617	-0.527582	0.528758
C	-1.266349	0.618040	1.051721
C	-2.582864	0.542833	1.500972
C	-3.304515	-0.652680	1.447237
H	-3.215682	-2.729703	0.850339
H	-0.873029	-2.608815	0.022677
H	-0.702808	1.555387	1.091346
H	-3.059204	1.446961	1.896814
H	-4.337687	-0.703182	1.801563
O	0.631417	-0.472328	0.122346
C	1.068054	2.234835	-1.469044
O	1.467457	2.016112	-0.155238
H	1.113811	3.317170	-1.688914
H	1.718968	1.728700	-2.214690
H	0.032255	1.902273	-1.677904
H	1.252784	1.065588	0.039140
C	0.462481	-1.645770	-2.765326
O	1.040600	-2.322428	-1.697673
H	0.011396	-2.368619	-3.471364
H	-0.335092	-0.945809	-2.450587
H	1.193522	-1.044182	-3.349662
H	1.024148	-1.682713	-0.937984
C	-3.211133	-0.188425	-2.273235
O	-2.361366	0.880441	-1.977494
H	-3.618141	-0.032010	-3.286100
H	-2.691163	-1.164921	-2.265474
H	-4.062537	-0.261976	-1.569882
H	-2.033347	0.765856	-1.072364

Energy -653.471731 (Hartree)

Pyridine

Atom	X	Y	Z (Angstrom)
C	-2.913610	-1.069702	0.000010
C	-1.518495	-1.076508	-0.000064
C	-0.850662	0.145610	-0.000278
C	-1.605744	1.315842	-0.000396
C	-2.996690	1.207448	-0.000283
N	-3.642956	0.043785	-0.000092
H	0.241818	0.185471	-0.000349
H	-3.468655	-2.015183	0.000185
H	-0.971565	-2.021979	0.000036
H	-1.129224	2.298687	-0.000571
H	-3.619215	2.109934	-0.000387

Energy -248.030593 (Hartree)

Methanol

Atom	X	Y	Z (Angstrom)
C	0.124261	0.392335	0.020167
H	0.493432	-0.643329	0.025507
H	0.480222	0.865045	-0.915425
H	-0.981166	0.348768	-0.020279
O	0.607823	1.022802	1.173229
H	0.291093	1.929765	1.177879

Energy -115.599014 (Hartree)

Py-Cluster-1.1-COMPLAX-Best

Atom	X	Y	Z (Angstrom)
C	-2.913610	-1.069702	0.000010
C	-1.518495	-1.076508	-0.000064
C	-0.850662	0.145610	-0.000278
C	-1.605744	1.315842	-0.000396
C	-2.996690	1.207448	-0.000283
N	-3.642956	0.043785	-0.000092
H	0.241818	0.185471	-0.000349
H	-3.468655	-2.015183	0.000185
H	-0.971565	-2.021979	0.000036
H	-1.129224	2.298687	-0.000571
H	-3.619215	2.109934	-0.000387
O	-6.029776	-0.243228	-0.634885
H	-5.478682	0.544835	-0.615773
H	-6.811446	0.019159	-0.139638

Energy 0.000000 (Hartree)

Py-Cluster-1.1-COMPLAX-DFT

Atom	X	Y	Z (Angstrom)
C	-2.749335	-1.317399	-0.199040
C	-1.387378	-1.111552	0.012852
C	-0.925297	0.197483	0.131893
C	-1.840817	1.242287	0.034205
C	-3.182520	0.931659	-0.177973
N	-3.627349	-0.320388	-0.291613
H	0.136040	0.399949	0.297629
H	-3.150271	-2.331822	-0.299362
H	-0.706528	-1.962547	0.081564
H	-1.525665	2.284335	0.118907
H	-3.949789	1.708422	-0.267854
O	-6.259153	0.733482	-0.612117
H	-5.514170	0.106236	-0.620475
H	-6.541268	0.714363	0.307223

Energy -324.384386 (Hartree)

Py-Cluster-1.2-COMPLAX-Best

Atom	X	Y	Z (Angstrom)
C	-2.913610	-1.069702	0.000010
C	-1.518495	-1.076508	-0.000064
C	-0.850662	0.145610	-0.000278
C	-1.605744	1.315842	-0.000396
C	-2.996690	1.207448	-0.000283
N	-3.642956	0.043785	-0.000092
H	0.241818	0.185471	-0.000349
H	-3.468655	-2.015183	0.000185
H	-0.971565	-2.021979	0.000036
H	-1.129224	2.298687	-0.000571
H	-3.619215	2.109934	-0.000387
O	-5.595850	0.127442	-0.622603
H	-5.436278	0.922873	-0.105940
H	-6.265161	-0.338953	-0.113070

Energy 0.000000 (Hartree)

Py-Cluster-1.2-COMPLAX-DFT

Atom	X	Y	Z (Angstrom)
C	-2.774291	-1.293391	-0.164028
C	-1.406805	-1.098930	0.020766
C	-0.929754	0.206600	0.117955
C	-1.836740	1.259317	0.026467
C	-3.185133	0.959782	-0.157666
N	-3.644308	-0.288755	-0.250645
H	0.136406	0.399980	0.262156
H	-3.186765	-2.304762	-0.246836
H	-0.733121	-1.955907	0.085640
H	-1.510142	2.299029	0.094906
H	-3.946059	1.743569	-0.240329
O	-6.274831	0.788079	-0.507460
H	-5.534794	0.155423	-0.531370
H	-6.527736	0.781550	0.420557

Energy -324.384380 (Hartree)

Py-Cluster-1.3-COMPLAX-Best

Atom	X	Y	Z (Angstrom)
C	-2.913610	-1.069702	0.000010
C	-1.518495	-1.076508	-0.000064
C	-0.850662	0.145610	-0.000278
C	-1.605744	1.315842	-0.000396
C	-2.996690	1.207448	-0.000283
N	-3.642956	0.043785	-0.000092
H	0.241818	0.185471	-0.000349
H	-3.468655	-2.015183	0.000185
H	-0.971565	-2.021979	0.000036
H	-1.129224	2.298687	-0.000571
H	-3.619215	2.109934	-0.000387
O	-6.556507	0.112790	-0.303471
H	-5.603425	0.234804	-0.346612
H	-6.674407	-0.452565	0.465683

Energy 0.000000 (Hartree)

Py-Cluster-1.3-COMPLAX-DFT

Atom	X	Y	Z (Angstrom)
C	-2.742435	-1.336008	-0.122516
C	-1.372121	-1.106473	-0.014439
C	-0.925187	0.210427	0.071100
C	-1.863492	1.239123	0.044873
C	-3.211804	0.905123	-0.065054
N	-3.642231	-0.354463	-0.146624
H	0.142024	0.431259	0.155925
H	-3.132455	-2.357131	-0.193718
H	-0.673330	-1.945473	0.001092
H	-1.561094	2.286517	0.107101
H	-3.997562	1.667926	-0.094699
O	-6.309254	0.652394	-0.247044
H	-5.557041	0.037606	-0.316651
H	-6.513298	0.631183	0.692670

Energy -324.384383 (Hartree)

Py-Cluster-2.1-COMPLAX-Best

Atom	X	Y	Z (Angstrom)
C	-2.913610	-1.069702	0.000010
C	-1.518495	-1.076508	-0.000064
C	-0.850662	0.145610	-0.000278
C	-1.605744	1.315842	-0.000396
C	-2.996690	1.207448	-0.000283
N	-3.642956	0.043785	-0.000092
H	0.241818	0.185471	-0.000349

H	-3.468655	-2.015183	0.000185
H	-0.971565	-2.021979	0.000036
H	-1.129224	2.298687	-0.000571
H	-3.619215	2.109934	-0.000387
O	-5.595850	0.127442	-0.622603
H	-5.436278	0.922873	-0.105940
H	-6.265161	-0.338953	-0.113070
O	-3.606743	0.900004	-2.578740
H	-4.078148	0.927273	-1.740798
H	-2.683674	0.846721	-2.313733

Energy 0.000000 (Hartree)

Py-Cluster-2.1-COMPLAX-DFT

Atom	X	Y	Z (Angstrom)
C	-2.825392	-1.211364	0.162183
C	-1.496261	-1.080417	0.555472
C	-0.817344	0.088095	0.214747
C	-1.493317	1.070704	-0.503177
C	-2.824535	0.849598	-0.853837
N	-3.474253	-0.270715	-0.524136
H	0.226833	0.229089	0.505997
H	-3.393600	-2.114411	0.410001
H	-1.006938	-1.879136	1.116656
H	-1.002052	2.001959	-0.792302
H	-3.403586	1.589019	-1.424171
O	-6.026242	-0.022226	-1.641594
H	-5.183254	-0.232527	-1.175964
H	-6.653589	0.144050	-0.930911
O	-5.081365	2.401840	-2.569242
H	-5.531464	1.578015	-2.287716
H	-4.837101	2.214137	-3.480625

Energy -400.746474 (Hartree)

Py-Cluster-2.2-COMPLAX-Best

Atom	X	Y	Z (Angstrom)
C	-2.913610	-1.069702	0.000010
C	-1.518495	-1.076508	-0.000064
C	-0.850662	0.145610	-0.000278
C	-1.605744	1.315842	-0.000396
C	-2.996690	1.207448	-0.000283
N	-3.642956	0.043785	-0.000092
H	0.241818	0.185471	-0.000349
H	-3.468655	-2.015183	0.000185
H	-0.971565	-2.021979	0.000036
H	-1.129224	2.298687	-0.000571
H	-3.619215	2.109934	-0.000387
O	-5.963563	-0.044183	-0.320816
H	-5.547652	0.590840	0.269843
H	-6.054377	-0.830624	0.225433
O	-4.569587	-0.050108	-1.935449
H	-4.915710	-0.721292	-1.339778
H	-4.631589	-0.466161	-2.800423

Energy 0.000000 (Hartree)

Py-Cluster-2.2-COMPLAX-DFT

Atom	X	Y	Z (Angstrom)
C	-3.241155	-0.632368	-0.925529
C	-1.894717	-0.955199	-0.759018
C	-1.075937	-0.074700	-0.057863
C	-1.631899	1.098708	0.449414
C	-2.986682	1.335501	0.233238
N	-3.772454	0.491583	-0.435941
H	-0.016297	-0.297595	0.091885

H	-3.928514	-1.290880	-1.474446
H	-1.503182	-1.885816	-1.174893
H	-1.029211	1.820439	1.004529
H	-3.461770	2.245268	0.615732
O	-6.494543	0.304567	-1.056679
H	-5.568013	0.540612	-0.819589
H	-6.880456	0.024805	-0.220487
O	-5.760277	-1.952273	-2.469119
H	-6.150808	-1.189151	-1.994178
H	-5.644746	-1.613759	-3.362283

Energy -400.746651 (Hartree)

Py-Cluster-2.3-COMPLAX-Best

Atom	X	Y	Z (Angstrom)
C	-2.913610	-1.069702	0.000010
C	-1.518495	-1.076508	-0.000064
C	-0.850662	0.145610	-0.000278
C	-1.605744	1.315842	-0.000396
C	-2.996690	1.207448	-0.000283
N	-3.642956	0.043785	-0.000092
H	0.241818	0.185471	-0.000349
H	-3.468655	-2.015183	0.000185
H	-0.971565	-2.021979	0.000036
H	-1.129224	2.298687	-0.000571
H	-3.619215	2.109934	-0.000387
O	-6.015238	-0.108779	-1.529920
H	-5.124636	-0.370457	-1.277993
H	-5.968746	0.851758	-1.548125
O	-3.386744	-0.545364	2.764834
H	-3.741442	-0.642084	1.876043
H	-4.174159	-0.483750	3.313745

Energy 0.000000 (Hartree)

Py-Cluster-2.3-COMPLAX-DFT

Atom	X	Y	Z (Angstrom)
C	-2.737413	-1.361769	-0.392770
C	-1.446833	-0.874275	-0.581016
C	-1.114030	0.353992	-0.012657
C	-2.081200	1.038496	0.717935
C	-3.347367	0.470443	0.852951
N	-3.664775	-0.706700	0.305578
H	-0.111428	0.771085	-0.138509
H	-3.038690	-2.323030	-0.822850
H	-0.720985	-1.447679	-1.161248
H	-1.865796	2.003419	1.181284
H	-4.144977	0.966275	1.423343
O	-6.373867	-1.050517	0.915782
H	-5.421420	-1.097289	0.668575
H	-6.813134	-0.813472	0.092815
O	-6.068650	1.241090	2.426992
H	-6.310439	0.439670	1.917388
H	-5.893187	0.892222	3.306294

Energy -400.746649 (Hartree)

Py-Cluster-ABCluster-Best

Atom	X	Y	Z (Angstrom)
C	0.276086	5.651544	2.066908
C	-0.881800	5.524753	1.314730
C	-1.636020	6.660056	1.064165
C	-1.204190	7.874868	1.574249
C	-0.032535	7.895877	2.313014
N	0.689141	6.813034	2.554635
H	-2.543838	6.601454	0.481027

H	0.902984	4.798547	2.289630
H	-1.182627	4.560630	0.936343
H	-1.761495	8.782601	1.402644
H	0.350554	8.817314	2.732408
O	2.913783	3.819309	2.962018
H	3.086299	4.723211	3.286995
H	3.748103	3.500217	2.616027
O	3.105975	6.432093	3.846994
H	2.875150	6.455891	4.777384
H	2.294059	6.683129	3.357671

Energy 0.000000 (Hartree)

Py-Cluster-ABCluster-DFT

Atom	X	Y	Z (Angstrom)
C	0.369058	5.676679	2.073915
C	-0.812162	5.496427	1.355252
C	-1.610903	6.605127	1.089658
C	-1.199602	7.854312	1.550950
C	-0.004586	7.936326	2.260727
N	0.760519	6.875256	2.516803
H	-2.543541	6.499249	0.529298
H	1.037815	4.836264	2.305982
H	-1.093669	4.498275	1.013760
H	-1.792504	8.752376	1.365831
H	0.353810	8.899396	2.639960
O	2.816663	3.779083	3.012122
H	3.023996	4.648646	3.413922
H	3.380916	3.762632	2.233002
O	3.122624	6.366139	3.926993
H	2.853344	6.401586	4.850305
H	2.337853	6.706757	3.438360

Energy 0.000000 (Hartree)

Py-Cluster-OGOLEM-Best

Atom	X	Y	Z (Angstrom)
C	0.389638	1.309874	-1.713170
C	-0.717274	2.012510	-1.265946
C	-1.724366	1.309158	-0.622943
C	-1.581700	-0.059288	-0.456758
C	-0.434545	-0.669269	-0.940955
N	0.529195	0.003358	-1.554454
H	-2.604216	1.820183	-0.259120
H	1.203964	1.809441	-2.222101
H	-0.788602	3.078387	-1.416986
H	-2.342425	-0.643038	0.037016
H	-0.273493	-1.734193	-0.840170
O	0.819809	-3.721339	-1.382350
H	1.464074	-3.106326	-1.781044
H	0.775975	-4.474035	-1.973475
O	2.504724	-1.757831	-2.365296
H	3.215505	-1.604203	-1.740645
H	1.829997	-1.069209	-2.185601

Energy -69082.945200 (Hartree)

Py-Cluster-OGOLEM-DFT

Atom	X	Y	Z (Angstrom)
C	0.369080	1.335073	-1.780340
C	-0.748238	1.995263	-1.275938
C	-1.712014	1.245951	-0.603603
C	-1.519057	-0.125823	-0.466660
C	-0.368124	-0.699016	-1.006094
N	0.555697	0.021796	-1.648702
H	-2.602937	1.727575	-0.192118

H	1.149088	1.887677	-2.315107
H	-0.856965	3.073574	-1.409090
H	-2.247002	-0.753151	0.051728
H	-0.170140	-1.777161	-0.930343
O	0.809216	-3.702350	-1.272855
H	1.517450	-3.135370	-1.643688
H	0.363471	-4.031464	-2.059421
O	2.553639	-1.825072	-2.301209
H	3.243579	-1.660800	-1.650570
H	1.929515	-1.072519	-2.179989

Energy 0.000000 (Hartree)

Py-Cluster-CREST-Best

Atom	X	Y	Z (Angstrom)
C	0.928685	-0.842163	0.007827
C	-0.436543	-0.994257	-0.174391
C	-1.215606	0.145850	-0.282200
C	-0.596551	1.382591	-0.199623
C	0.776457	1.419022	-0.015007
N	1.530150	0.334839	0.086771
H	-2.282335	0.072025	-0.429602
H	1.580403	-1.701399	0.095163
H	-0.875177	-1.977015	-0.233385
H	-1.162653	2.297017	-0.278266
H	1.306891	2.359545	0.054986
O	-0.200802	-0.188853	4.384175
H	0.093339	-1.051084	4.695843
H	0.437070	0.457351	4.704705
O	-0.210044	-0.763639	-3.778821
H	0.121765	-0.084378	-4.374500
H	-0.049575	-1.608271	-4.211971

Energy -26.327788 (Hartree)

Py-Cluster-CREST-DFT

Atom	X	Y	Z (Angstrom)
C	-1.037160	-1.464528	0.584975
C	-2.300714	-1.260195	0.037271
C	-2.505345	-0.129000	-0.750894
C	-1.441816	0.745140	-0.957303
C	-0.211612	0.456801	-0.367539
N	-0.018215	-0.627611	0.388974
H	-3.483259	0.067146	-1.198404
H	-0.834745	-2.341988	1.208575
H	-3.104674	-1.974388	0.226688
H	-1.554869	1.643505	-1.567494
H	0.660245	1.111775	-0.502426
O	2.632771	-0.409850	1.259118
H	2.540073	-0.083728	2.159923
H	1.713074	-0.648702	0.999567
O	2.747640	1.762469	-0.440476
H	2.832933	1.037079	0.212884
H	3.111148	1.373254	-1.241733

Energy 0.000000 (Hartree)

Py-Cluster-SOLVATOR-Best

Atom	X	Y	Z (Angstrom)
C	-2.913610	-1.069702	0.000010
C	-1.518495	-1.076508	-0.000064
C	-0.850662	0.145610	-0.000278
C	-1.605744	1.315842	-0.000396
C	-2.996690	1.207448	-0.000283
N	-3.642956	0.043785	-0.000092
H	0.241818	0.185471	-0.000349

H	-3.468655	-2.015183	0.000185
H	-0.971565	-2.021979	0.000036
H	-1.129224	2.298687	-0.000571
H	-3.619215	2.109934	-0.000387
O	-6.452933	-0.015165	0.079886
H	-5.485612	0.072720	-0.045660
H	-6.795390	0.874576	0.203097
O	-5.661561	-2.509973	1.153325
H	-6.124995	-1.807448	0.672308
H	-5.423354	-2.117313	1.997931

Energy -26.327819 (Hartree)

Py-Cluster-SOLVATOR-DFT

Atom	X	Y	Z (Angstrom)
C	-3.064039	-0.797879	0.257715
C	-1.682171	-0.982335	0.241259
C	-0.862673	0.116439	-0.001456
C	-1.454293	1.359092	-0.220735
C	-2.843517	1.445371	-0.186414
N	-3.629461	0.394618	0.048405
H	0.224825	0.007284	-0.022221
H	-3.753950	-1.633714	0.437284
H	-1.264521	-1.976169	0.414290
H	-0.852834	2.248919	-0.417615
H	-3.346944	2.402992	-0.357454
O	-6.373095	-0.132639	0.144921
H	-5.452835	0.208254	0.058299
H	-6.708665	0.292070	0.940124
O	-5.634157	-2.745195	0.685890
H	-6.018538	-1.893252	0.392355
H	-5.701975	-2.693056	1.644051

Energy 0.000000 (Hartree)

Pd(II)

Atom	X	Y	Z (Angstrom)
Pd	0.045522	-0.922556	0.046315
P	-2.145585	-0.662701	-0.018454
P	2.236489	-0.662626	-0.018819
C	0.047871	1.181223	0.259715
H	0.044709	1.374906	1.341579
H	0.926439	1.650683	-0.201132
H	-0.827753	1.650478	-0.206907
C	3.113098	0.132527	1.359028
H	2.680809	-0.179256	2.318650
H	4.166337	-0.192914	1.328720
H	3.097599	1.230575	1.277588
C	3.023258	0.050246	-1.493276
H	4.060981	-0.321132	-1.535507
H	2.501900	-0.276242	-2.402359
H	3.061636	1.149824	-1.453579
C	-3.021611	0.133836	1.358977
H	-4.075703	-0.188697	1.327802
H	-2.590796	-0.179171	2.318862
H	-3.003060	1.231871	1.277727
C	-2.932313	0.049530	-1.493287
H	-2.411473	-0.278048	-2.402277
H	-3.970405	-0.320861	-1.535027
H	-2.969665	1.149179	-1.454340

Energy 0.000000 (Hartree)

Acetonitrile.xyz

Atom	X	Y	Z (Angstrom)
C	-2.074068	-0.213339	0.028965

H	-1.713456	-1.250715	0.018922
H	-1.713460	0.296628	-0.874466
H	-3.172318	-0.219131	0.018890
C	-1.587401	0.474920	1.221046
N	-1.202416	1.019290	2.163996

Energy 0.000000 (Hartree)

Pd-Cluster-COMPLAX-Best

Atom	X	Y	Z (Angstrom)
Pd	0.045522	-0.922556	0.046315
P	-2.145585	-0.662701	-0.018454
P	2.236489	-0.662626	-0.018819
C	0.047871	1.181223	0.259715
H	0.044709	1.374906	1.341579
H	0.926439	1.650683	-0.201132
H	-0.827753	1.650478	-0.206907
C	3.113098	0.132527	1.359028
H	2.680809	-0.179256	2.318650
H	4.166337	-0.192914	1.328720
H	3.097599	1.230576	1.277588
C	3.023258	0.050246	-1.493276
H	4.060981	-0.321132	-1.535507
H	2.501900	-0.276242	-2.402359
H	3.061636	1.149824	-1.453579
C	-3.021611	0.133836	1.358977
H	-4.075703	-0.188697	1.327802
H	-2.590796	-0.179171	2.318862
H	-3.003060	1.231871	1.277727
C	-2.932313	0.049530	-1.493287
H	-2.411473	-0.278048	-2.402277
H	-3.970405	-0.320861	-1.535027
H	-2.969665	1.149179	-1.454340
C	0.268107	-5.758969	-0.400426
H	1.302820	-6.004223	-0.667986
H	-0.402737	-6.113767	-1.187699
H	0.022886	-6.268060	0.541395
C	0.147203	-4.340948	-0.301609
N	0.050507	-3.192115	-0.232277

Energy -36.333908 (Hartree)

Pd-Cluster-COMPLAX-DFT

Atom	X	Y	Z (Angstrom)
Pd	0.060605	-1.376229	1.176128
P	-1.862304	-0.482643	0.300664
P	2.006546	-0.461453	0.378566
C	0.022742	0.058195	2.593884
H	0.912500	-0.142042	3.212591
H	0.020043	1.106305	2.276836
H	-0.886459	-0.156802	3.178475
C	2.075150	1.364241	0.494248
H	2.180779	1.691057	1.536550
H	2.974262	1.689703	-0.053849
H	1.204107	1.861192	0.036582
C	1.909643	-0.658056	-1.443027
H	2.897133	-0.379125	-1.847155
H	1.719308	-1.704538	-1.714834
H	1.158819	-0.008567	-1.918970
C	-1.953846	1.342395	0.410185
H	-2.831452	1.658070	-0.177059
H	-2.107279	1.670054	1.446229
H	-1.068760	1.846793	-0.010838
C	-1.690745	-0.680401	-1.515288
H	-1.473831	-1.724315	-1.777085

H	-2.665681	-0.417277	-1.958713
H	-0.931764	-0.020108	-1.962785
C	0.137252	-5.611588	-1.012756
H	1.139283	-5.827981	-1.407161
H	-0.599162	-5.680798	-1.824721
H	-0.112354	-6.343303	-0.232307
C	0.115020	-4.274297	-0.443958
N	0.097513	-3.215891	0.010964

Energy 0.000000 (Hartree)

Pd-Cluster-ABCluster-Best

Atom	X	Y	Z (Angstrom)
Pd	4.115487	3.876375	3.931305
P	5.338803	4.618804	2.118442
P	2.556411	2.435384	3.007608
C	2.931986	5.529833	3.993623
H	2.261140	5.730842	3.161366
H	2.347999	5.371142	4.912987
H	3.609356	6.381852	4.130596
C	0.892023	3.215897	2.931956
H	0.852314	3.978854	2.157497
H	0.174404	2.438838	2.665678
H	0.590667	3.648393	3.885834
C	2.285911	1.456920	4.550422
H	1.467185	0.751423	4.404314
H	3.183600	0.889372	4.800633
H	2.025854	2.116501	5.385078
C	5.788847	2.976799	1.416009
H	6.373499	3.124241	0.508367
H	6.400832	2.409212	2.117631
H	4.898019	2.395910	1.162050
C	4.126316	5.110959	0.827143
H	3.676879	6.073740	1.060921
H	4.648321	5.208709	-0.123996
H	3.343055	4.357892	0.714980
C	7.410673	1.625245	6.344120
H	7.899715	0.741997	5.931942
H	6.973781	1.375520	7.311441
H	8.150799	2.414668	6.479314
C	6.394662	2.068015	5.455151
N	5.578327	2.397541	4.717254

Energy 0.000000 (Hartree)

Pd-Cluster-ABCluster-DFT

Atom	X	Y	Z (Angstrom)
Pd	4.129794	4.074653	4.171825
P	5.173701	4.694975	2.224689
P	2.737754	2.396694	3.329920
C	2.720022	5.473879	3.882916
H	2.170384	5.501900	2.937117
H	2.014730	5.293765	4.713192
H	3.243948	6.430971	4.041144
C	1.150986	3.042267	2.669928
H	1.306339	3.609896	1.741847
H	0.558701	2.149984	2.404753
H	0.565309	3.643884	3.379471
C	2.149892	1.788794	4.970452
H	1.472819	0.940441	4.772322
H	2.986160	1.406943	5.572750
H	1.586209	2.536759	5.549113
C	5.835709	3.117011	1.565396
H	6.396399	3.363012	0.647926
H	6.536138	2.665069	2.279705

H	5.047090	2.391025	1.314117
C	4.019224	5.139694	0.875041
H	3.477575	6.066578	1.102913
H	4.641939	5.327731	-0.015448
H	3.310168	4.336091	0.623407
C	7.530181	1.277732	6.071419
H	7.718453	0.397689	5.441588
H	7.209673	0.949586	7.069526
H	8.452989	1.866799	6.161838
C	6.489149	2.094594	5.469725
N	5.665427	2.742461	4.991074

Energy -1142.187901 (Hartree)

Pd-Cluster-OGOLEM-Best

Atom	X	Y	Z (Angstrom)
Pd	0.247408	-0.984636	0.615881
P	1.363066	0.832660	1.625800
P	-1.181934	-2.047594	-0.925693
C	1.932519	-1.616978	-0.348387
H	2.800275	-1.540924	0.316087
H	1.820995	-2.665574	-0.646006
H	2.121712	-1.014426	-1.244052
C	-0.387207	-2.683608	-2.470445
H	0.107211	-3.633443	-2.272927
H	-1.165191	-2.851948	-3.215417
H	0.340694	-1.977273	-2.870069
C	-1.734776	-0.408163	-1.599750
H	-2.457062	-0.549287	-2.404453
H	-2.206507	0.165620	-0.798507
H	-0.871850	0.146544	-1.987213
C	3.040773	1.229633	0.957685
H	3.292810	2.252198	1.239998
H	3.783458	0.562348	1.391343
H	3.072173	1.142742	-0.128556
C	0.401761	2.036657	0.589779
H	-0.663612	1.916355	0.799237
H	0.693476	3.060892	0.823736
H	0.588568	1.845530	-0.473361
C	-0.532946	-2.248525	3.165957
H	0.213823	-2.835713	2.603926
H	-1.330494	-2.909541	3.506335
H	-0.049567	-1.784703	4.027834
C	-1.084539	-1.210915	2.313420
N	-1.687807	-0.366947	1.794773

Energy -96212.612200 (Hartree)

Pd-Cluster-OGOLEM-DFT

Atom	X	Y	Z (Angstrom)
Pd	1.100517	-1.673315	0.606498
P	1.896350	0.463552	0.863408
P	-0.264258	-1.980913	-1.211747
C	2.684114	-2.068613	-0.579204
H	3.554803	-1.985122	0.091201
H	2.548712	-3.121249	-0.876248
H	2.841387	-1.450223	-1.468919
C	0.447539	-1.374855	-2.785267
H	1.271494	-2.017272	-3.121182
H	-0.350509	-1.425622	-3.543661
H	0.791671	-0.328959	-2.730981
C	-1.621613	-0.765943	-0.993296
H	-2.380199	-0.994776	-1.760494
H	-2.094496	-0.878197	-0.008935
H	-1.307016	0.279392	-1.137122

```
  C     2.684942     1.148172    -0.639654
  H     2.876911     2.216380    -0.448121
  H     3.649503     0.663526    -0.836822
  H     2.045281     1.078493    -1.534554
  C     0.397132     1.518859     0.938351
  H    -0.294721     1.164525     1.713416
  H     0.733226     2.530036     1.222975
  H    -0.133279     1.602329    -0.022959
  C    -2.078001    -1.832750     4.155916
  H    -2.003091    -2.847565     4.569840
  H    -3.108063    -1.651737     3.820343
  H    -1.812107    -1.102462     4.932196
  C    -1.166807    -1.701675     3.031110
  N    -0.442196    -1.603035     2.140863
Energy            0.000000  (Hartree)
```

Pd-Cluster-CREST-Best

```
 Atom      X             Y             Z (Angstrom)
 Pd     0.039445    -0.806495     0.172507
  P     2.369187    -0.544064    -0.299806
  P    -2.276297    -0.614831    -0.391069
  C    -0.012031     0.942310     1.244582
  H    -0.038753     1.826371     0.599308
  H    -0.901809     0.948343     1.886531
  H     0.870210     1.007548     1.892569
  C    -3.124045     0.978870     0.002895
  H    -2.638204     1.796116    -0.527267
  H    -4.157085     0.908862    -0.338435
  H    -3.121947     1.191795     1.072035
  C    -3.107938    -1.670236     0.883363
  H    -4.176759    -1.725639     0.675396
  H    -2.688139    -2.676811     0.853773
  H    -2.960396    -1.254398     1.882593
  C     3.157991     1.068560     0.137907
  H     4.200585     1.034698    -0.179118
  H     2.660431     1.878750    -0.392294
  H     3.124369     1.265217     1.209670
  C     3.178848    -1.587451     0.998530
  H     2.788995    -2.604978     0.943483
  H     4.256284    -1.611075     0.833459
  H     2.979604    -1.185181     1.994368
  C     0.107462    -2.253040    -2.593375
  H     1.006608    -2.662286    -3.053886
  H     0.105500    -1.159451    -2.703156
  H    -0.778277    -2.666806    -3.075495
  C     0.091307    -2.593951    -1.192088
  N     0.082473    -3.062135    -0.129753
Energy          -36.663717  (Hartree)
```

Pd-Cluster-CREST-DFT

```
 Atom      X             Y             Z (Angstrom)
 Pd     0.030296    -1.074698     0.526807
  P     1.733727     0.260318    -0.345121
  P    -1.687929     0.193624    -0.412616
  C    -0.020222    -0.042273     2.243825
  H    -0.037346     1.051436     2.213591
  H    -0.924568    -0.413239     2.756754
  H     0.874727    -0.383856     2.792736
  C    -2.014943     1.752141     0.496903
  H    -1.187921     2.463648     0.366353
  H    -2.896726     2.195064     0.002453
  H    -2.244720     1.631588     1.564689
  C    -3.041915    -0.852327     0.276684
```

H	-3.983094	-0.451812	-0.138470
H	-2.955686	-1.892880	-0.068424
H	-3.122201	-0.827574	1.373936
C	2.012311	1.795602	0.619125
H	2.898081	2.266517	0.158820
H	1.177768	2.498687	0.491058
H	2.218897	1.646273	1.687923
C	3.094686	-0.779122	0.341146
H	3.041572	-1.808430	-0.041953
H	4.036492	-0.342864	-0.034421
H	3.146511	-0.791298	1.440476
C	0.162110	-4.307549	-3.025198
H	1.080902	-4.906563	-2.966693
H	0.146003	-3.756022	-3.974996
H	-0.710346	-4.973048	-2.975033
C	0.121999	-3.366267	-1.918563
N	0.089151	-2.616460	-1.044566

Energy 0.000000 (Hartree)

Pd-Cluster-SOLVATOR-Best

Atom	X	Y	Z (Angstrom)
Pd	0.045522	-0.922556	0.046315
P	-2.145585	-0.662701	-0.018454
P	2.236489	-0.662626	-0.018819
C	0.047871	1.181223	0.259715
H	0.044709	1.374906	1.341579
H	0.926439	1.650683	-0.201132
H	-0.827753	1.650478	-0.206907
C	3.113098	0.132527	1.359028
H	2.680809	-0.179256	2.318650
H	4.166337	-0.192914	1.328720
H	3.097599	1.230575	1.277588
C	3.023258	0.050246	-1.493276
H	4.060981	-0.321132	-1.535507
H	2.501900	-0.276242	-2.402359
H	3.061636	1.149824	-1.453579
C	-3.021611	0.133836	1.358977
H	-4.075703	-0.188697	1.327802
H	-2.590796	-0.179171	2.318862
H	-3.003060	1.231871	1.277727
C	-2.932313	0.049530	-1.493287
H	-2.411473	-0.278048	-2.402277
H	-3.970405	-0.320861	-1.535027
H	-2.969665	1.149179	-1.454340
C	0.999669	-4.587594	-3.000319
C	0.451973	-3.483087	-2.288852
N	-0.000559	-2.593155	-1.723671
H	1.905374	-4.277560	-3.521728
H	1.242197	-5.385406	-2.297942
H	0.270609	-4.950670	-3.724620

Energy 0.000000 (Hartree)

Pd-Cluster-SOLVATOR-DFT

Atom	X	Y	Z (Angstrom)
Pd	0.275238	-1.550931	0.440899
P	-1.750233	-0.585419	-0.036192
P	2.106008	-0.250914	-0.028867
C	0.197840	-0.698368	2.267315
H	-0.664325	-1.178564	2.757981
H	1.128163	-1.021911	2.761707
H	0.102406	0.390406	2.333834
C	2.047203	1.419995	0.716564
H	2.183692	1.372982	1.804430

```
  H     2.889457     1.992496     0.295283
  H     1.119078     1.966737     0.482797
  C     1.931074     0.188741    -1.801454
  H     2.874485     0.672162    -2.105741
  H     1.801225    -0.710758    -2.417371
  H     1.112914     0.897674    -2.002115
  C    -1.974129     1.077498     0.696071
  H    -2.900291     1.497037     0.270661
  H    -2.101778     1.016728     1.784376
  H    -1.150846     1.770642     0.457673
  C    -1.654234    -0.136690    -1.812608
  H    -1.376540    -1.006695    -2.421901
  H    -2.666272     0.178557    -2.117204
  H    -0.967446     0.697918    -2.022176
  C     0.556493    -4.763061    -3.070150
  C     0.466811    -3.706826    -2.075743
  N     0.391966    -2.870720    -1.286553
  H     1.580452    -5.160128    -3.093208
  H    -0.141247    -5.570504    -2.809282
  H     0.297220    -4.363051    -4.059723
Energy             0.000000  (Hartree)
```

Acetate_Ion

```
 Atom       X            Y             Z  (Angstrom)
  C    -0.752701    -1.229758     0.021647
  H    -0.421965    -2.281201     0.009721
  H    -0.423730    -0.713357    -0.894952
  H    -1.857617    -1.229202     0.025108
  C    -0.238663    -0.488109     1.306276
  O    -0.080944     0.741751     1.180384
  O    -0.084201    -1.211644     2.309240
Energy             0.000000  (Hartree)
```

Acetate-Cluster-COMPLAX-Best

```
 Atom       X            Y             Z  (Angstrom)
  C    -0.752701    -1.229758     0.021647
  H    -0.421965    -2.281201     0.009721
  H    -0.423730    -0.713357    -0.894952
  H    -1.857617    -1.229202     0.025108
  C    -0.238663    -0.488109     1.306276
  O    -0.080944     0.741751     1.180384
  O    -0.084201    -1.211644     2.309240
  O    -2.403668    -0.074297     2.628722
  H    -1.485131    -0.080132     2.343469
  H    -2.392691     0.494162     3.404517
  O    -0.208511     2.393921     0.739442
  H     0.032875     2.209697    -0.173196
  H    -0.770298     3.171452     0.668970
  O     2.014082    -0.207997     2.525192
  H     1.570826    -0.272151     1.674003
  H     2.900065     0.086216     2.293680
  O     1.725597     2.200468     2.002655
  H     0.807450     2.152492     2.285176
  H     1.660241     2.306540     1.048925
Energy             0.000000  (Hartree)
```

Acetate-Cluster-COMPLAX-DFT

```
 Atom       X            Y             Z  (Angstrom)
  C    -0.959638    -0.960036    -0.117534
  H    -0.624567    -1.962495    -0.420814
  H    -0.814131    -0.216339    -0.911409
  H    -2.040775    -1.033827     0.090931
  C    -0.264487    -0.538345     1.185213
```

O	0.047203	0.684708	1.282409
O	-0.110080	-1.403699	2.066206
O	-1.476121	-0.741034	4.365285
H	-0.976757	-0.992488	3.565414
H	-0.952511	0.008619	4.666195
O	-0.412574	2.062378	-1.018111
H	-0.156377	1.567803	-0.205360
H	-1.370528	2.070278	-0.919403
O	2.291020	-1.110993	3.658486
H	1.510224	-1.376316	3.146272
H	2.194029	-0.143878	3.639332
O	1.695058	1.623768	3.157388
H	1.003846	1.329314	2.513081
H	2.310960	2.095337	2.589742

Energy -533.744309 (Hartree)

Acetate-Cluster-ABCluster-Best

Atom	X	Y	Z (Angstrom)
C	2.924275	3.323230	0.819314
H	2.608485	2.368932	1.234331
H	3.663930	3.143346	0.040101
H	2.078044	3.851620	0.386679
C	3.578530	4.188435	1.919120
O	3.508569	5.425452	1.780810
O	4.137880	3.584979	2.855413
O	5.534227	7.056787	2.635192
H	4.966281	7.383396	3.349077
H	4.920520	6.464805	2.150052
O	6.541708	4.752420	3.811859
H	5.839698	4.201040	3.421489
H	6.379238	5.631952	3.421659
O	4.189375	4.643199	5.381772
H	5.139176	4.811017	5.287467
H	3.986123	4.180866	4.540927
O	3.157871	6.919078	4.171942
H	3.445597	6.204519	4.770954
H	3.090706	6.473295	3.308430

Energy 0.000000 (Hartree)

Acetate-Cluster-ABCluster-DFT

Atom	X	Y	Z (Angstrom)
C	2.938463	3.323020	0.811940
H	2.592212	2.359135	1.210531
H	3.724181	3.119467	0.066317
H	2.120203	3.855492	0.307368
C	3.540387	4.185441	1.927862
O	3.469675	5.428060	1.774035
O	4.079967	3.570406	2.878577
O	5.534729	7.087646	2.650237
H	4.883987	7.282840	3.357026
H	4.963691	6.517244	2.097930
O	6.473366	4.777833	3.835302
H	5.790152	4.229996	3.403439
H	6.292540	5.656988	3.435576
O	4.235652	4.666840	5.433017
H	5.165050	4.816345	5.161310
H	3.944997	4.165945	4.644117
O	3.262097	6.924536	4.182568
H	3.559430	6.193222	4.767549
H	3.119457	6.447910	3.341888

Energy 0.000000 (Hartree)

Acetate-Cluster-OGOLEM-Best

Atom	X	Y	Z (Angstrom)
C	0.708844	0.414520	-1.522816
H	0.874609	0.939896	-0.587165
H	-0.244160	-0.105912	-1.541974
H	0.865781	1.041028	-2.390930
C	1.858548	-0.785653	-1.549879
O	2.574017	-0.606211	-2.497614
O	1.702554	-1.538643	-0.624183
O	-2.176520	-0.578586	-0.628023
H	-3.048655	-0.972004	-0.577242
H	-2.275834	0.353193	-0.330935
O	0.171654	1.407852	1.507080
H	0.017750	0.425778	1.533877
H	0.297264	1.680003	2.416903
O	-2.155373	1.961758	0.268984
H	-1.321297	1.852939	0.778280
H	-2.018360	2.704638	-0.320156
O	-0.331628	-1.179228	1.331557
H	0.427621	-1.560411	0.871841
H	-1.040973	-1.122868	0.663476

Energy -89829.534400 (Hartree)

Acetate-Cluster-OGOLEM-DFT

Atom	X	Y	Z (Angstrom)
C	0.928629	0.565475	-1.520336
H	0.998791	1.092409	-0.555049
H	-0.107675	0.201288	-1.620346
H	1.160825	1.259023	-2.341034
C	1.909954	-0.636471	-1.531344
O	2.816099	-0.632181	-2.364190
O	1.679855	-1.524876	-0.654286
O	-2.925513	-0.547248	0.028176
H	-2.862702	-0.582524	0.988132
H	-2.642871	0.374401	-0.165644
O	-0.242057	1.260890	1.680987
H	-0.185717	0.285677	1.396567
H	-0.390108	1.227096	2.628866
O	-1.908662	1.991753	-0.262002
H	-1.323947	1.851818	0.523827
H	-1.284064	1.921442	-0.993410
O	-0.255073	-1.195816	0.925754
H	0.551519	-1.357320	0.290064
H	-1.031440	-1.222751	0.346349

Energy 0.000000 (Hartree)

Acetate-Cluster-CREST-Best

Atom	X	Y	Z (Angstrom)
C	1.296129	-0.238813	-0.701499
H	1.302025	0.847869	-0.632173
H	2.078458	-0.615260	-1.347664
H	1.286930	-0.726076	0.277619
C	-0.141786	-0.609923	-1.360303
O	0.045668	-1.202318	-2.401786
O	-1.085654	-0.226216	-0.711006
O	-0.680520	-0.493405	3.605699
H	-0.507517	0.273396	4.160063
H	-1.386621	-0.224095	2.984059
O	-2.578432	0.265906	1.758959
H	-3.385305	-0.246509	1.646247
H	-2.120510	0.246484	0.907523
O	1.316397	-1.562515	2.063448
H	0.635462	-1.166592	2.646954
H	1.255552	-2.512882	2.196098

O	1.298010	2.925875	-0.315320
H	0.802321	3.626300	-0.750055
H	1.444996	3.205506	0.594173

Energy -34.246163 (Hartree)

Acetate-Cluster-CREST-DFT

Atom	X	Y	Z (Angstrom)
C	1.283783	-1.038714	-1.925976
H	2.202273	-0.536406	-1.583978
H	1.085143	-0.768957	-2.971985
H	1.458504	-2.121809	-1.838284
C	0.120110	-0.625085	-1.018460
O	-0.814798	0.024612	-1.509400
O	0.224050	-0.994296	0.192626
O	-1.118746	0.480594	3.766872
H	-0.697193	1.305435	3.450402
H	-1.698138	0.302164	3.008321
O	-1.770767	0.476517	1.050148
H	-1.924297	0.615275	0.098638
H	-1.039472	-0.189702	0.929547
O	1.402042	-0.189485	2.513348
H	0.586689	-0.237916	3.044373
H	1.130080	-0.571128	1.651922
O	0.154598	2.262654	2.079573
H	-0.534168	1.820656	1.543114
H	0.825909	1.552321	2.140237

Energy 0.000000 (Hartree)

Acetate-Cluster-SOLVATOR-Best

Atom	X	Y	Z (Angstrom)
C	-0.752701	-1.229758	0.021647
H	-0.421965	-2.281201	0.009721
H	-0.423730	-0.713357	-0.894952
H	-1.857617	-1.229202	0.025108
C	-0.238663	-0.488109	1.306276
O	-0.080944	0.741751	1.180384
O	-0.084201	-1.211644	2.309240
O	0.645757	1.441296	3.801224
H	0.589682	0.569256	4.206140
H	0.429531	1.288930	2.861047
O	0.204203	-3.969648	2.403988
H	0.009526	-3.014421	2.437396
H	1.156461	-4.041004	2.519840
O	-1.028498	2.500862	-0.650159
H	-1.504202	3.094827	-0.062544
H	-0.612967	1.843938	-0.057677
O	-2.003272	0.774022	-2.608037
H	-2.947800	0.847136	-2.444880
H	-1.587316	1.435509	-2.026955

Energy 0.000000 (Hartree)

Acetate-Cluster-SOLVATOR-DFT

Atom	X	Y	Z (Angstrom)
C	-1.106136	-0.965930	0.356052
H	-0.993951	-2.055385	0.426317
H	-0.758851	-0.596161	-0.619349
H	-2.175655	-0.710623	0.438534
C	-0.349556	-0.275526	1.495613
O	-0.274581	0.981287	1.454829
O	0.126546	-0.995839	2.401611
O	1.127838	1.182877	3.926213
H	0.955393	0.246581	3.732142
H	0.711325	1.535840	3.123155

O	0.076282	-3.661362	1.911247
H	0.023718	-2.723648	2.218014
H	0.838946	-3.613029	1.327016
O	-1.370690	2.223069	-0.560034
H	-2.276100	2.235297	-0.236934
H	-0.879169	1.779421	0.196820
O	-1.174859	0.592741	-2.762286
H	-1.814773	-0.061598	-2.468660
H	-1.194442	1.241170	-2.023493

Energy 0.000000 (Hartree)

S7References

(1) Harris, C. R.; Millman, K. J.; van der Walt, S. J.; Gommers, R.; Virtanen, P.; Cournapeau, D.; Wieser, E.; Taylor, J.; Berg, S.; Smith, N. J.; et al. Array programming with NumPy. *Nature* **2020**, *585* (7825), 357-362. DOI: 10.1038/s41586-020-2649-2.

(2) *colorama: Cross-platform colored terminal text*; 2024. https://pypi.org/project/colorama/ (accessed.

(3) *tabulate: Pretty-print tabular data*; 2024. https://pypi.org/project/tabulate/ (accessed.

(4) da Costa-Luis, C. O. tqdm: A Fast, Extensible Progress Meter for Python and CLI. *Journal of Open Source Software* **2019**, *4* (37). DOI: 10.21105/joss.01277.

(5) Bannwarth, C.; Caldeweyher, E.; Ehlert, S.; Hansen, A.; Pracht, P.; Seibert, J.; Spicher, S.; Grimme, S. Extended tight-binding quantum chemistry methods. *WIREs Computational Molecular Science* **2020**, *11* (2). DOI: 10.1002/wcms.1493.

(6) Lin, Y.-S.; Li, G.-D.; Mao, S.-P.; Chai, J.-D. Long-Range Corrected Hybrid Density Functionals with Improved Dispersion Corrections. *Journal of Chemical Theory and Computation* **2012**, *9* (1), 263-272. DOI: 10.1021/ct300715s.

(7) Weigend, F.; Ahlrichs, R. Balanced basis sets of split valence, triple zeta valence and quadruple zeta valence quality for H to Rn: Design and assessment of accuracy. *Physical Chemistry Chemical Physics* **2005**, *7* (18). DOI: 10.1039/b508541a.

(8) Neese, F. Software Update: The ORCA Program System—Version 6.0. *WIREs Computational Molecular Science* **2025**, *15* (2). DOI: 10.1002/wcms.70019.
